# Single-cell transcriptomics reveals immune infiltrate in sepsis

**DOI:** 10.3389/fphar.2023.1133145

**Published:** 2023-04-11

**Authors:** Xusheng Tu, He Huang, Shilei Xu, Caifei Li, Shaoning Luo

**Affiliations:** ^1^ Department of Emergency Medicine, Third Affiliated Hospital of Sun Yat-sen University, Guangzhou, China; ^2^ Department of General Surgery, The Third Affiliated Hospital of Sun Yat-sen University, Guangzhou, China; ^3^ Third Affiliated Hospital of Sun Yat-sen University, Guangzhou, China

**Keywords:** immune diseases, sepsis, machine learning, single cell, inflammation

## Abstract

Immune cells and immune microenvironment play important in the evolution of sepsis. This study aimed to explore hub genes related to the abundance of immune cell infiltration in sepsis. The GEOquery package is used to download and organize data from the GEO database. A total of 61 differentially expressed genes (DEGs) between sepsis samples and normal samples were obtained through the ‘limma’ package. T cells, natural killer (NK) cells, monocytes, megakaryocytes, dendritic cells (DCs), and B cells formed six distinct clusters on the t-distributed stochastic neighbor embedding (t-SNE) plot generated using the Seurat R package. Gene set enrichment analysis (GSEA) enrichment analysis showed that sepsis samples and normal samples were related to Neutrophil Degranulation, Modulators of Tcr Signaling and T Cell Activation, IL 17 Pathway, T Cell Receptor Signaling Pathway, Ctl Pathway, Immunoregulatory Interactions Between a Lymphoid and A Non-Lymphoid Cell. GO analysis and KEGG analysis of immune-related genes showed that the intersection genes were mainly associated with Immune-related signaling pathways. Seven hub genes (CD28, CD3D, CD2, CD4, IL7R, LCK, and CD3E) were screened using Maximal Clique Centrality, Maximum neighborhood component, and Density of Maximum Neighborhood Component algorithms. The lower expression of the six hub genes (CD28, CD3D, CD4, IL7R, LCK, and CD3E) was observed in sepsis samples. We observed the significant difference of several immune cell between sepsis samples and control samples. Finally, we carried out *in vivo* animal experiments, including Western blotting, flow cytometry, Elisa, and qPCR assays to detect the concentration and the expression of several immune factors.

## 1 Introduction

Sepsis is a common life-threatening syndrome that is a major cause of morbidity and mortality worldwide (Islam et al., 2019). Sepsis is a condition in which the patient’s ability to control infection is compromised and the infection continues to spread, leading to multiple organ failure and even life-threatening disease (Napolitano, 2018). Due to the lack of effective means to control sepsis, patients with sepsis rapidly deteriorate and die. Even with the rapid development and use of antibiotics today, the prevalence of sepsis patients is still slowly increasing with the rise of drug-resistant strains. Sepsis has become a major global health burden due to rising treatment costs and a significant increase in the length and number of hospitalizations (Islam et al., 2019). Rapid assessment of sepsis severity and mortality risk and timely adjustment of treatment strategies will play an important role in reducing the overall mortality and cost burden of sepsis.

Once sepsis occurs, a variety of inflammatory factors, bacterial products, and other into the bloodstream through various organs. The basic pathogenesis and mechanism of sepsis are complicated, especially the immune mechanism plays an important role in the onset and development of sepsis. For example, apoptosis of immune cells (T and B lymphocytes) and myeloid-derived suppressor cells (MDSCs) is a major contributor to the development of immunosuppression in patients with sepsis (Schrijver et al., 2019; Cheng et al., 2020). The expression of the chemokine CX3CL1 is increased in patients with septic shock, while the decrease of its receptor CX3CR1 is directly associated with poor prognosis (Chen et al., 2020a). In addition, regulation of immune checkpoints also plays an important role in sepsis-induced immunosuppression. For example, increased expression of programmed cell death protein-1 (PD-1) leads to T-cell apoptosis, lymphocytopenia, and impaired phagocytosis of leukocytes to varying degrees (van der Poll et al., 2021). Therefore, the role of immune cells and the immune microenvironment in the development of sepsis. In the development of sepsis.

During sepsis-induced organ damage, the lung is the first organ to be affected (Sadowitz et al., 2011). The alveolar epithelium of the lung tissue is the primary target of harmful substances in the process of acute lung injury caused by sepsis. Immune factors play a pivotal role in the development of acute lung injury (Kumar, 2020). Studies indicate that the primary inflammatory factors involved in acute lung injury are tumor necrosis factor and interleukin 6. Additionally, neutrophils are also known to have a crucial function in the initial stages of the injury. The interaction between different cells and factors triggers a cascade of inflammatory responses through a positive feedback mechanism, leading to a waterfall effect that damages and destroys the lung tissue. Consequently, the ability to resist microbial attack is compromised, rendering this the most critical aspect of acute lung injury.

The human immune system constantly faces the challenge of combatting diverse pathogenic invaders. Infection is the outcome of complex interactions between pathogenic microorganisms and human immune cells. The transmission rate of pathogenic microorganisms is influenced by multiple factors, including environmental conditions, pathogen-host interactions, and the host immune cell response mechanism (Chen et al., 2020b). The application of single-cell sequencing (scRNA-seq) technology to analyze blood, sputum and other infected tissues of patients has demonstrated its efficacy in elucidating the immune landscape and corresponding signaling pathways during infection. Moreover, this technology enables the discovery of novel immune cell subsets and biomarkers connected to infectious diseases (Cho et al., 2020; Darden et al., 2021; Wang et al., 2021). Immune cells play a crucial role in initiating host defenses against pathogenic infections. The immune cell atlas provides an overview of immune cell structure under specific conditions, either physiological or infectious, and crucial insights into the pathogenesis of infectious diseases. Single-cell sequencing technology has simplified and standardized the construction of immune cell maps, enabling the identification of global immune cell changes that occur during infections (Han et al., 2020; Wilk et al., 2020; Zhang et al., 2020; Li et al., 2021; Ren et al., 2021).

## 2 Materials and methods

### 2.1 Data download and data preprocessing

We used GEOquery package of R software (version 4.0.0, http://rproject.org/) (Davis and Meltzer, 2007) to download the expression profile of reliable sample source for patients with sepsis-induced lung injury (SILI) from GSE28750 dataset from GEO database (https://www.ncbi.nlm.nih.gov/geo/) (Sutherland et al., 2011). The chip platform was based on the GPL570 [HG-U133_Plus_2] Affymetrix Human Genome U133 Plus 2.0 Array, and the samples in the dataset were all derived from *Homo sapiens*. There was a total of 10 sepsis samples and 20 normal samples in GSE28750 dataset. We read the original data of GSE28750 by “affy” package (Gautier et al., 2004), obtained the gene expression matrix of the data set, and normalized the data by “limma” package. Setting species as *H. sapiens*, the single cell sequencing (scRNA-seq) of sepsis was downloaded from Single Cell Portal database (https://singlecell.broadinstitute.org/single_cell). The Seurat R package (Version 4.0) was used to process single-cell data, and 29 samples were included to create Seurat objects for our analysis (Butler et al., 2018; Stuart et al., 2019; Hao et al., 2021; Stuart et al., 2021). We used the doubletFinder_v3 function to remove the two-cell effect and the LogNormalize method to normalize the data. After controlling for the relationship between average expression and dispersion, highly variable genes were identified in individual cells. Whereafter, variable genes were identified as inputs, Principal Component Analysis (PCA) were carried out and significant principal components based on the ElbowPlot function were finally identified (Supplementary Figure S1). According, the “elbow criterion,” the first 15 principal components were selected as statistically significant inputs of the t-Distributed Neighbor Embedding (t-SNE).

### 2.2 Differentially expressed genes screening

We selected differentially expressed genes (DEGs) of GSE28750 through the limma package (Ritchie et al., 2015). The ggplot2 package was used to map the volcano of DEGs (Ito and Murphy, 2013) and the pheatmap package was used to map the heatmap of DEGs (Ito and Murphy, 2013) to display the distribution of DEGs. In addition, we used the removeBatchEffect function in the limma package to remove the batch effect and DEGs met the requirements of the adj.p.value < 0.05 and |log2FC|>1. We downloaded and collated lists of immune-related genes from Pubmed and MSigDB databases, and then manually searched the original literature and reviews containing these genes (Reyes et al., 2020). Finally, after excluding extraneous genes and adding other reported genes, DEGs of sepsis were screened out and intersected with immune-related genes to obtain immune-related differentially expressed genes (IRDEGs).

### 2.3 Analysis of single cell clustering and annotation

First, we used the FindClusters function to cluster the cells and identify the cell types of the clusters.In order to verify the annotation of single cell, HumanPrimaryCellAtlasData was used to annotate the cell types through SingleR (Version 1.8.1) (Aran et al., 2019). In order to annotate cells more accurately, we sorted out marker genes of all kinds of cells according to previously published literature to identify cell types, and further searched for differential marker genes among cell subsets.

### 2.4 Trajectory analysis for cell subsets

Cell differentiation was inferred using the Monocle package of R (version 2.22.0) (Trapnell et al., 2014). An integrated gene expression matrix from each cell type was first exported from the Seurat object to Monocle to construct the cell data set. We used the variable genes defined by dispersionTable function and then sequenced the cells using the setOrderingFilter function. Finally, the Darter method was used for dimensionality reduction, and the orderCells function was used to estimate the arrangement of cells along the trajectory. Based on the clustering characteristics and marker gene analysis, the differentiation time locus of cell subsets in single cell data set was obtained.

### 2.5 Gene set variation analysis

Gene set variation analysis (GSVA) is a non-parametric, unsupervised algorithm (Hänzelmann et al., 2013). We analyzed the data based on the GSVA package (version 1.42.0) of R. GSVA algorithm transformed gene expression data from the expression matrix of a single gene as a feature to the expression matrix of a specific gene set as a feature (Hänzelmann et al., 2013). The gene set corresponding to each feature was calculated using rank statistics similar to K-S test, and the expression matrix was converted into Enrichment Score (ES) matrix for feature (Hänzelmann et al., 2013). GSVA enrichment score corresponding to each sample for each feature could be obtained, which would facilitate further statistical analysis (Hänzelmann et al., 2013).

### 2.6 Mice for *in vivo* animal experiments

In an animal experiment, 40 male C57BL6 mice aged 6–8 weeks were employed (Purchased from Guangdong Medical Laboratory Animal Center). After 7-day adaptive feeding, these rats were divided into four groups: sham group (*n* = 10), sham + anti-IL-7 group (*n* = 10), sepsis group and sepsis + anti-IL-7 group (*n* = 10). The rats in sepsis group were anesthetized by isoflurane, and a 1-cm midline incision was made along the abdominal line. The abdominal cavity was then opened layer by layer to find the ileum. A tight ligature was made at 1 cm from the distal end of ileum, and two punctures were made 0.5 cm away from the distal end with a 7-gauge needle. A small amount of feces was squeezed into the abdominal cavity. The ileum was placed back to its normal position in the abdominal cavity and the abdomen was then closed in two layers. After the operation, 1 mL of pre-heated physiological saline was injected subcutaneously to each mouse for fluid supplementation, and 10 mg/kg of tramadol hydrochloride was injected subcutaneously as an analgesic. It was injected once every 12 h within 48 h after the operation. Except for the blind intestinal ligation and perforation, the other operations were the same as those of the surgical group in the sham operation group. 48 h later, the septic rats showed symptoms of depression, decreased activity, and decreased appetite. After the modelling was completed, IL-7 (2.5 μg/day) was administered i.p. daily for 5 days, and death was performed on the sixth day. The spleen tissues were collected after the mice were sacrificed. Recombinant human IL-7 was purchased from Abcam Company and diluted with sterile distilled water. The animal experiments were performed following the National Institutes of Health Guide for the Care and Use of Laboratory Animals and approved by the Animals Care and Use Committee of Sun Yat-sen University (Guangzhou, China).

### 2.7 Western blotting assays

We extracted proteins from the spleen of mice. Tissue samples were lysed in RIPA buffer adding protease and phosphatase inhibitors. Protein concentrations of the supernatants were determined after centrifugation using BCA Protein Assay Kit (Beyotime). We performed Western blotting assays according to the following steps: 1) Proteins were separated by electrophoresis on NuPAGE^®^ 4%–12% Bis-Tris Gel (Invitrogen, Life Technologies) for 40 min at 200 V and transferred onto polyvinylidene difluoride (PVDF) membranes (Invitrogen, Thermo Fisher; 30 V, 1 h); 2) The membranes were blocked with Blocking Solution of the Western Breeze^®^ Chromogenic Western blot Immunodetection Kit (Invitrogen, Thermo Fisher) for 30 min on a rotary shaker at room temperature; 3) The membranes were then incubated overnight at 4°C with the following primary antibodies: GAPDH (ProteinTech, Wuhan, China, 1:5000), IL-7R (Abcam, 1:1000), and CD4 (Abcam, 1:1000). Goat anti-rabbit IgG or goat anti-mouse IgG (Invitrogen) were used as the secondary antibodies; 4)Target proteins were visualized by Molecular Imager ChemiDoc XRS System (Bio-Rad) with super Electro-Chemi-Luminescence (ECL) plus kits (Beyotime); 5) The protein bands were analyzed with Image-Pro Plus 6.0 software (Media Cybernetics); 6) Relative protein expression levels were expressed as the ratio of the band intensity of the target protein to that of GAPDH.

### 2.8 Apoptosis quantifying

Apoptosis was quantified by flow cytometry using the TUNEL assay. Flow cytometric analysis (50,000–100,000 events/sample) was per_x0002_formed on FACScan. Tissues were then rinsed with PBS and treated with a TUNEL reaction mixture according to the kit instructions. After rinsing in PBS, the converter-POD was added to the tissue, covered with a glass slide or sealing film, and reacted in a dark wet box for 30 min.

### 2.9 Elisa assays

The procedure Elisa (Invitrogen, Thermo Fisher) assays procedure were roughly as follows: dilute the corresponding protein with a coated solution, seal the enzymic label plate with plastic film, and place overnight in a 4°C refrigerator. The enzymic label plate was evenly wrapped at room temperature, washed with PBST lotion for 3 times, added BSA sealer, sealed with plastic film, and placed in a 37°C water bath for 1 h. They were incubated with primary and secondary antibodies successively, washed with PBST lotion for 3 times, and added TMB and 3% H_2_O_2_ at 37°C for 10 min of dark color development. Add H2SO4 to terminate the color rendering. Then, the optical density (OD) of each well was measured at the wavelength of 450 nm by enzyme-labeled instrument.

## 3 Results

### 3.1 Flow chart for our study

**FIGURE 1:**
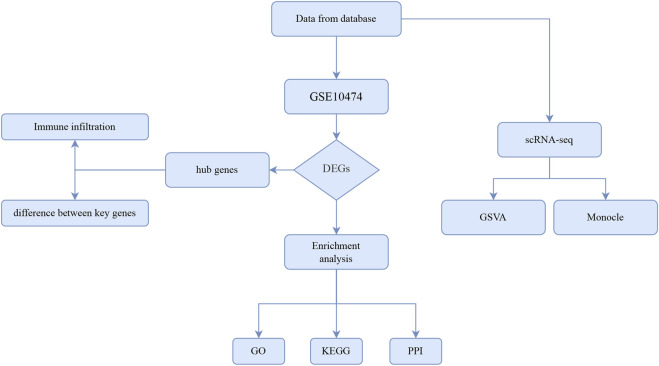
The overall technical route of this study.

We first drew up the overall technical route of this study, which was shown in Figure 1.

### 3.2 Data standardization and immune-related gene screening

**FIGURE 2:**
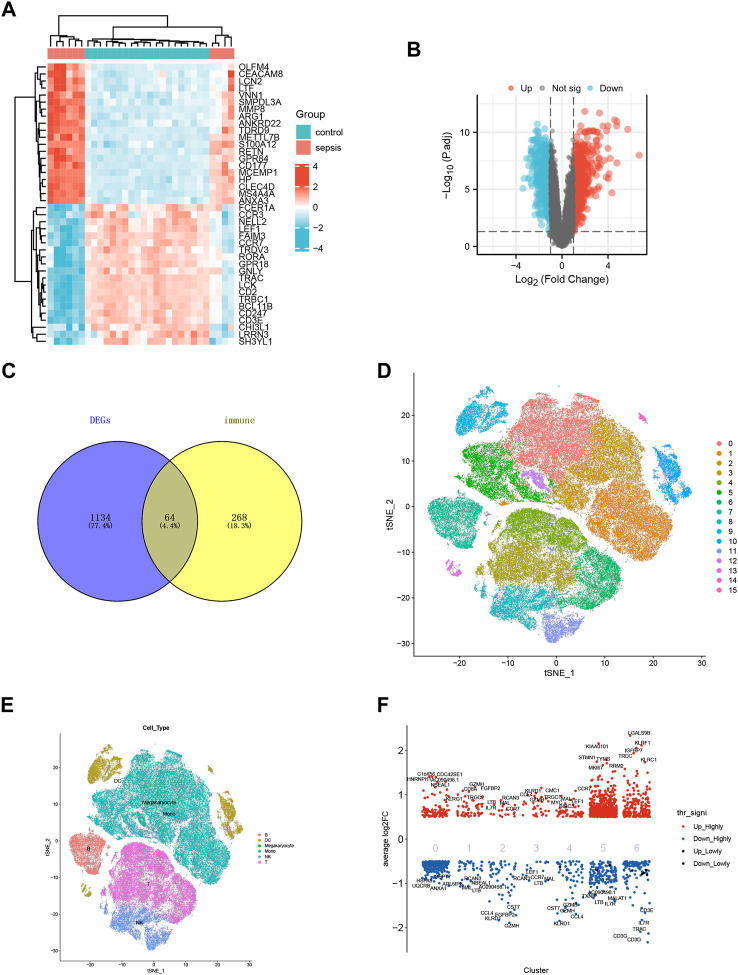
Data standardization and immune-related gene screening. **(A)** Heat map for GSE28750. Blue represents the control group and red represents the sepsis-induced lung injury (SILI) group. **(B)**Volcano map for GSE10474. Red represents upregulated differential genes, blue downregulated differential genes, and black represents undifferentiated genes. **(C)** Venn diagram showing intersection for differential genes of sepsis and immune-related genes. **(D)** tSNE diagram for single cell samples of sepsis. 0–15 representing different cell clusters. **(E)** tSNE diagram for single cell samples with different colors representing T cells, B cells, Mono cells, Megakaryocyte cells, NK cells, and DC cells respectively. **(F)** Single cell heat map of differentially expressed genes, with red representing highly expressed genes and blue representing low-expressed genes.

The data of DEGs were summarized and sorted out according to GEO data platform, and the data of the analyzed sepsis samples and normal samples were compared. We removed the probes corresponding to multiple molecules from one probe, and when the probes corresponding to the same molecule were encountered, only the probe with the largest signal value was retained. After data preprocessing for GSE28750, a total of 21,655 molecules were filtered, of which 1198 molecules met |log2(FC) |>1 and p.adj<0.05. Under the above threshold (|log2(FC) |>1 and p.adj<0.05), compared with the control group, 599 genes were upregulated, and 599 genes were downregulated in the sepsis group. We used R software to extract the mutual differentially expressed genes from the gene expression matrix, as shown in the heat map (Figure 2A) and volcano map (Figure 2B). We manually searched the original literature and reviews containing immune-related genes, excluded irrelevant genes and added other reported genes, and intersected them with the list of differentially expressed genes with *p*-value less than 0.05, thus obtaining a total of 64 genes, as shown in the Venn diagram (Figure 2C). For the unsupervised analysis, we plotted the single-cell transcriptome on t-distributed Stochastic Neighbour Embedding (tSNE) diagram (Figures 2D, E). T cells, NK cells, Mono cells, Megakaryocyte cells, DC cells, and B formed six separate clusters on the tSNE diagram (Figure 2E). Single-cell differential expression analysis (SCDE) identified the genes that are differentially expressed between different cell types (Figure 2F).

### 3.3 GSEA enrichment analysis of GSE28750 dataset

**FIGURE 3:**
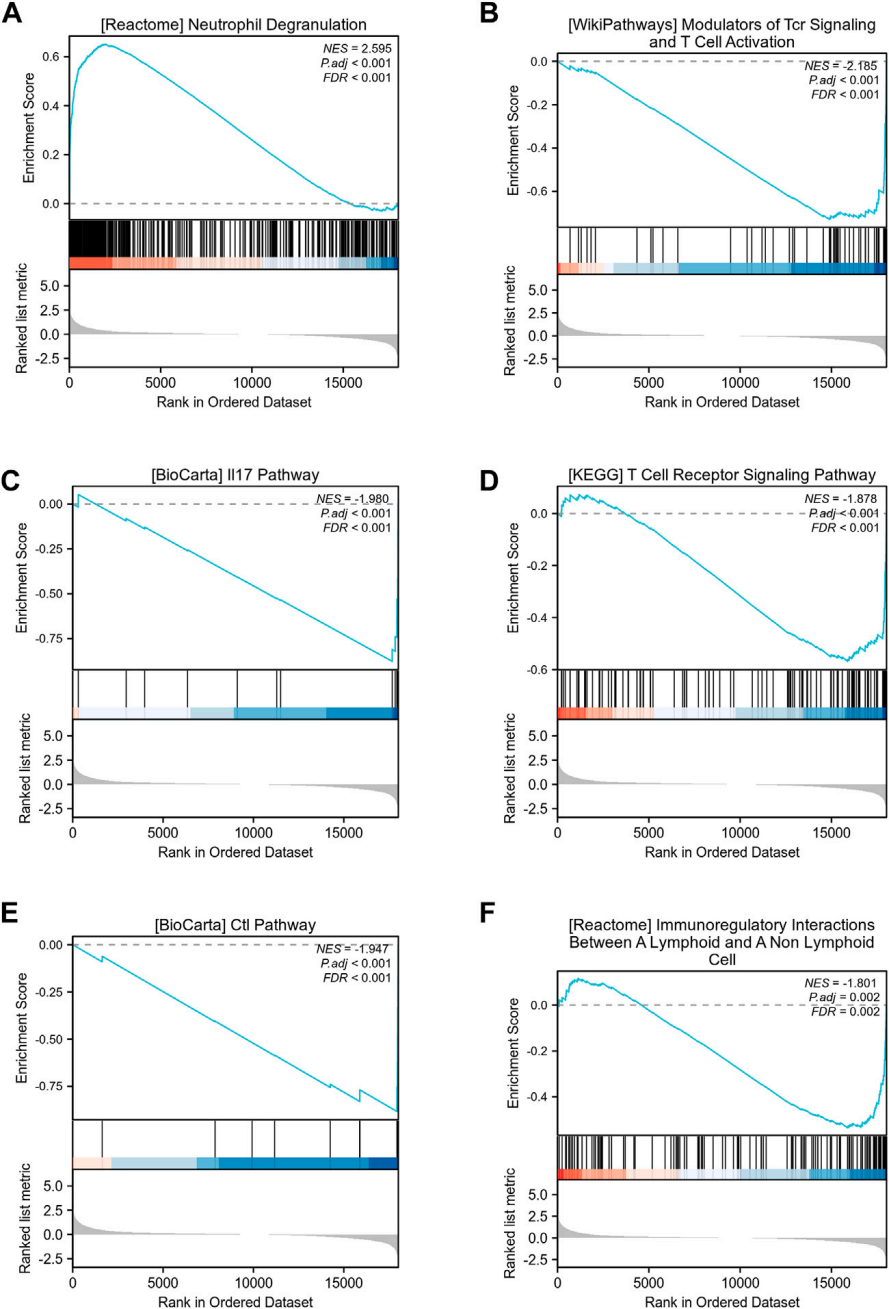
GSEA enrichment analysis of GSE28750 dataset. **(A)** GSEA enrichment analysis of data sets in Neutrophil Degranulation. **(B)** GSEA enrichment analysis of data sets in Modulators of Tcr Signaling and T Cell Activation. **(C)** GSEA enrichment analysis of data sets in IL 17 Pathway. **(D)** GSEA enrichment analysis of data sets in T Cell Receptor Signaling Pathway. **(E)** GSEA enrichment analysis of data sets in Ctl Pathway. **(F)** GSEA enrichment analysis of data sets in Immunoregulatory Interactions Between A Lymphoid and A Non-Lymphoid Cell.

To identify differences in biological processes between sepsis samples and normal samples, based on gene expression profile data from the GSE28750 dataset, we carried out gene set enrichment analysis (GSEA) using R packages (Table 1; Figures 3A–F). GESA results showed that sepsis samples and normal samples were related to the biological phenomena as below: Neutrophil Degranulation (Figure 3A), Modulators of Tcr Signaling and T Cell Activation (Figure 3B), IL 17 Pathway (Figure 3C), T Cell Receptor Signaling Pathway (Figure 3D), Ctl Pathway (Figure 3E), Immunoregulatory Interactions Between A Lymphoid and A Non-Lymphoid Cell (Figure 3F).

**TABLE 1:** The primer sequences in PCR analysis.

**Symbol**	**Sequences (5′-3′)**
IL-7R-F	TTG​GAC​TTC​CTC​CCC​TGA​TCC
IL-7R-R	TCG​ATG​CTG​ACC​ATT​AGA​ACA​C
CD4-F	TGC​CTC​AGT​ATG​CTG​GCT​CT
CD4-R	GAG​ACC​TTT​GCC​TCC​TTG​TTC
GAPDH-F	GGA​GCG​AGA​TCC​CTC​CAA​AAT
GAPDH-R	GGC​TGT​TGT​CAT​ACT​TCT​CAT​GG

### 3.4 Enrichment analysis for immune-related genes and single cell

**FIGURE 4:**
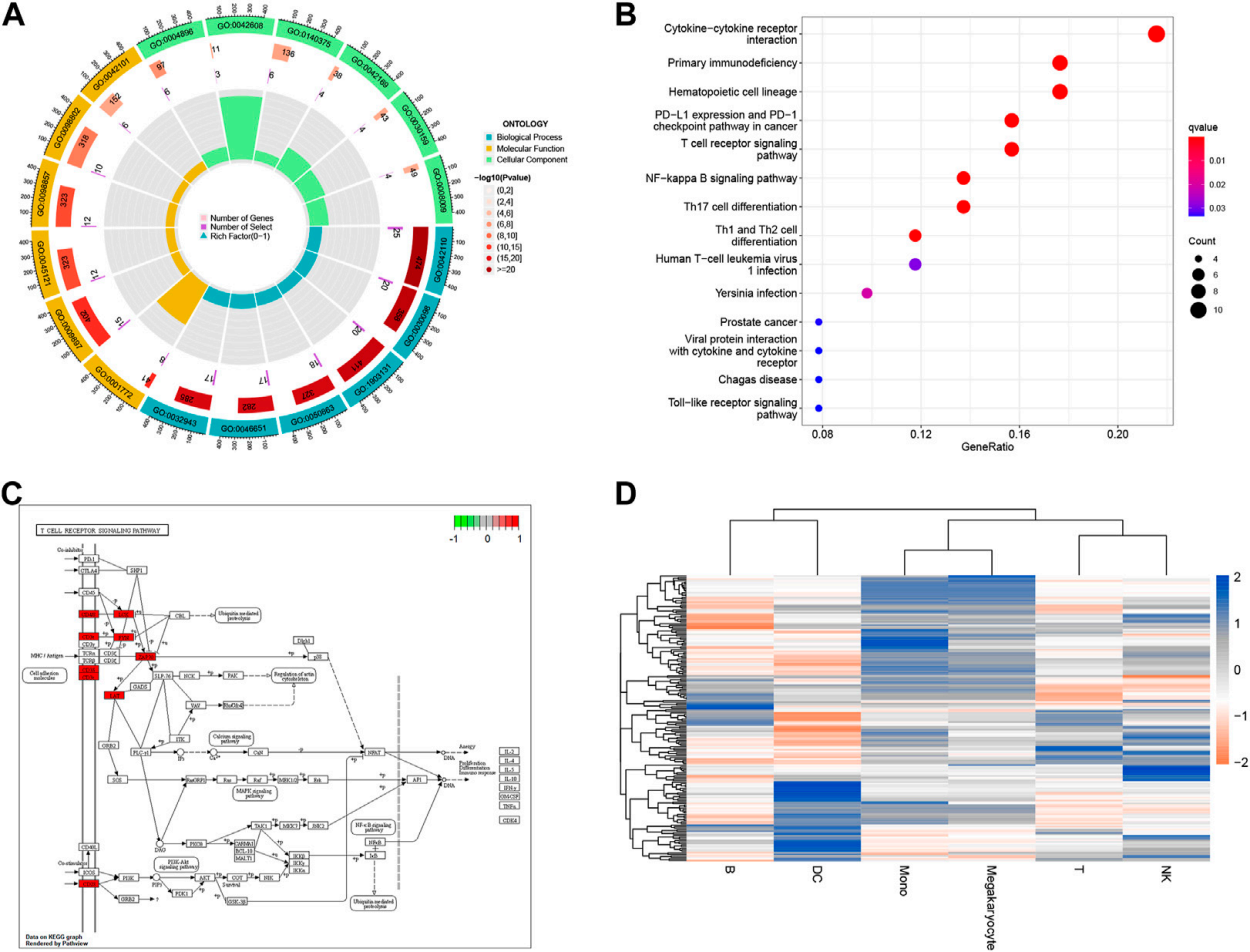
Enrichment analysis for immune-related genes and Single cell. **(A)** Chord plot for enrichment analysis of GO biological function. Blue, yellow and green respectively represent different enrichment functions, while red represents *p*-value. The darker the color, the smaller the *p*-value. **(B)** Bar graph for KEGG pathway enrichment analysis. The x-horizontal axis represents the proportion of enriched genes, and the color of the bar represents the *p*-value: the redder the color, the smaller the *p*-value, and the bluer the color, the larger the *p*-value. **(C)** Dot plot for KEGG pathway enrichment analysis. The *X*-axis represents the proportion of enriched genes, and the darker the color, the smaller the *p*-value. **(D)** Visualization of GSVA pathway for Single cell. Red squares represent negative correlation, blue squares represent positive correlation.

We performed GO analysis of immune-related genes, the results showed that intersection genes were mainly associated with T cell receptor binding, immune receptor activity, SH2 domain binding, signaling receptor complex adaptor activity, chemokine activity and other biological phenomena (Figure 4A). The results showed that intersection genes were mainly associated with T cell receptor binding, immune receptor activity, SH2 domain binding, signaling receptor complex adaptor activity, chemokine activity and other biological phenomena. The results of KEGG analysis showed that intersection genes were enriched in Primary immunodeficiency, Hematopoietic cell lineage, PD-L1 expression and PD-1 checkpoint pathway in cancer, T cell receptor signaling pathway, PD-L1 expression and PD-1 checkpoint pathway in cancer, T cell receptor signaling pathway, and Cytokine-cytokine receptor interaction pathways (Figures 4B, C). Single-cell GSVA analysis showed that the function of immunorelated cells in sepsis was mainly enriched in ABC transporters, Acute myeloid leukemia, Adherens Junction, Adipocytokine Signaling Pathway, Alanine Aspartate and Glutamate Metabolism (Figure 4D). Finally, we identified the key genes to be mainly concentrated in T cells after Aucell scoring, Ucell scoring, ssgsea scoring and singscoring (Supplementary Figure S2).

### 3.5 Hub genes screening based on PPI network of immune-related genes

**FIGURE 5:**
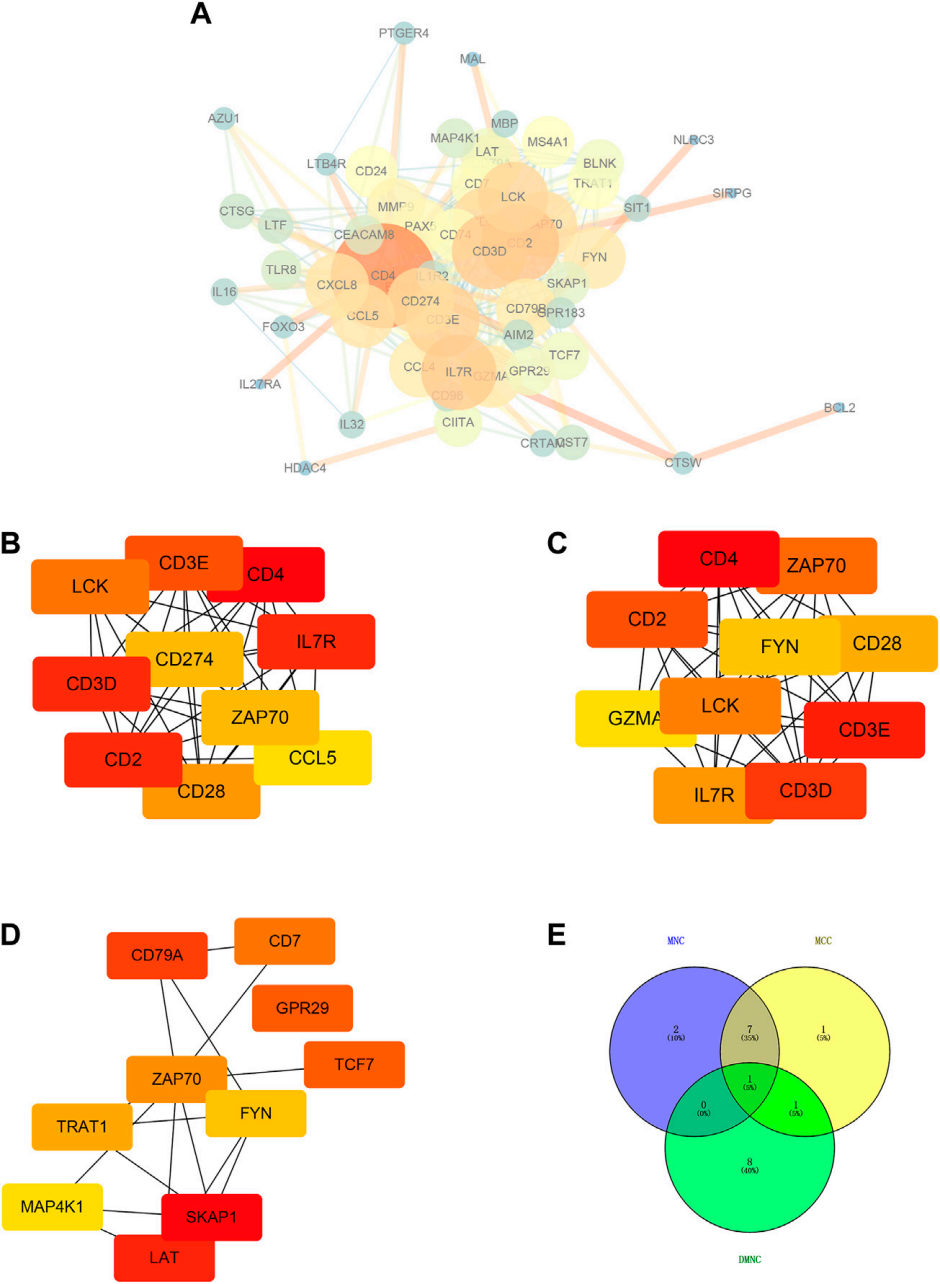
Hub genes screening based on PPI network of immune-related genes. **(A)** Protein-protein interaction (PPI) analysis based on STRING tool. Cytoscape software was used for visualization. The larger the genes involved, the larger the degree of nodes, and the thicker the lines, the larger the number of betweenness. **(B)** Top ten hub genes (LCK, CD3E, CD4, CD3D, CD274, IL7R, ZAP70, CD2, CCL5, and CD28) screened based on MNC algorithm. **(C)** Top ten hub genes (CD4, ZAP70, CD2, FYN, CD28, GZMA, LCK, CD3E, IL7R, and CD3D) screened based on MCC algorithm. **(D)** Top ten hub genes (CD79A, CD7, GPR29, ZAP70, TCF7, TRAT1, FYN, MAP4K1, SKAP1, LAT) screened based on DMNC algorithm. **(E)** Intersection of hub genes screened by the above three algorithms.

We used STRING tool to conduct protein-protein interaction (PPI) analysis for immune-related genes and visualized the number of interactions between each immune-related gene. The larger the genes involved, the larger the degree of nodes, and the thicker the lines, the larger the number of betweenness (Figure 5A). Subsequently, we utilized the Maximal Clique Centrality (MCC), Maximum neighborhood component (MNC), and Density of Maximum Neighborhood Component (DMNC) algorithms based on cytoHubba plug-in of Cytoscape software to screened seven hub genes (Figures 5B–E). Intersection of hub genes screened by the above three algorithms was selected and the screening results described the seven hub genes as CD28, CD3D, CD2, CD4, IL7R, LCK and CD3E (Figures 5B–E).

### 3.6 Differential analysis of hub gene

**FIGURE 6:**
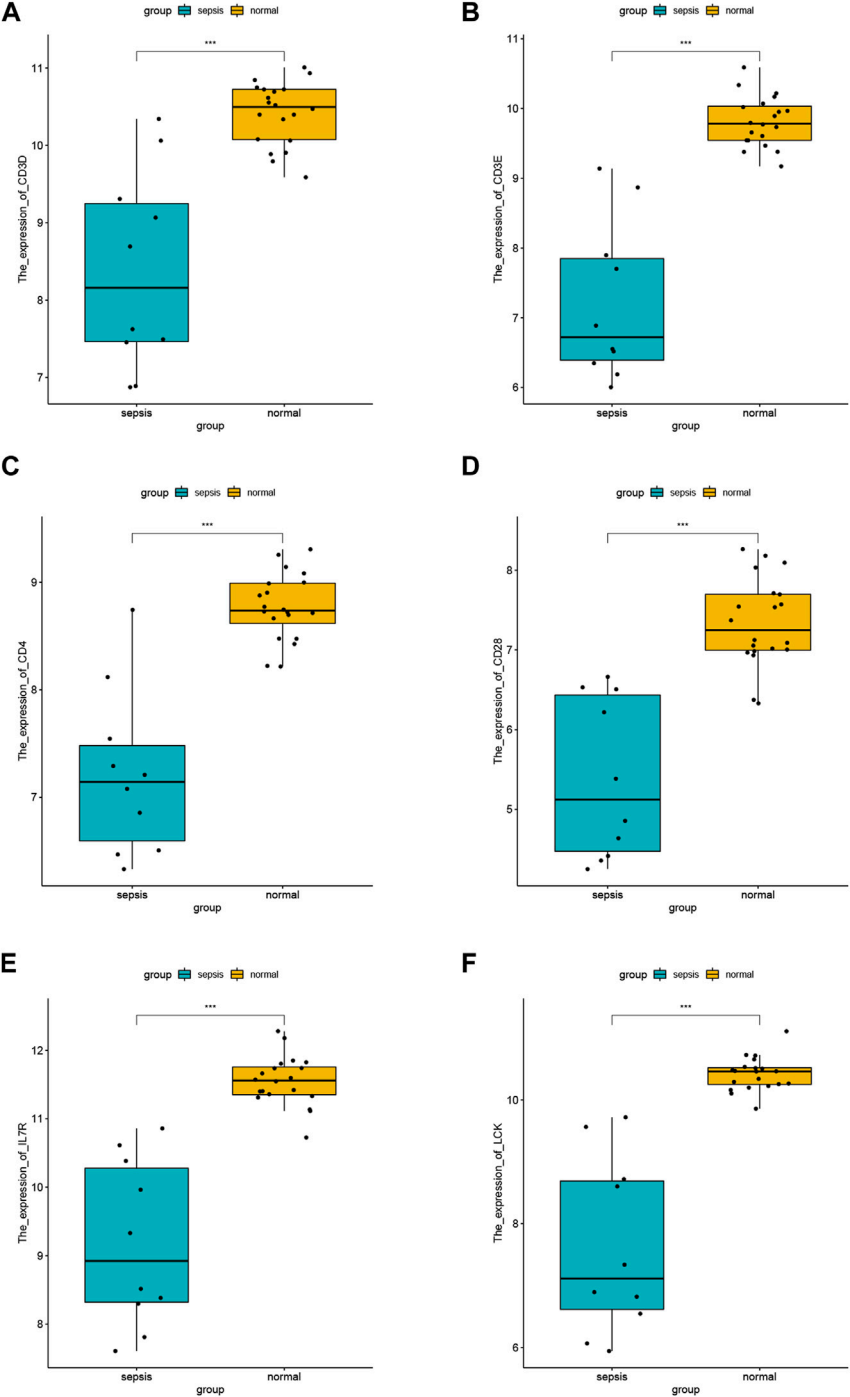
Differential analysis of hub gene. **(A)** Expression difference of CD3D in GSE28750 between sepsis group and control group. **(B)** Expression difference of CD3E in GSE28750 between sepsis group and control group. **(C)** Expression difference of CD4 in GSE28750 between sepsis group and control group. **(D)** Expression difference of CD28 in GSE28750 between sepsis group and control group. **(E)** Expression difference of IL7R in GSE28750 between sepsis group and control group. **(F)** Expression difference of LCK in GSE28750 between sepsis group and control group. *p < 0.05; ∗∗*p* < 0.01; ∗∗∗*p* < 0.001.

We visualized the expression values of the other six hub genes except CD2 in the dataset GSE28750 and showed the differences (Figures 6A–F). The results showed the expression level of CD3D (Figure 6A), CD3E (Figure 6B), CD4 (Figure 6C), CD28 (Figure 6D), IL7R (Figure 6E), and LCK (Figure 6F) was statistically different between sepsis and normal groups. The lower expression of the hub genes was observed in sepsis samples, which was consistent in all the six hub genes (Figures 6A–F).

### 3.7 Immunoinfiltration analysis of sepsis using GSE28750 dataset

**FIGURE 7:**
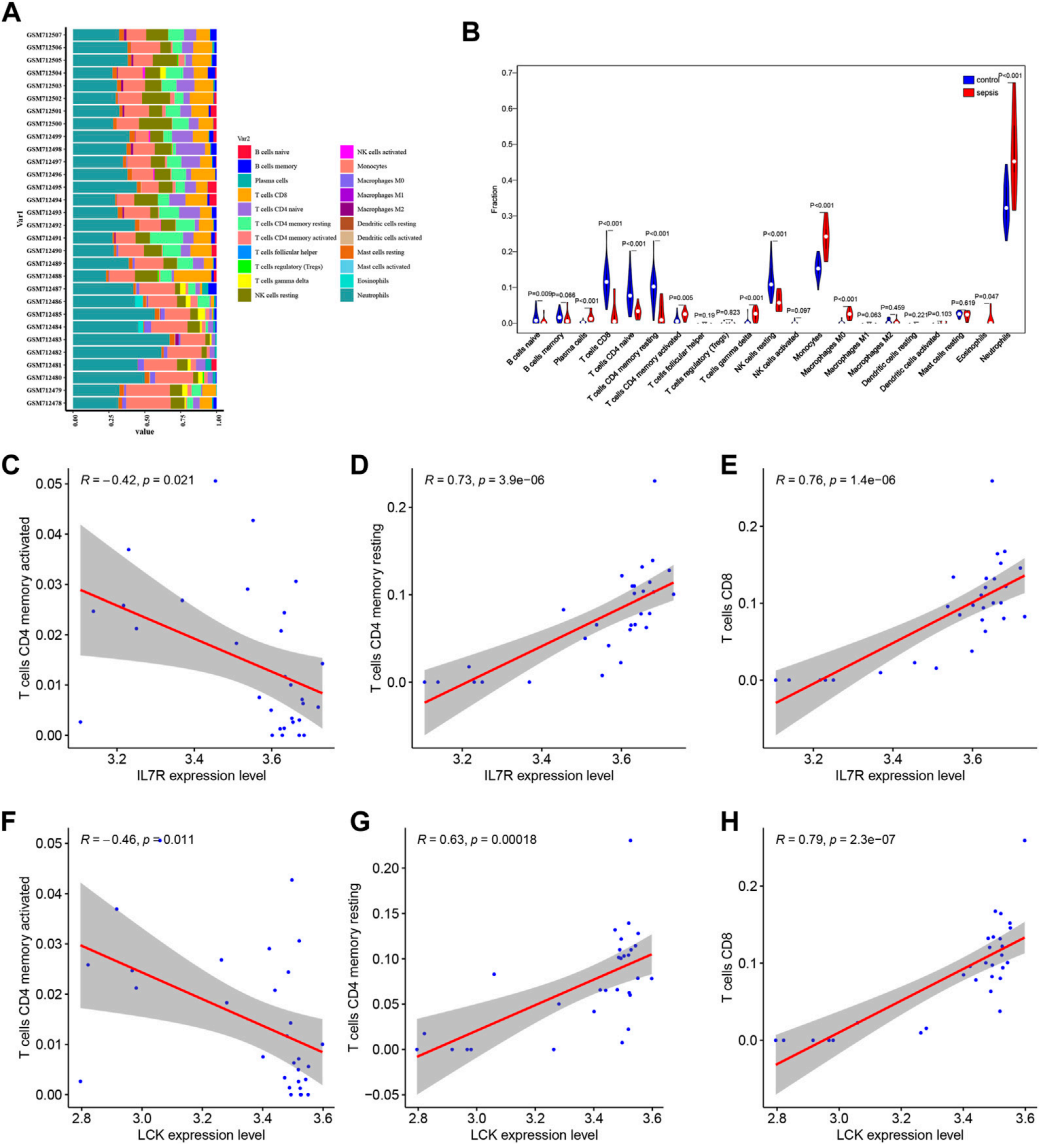
Analysis and visualization of immune cell infiltration. **(A)** Proportion of immune cell infiltration in GSE28750 data set. **(B)** Box diagram of immune cell infiltration in GSE28750 data set. Red was the sepsis group and blue was the control group. **(C–F)** Correlation between hub genes and immune cells. IL7R was negatively correlated with T cells CD4 memory activated **(C)**. IL7R was positively correlated with T cells CD4 memory resting **(D)**. IL7R was positively correlated with T cells CD8 **(E)**. LCK was negatively correlated with T cells CD4 memory activated **(F)**. LCK was positively correlated with T cells CD4 memory resting **(G)**. LCK was positively correlated with T cells CD8 **(H)**.

Based on CIBERSORT algorithm, the immune cell infiltration analysis was conducted on the sepsis samples and control samples in the GSE28750 data, and the proportion of immune cells in each sample was analyzed to obtain the proportion of 22 kinds of immune cells in the sepsis samples and control samples (Figure 7A). Further, we carried out an intergroup comparison of 22 types of immune cells between sepsis samples and control samples (Figure 7B). We observed the significant difference of B cells naïve, plasma cells, T cells CD8, T cells CD4 naïve, T cells CD4 memory resting, T cells CD4 memory activated, T cells gamma delta, NK cells resting, Monocytes, Macrophages M0, Eosinophils, Neutrophils cells between sepsis samples and control samples (Figure 7B). Further, the correlation between hub genes and immune cells were analyzed according to gene expression and the abundance of corresponding immune cells, and the interaction relationship was visualized. It could be seen that IL7R was negatively correlated with T cells CD4 memory activated (Figure 7C), while positively correlated with T cells CD4 memory resting (Figure 7D) and T cells CD8 (Figure 7E). LCK was negatively correlated with T cells CD4 memory activated (Figure 7F), while positively correlated with T cells CD4 memory resting (Figure 7G) and T cells CD8 (Figure 7H).

### 3.8 Trajectory analysis for T cells

**FIGURE 8:**
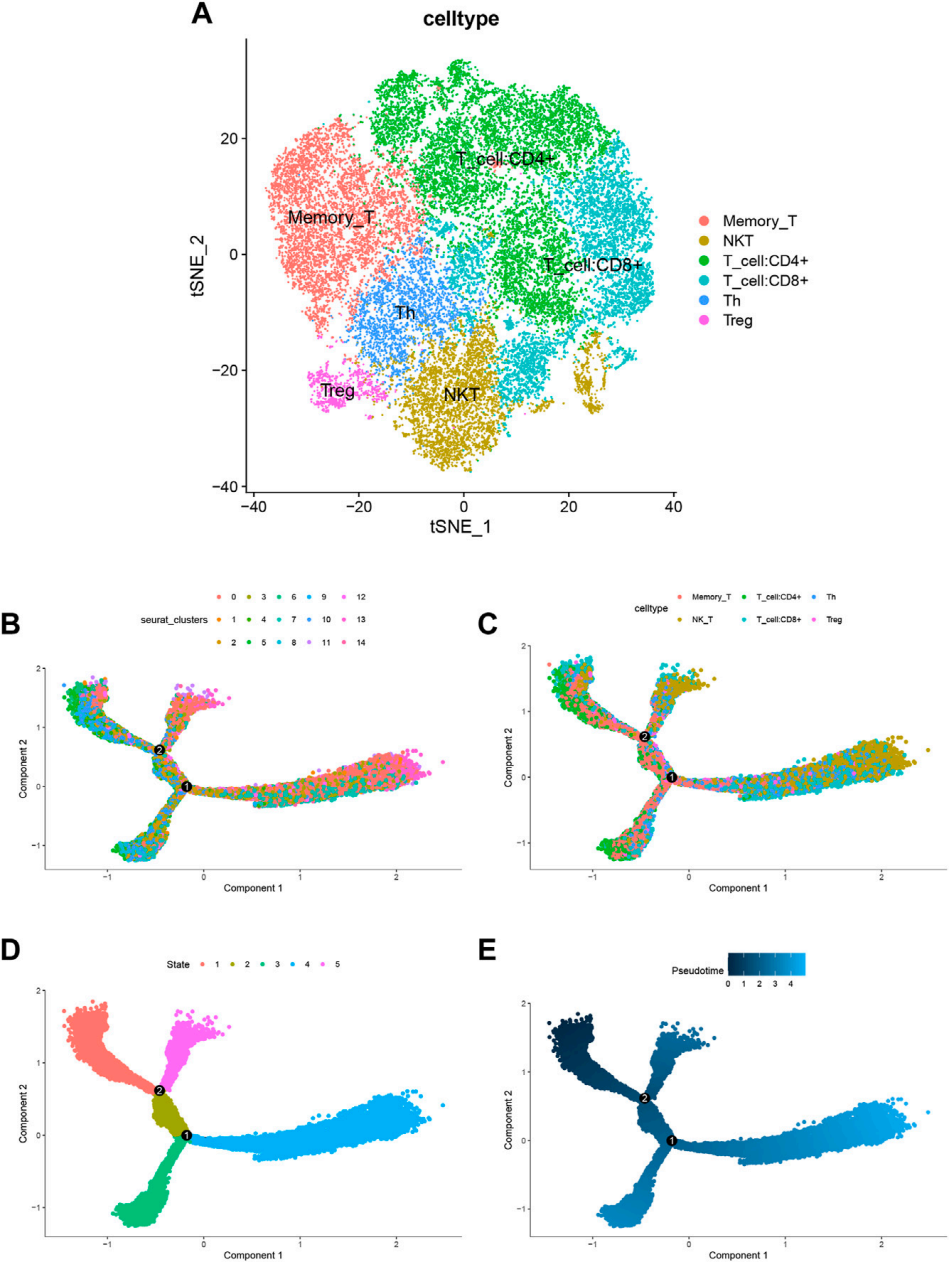
Trajectory analysis for T cells. **(A)** tSNE diagram for T cells. T cells differentiate into Memory T cells, NK T cells, CD4^+^ T cells, CD8^+^ T cells, Th T cells and Treg T cells. **(B–E)** Pseudotime analysis for T cells.

According to 3.4 analysis results showed that the differential gene score in T cells was higher. Next, we were able to capture the differentiation of T cells into Memory T cells, NK T cells, CD4^+^ T cells, CD8^+^ T cells, Th T cells and Treg T cells (Figure 8A) in the Trajectory analysis for T cells. The results of pseudotime analysis were displayed in Figures 8B–E.

### 3.9 *In vivo* animal experiments

**FIGURE 9:**
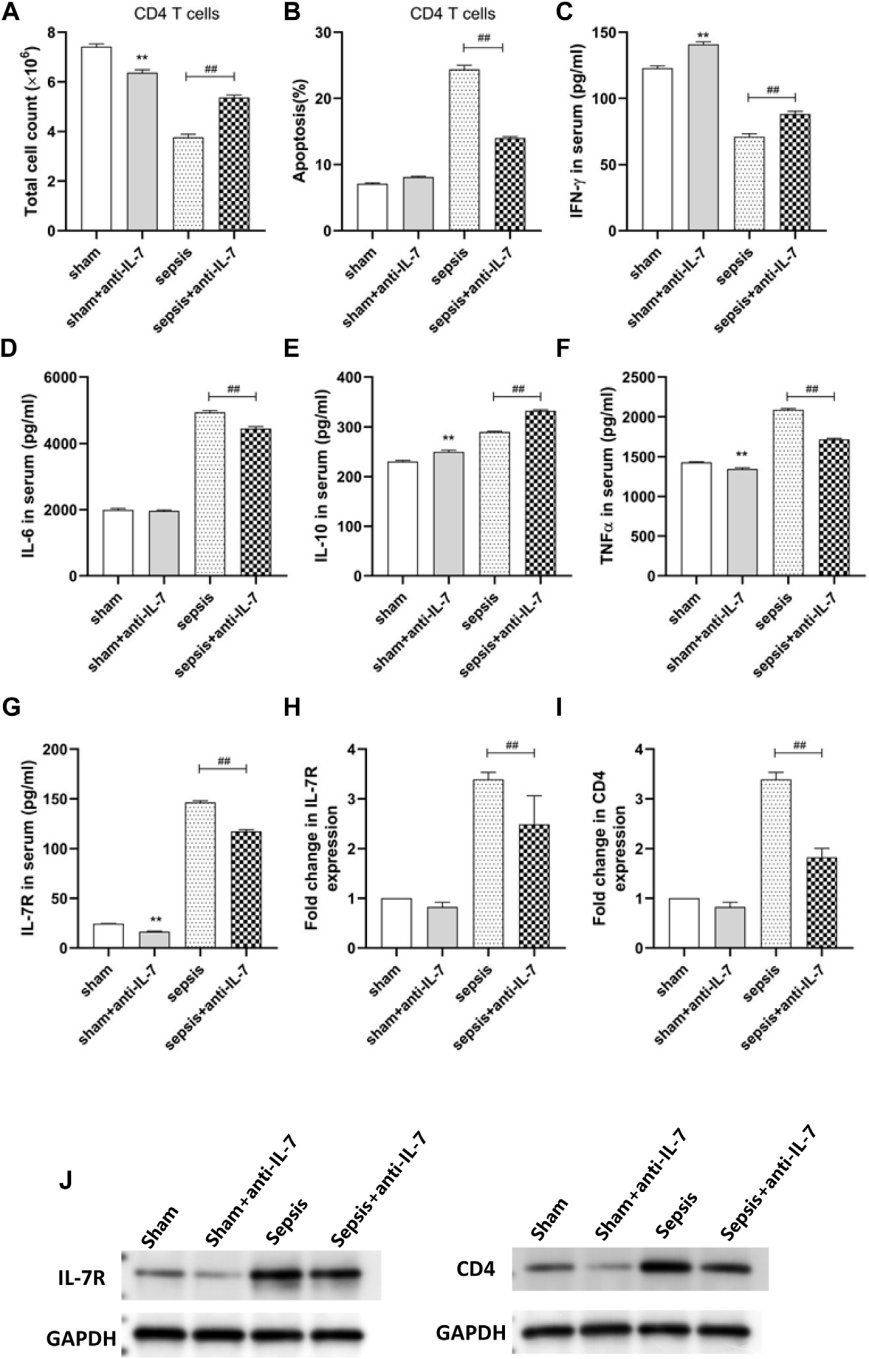
*In vivo* animal experiments. **(A)** The cell counts of CD4 T cells. **(B)** The apoptosis of CD4 T cells. **(C)** The concentration of IFN-γ. **(D)** The concentration of IL-6. **(E)** The concentration of IL -10. **(F)** The concentration of TNF-α. **(G)** The concentration of IL-7R. **(H)** The expression of IL-7R. **(I)** The expression of CD4. **(J)** Western blot assays.

We found that the cell counts of CD4 T cells were highest in sham group while lowest in sepsis group (Figure 9A). IL-7 antibody treatment could increase the counts of CD4 T cells (Figure 9A), which may be the cause of increased apoptosis (Figure 9B). The concentration of IFN-γ in peripheral blood were lowest in sepsis group and IL-7 antibody treatment could increase the concentration of IFN-γ (Figure 9C). The concentration of IL-6 in peripheral blood were highest in sepsis group and IL-7 antibody treatment could decrease the concentration of IL-6 (Figure 9D). IL-7 antibody treatment could increase the concentration of IL-10 (Figure 9E). The concentration of TNF-γ in peripheral blood were highest in sepsis group and IL-7 antibody treatment could decrease the concentration of TNF-α (Figure 9F). The concentration of IL-7R in peripheral blood were highest in sepsis group and IL-7 antibody treatment could decrease the concentration of IL-7R (Figure 9G). IL-7 antibody treatment could also decrease the expression of IL-7R and CD4 (Figures 9H–J).

## 4 Discussion

The imbalance of inflammatory response is the basis of sepsis, which runs through the whole process of sepsis. Pathogens that cause inflammation include bacteria, fungi, parasites, *etc.* The initial acute host response to an invasive pathogen usually manifests as macrophages engulfing the pathogen and producing a series of cytokines that trigger a cytokine storm and activate the innate immune 255 system (Hotchkiss et al., 2013; Rimmelé et al., 2016; Cai et al., 2020; Nedeva, 2021). Studies have shown that preventing lymphocyte apoptosis can improve the survival rate of patients with sepsis (Zhu et al., 2012). The harm of apoptosis is not only related to the serious loss of immune cells, but also related to the effect of uptake of apoptotic cells on surviving immune cells (Zhu et al., 2012). Our data showed that the cell counts of CD4 T cells were highest in sham group while lowest in sepsis group (Figure 9A). IL-7 antibody treatment could increase the counts of CD4 T cells (Figure 9A), which may be the cause of increased apoptosis (Figure 9B). However, in sepsis, apoptosis of neutrophils is delayed in contrast to that of other lymphocytes, where apoptosis is accelerated (Hazeldine et al., 2017). Dendritic cell showed obvious apoptosis in sepsis. The number of circulating and spleen dendritic cell and the percentage of spleen area occupied by dendritic cell were significantly reduced in *postmortem* reports of patients with sepsis (Hotchkiss et al., 2002). However, the loss of dendritic cell was more serious in the patients with sepsis death than in the survivors, and in the patients with sepsis in the later stage, not only the number of dendritic cell decreased, but also the ability of surviving dendritic cell antigen presentation, the expression level of HLA-DR decreased, and the secretion of IL-10 increased. Our research showed that IL-7 antibody treatment could increase the concentration of IL-10 in peripheral blood. Uptake of apoptotic cells by monocytes, macrophages and DC promotes the production of interleukin-10 (IL-10) by inducing the proliferation of non-functional cells, leading to immune tolerance (Luan et al., 2014). Our data showed that IL-7 antibody treatment could increase the concentration of IL-10 (Figure 9E). Therefore, immune cells play a key role in immune response and maintenance of immune balance during sepsis. Understanding the changes and mechanisms of different immune cell populations in sepsis may bring new ideas for the treatment of sepsis. For instance, myeloid-derived suppressor cellsn (MDSC) are reported to be elevated in patients with sepsis (Ost et al., 2016; Mathias et al., 2017). MDSC is produced in response to various inflammatory and infectious stimuli. The most important feature of MDSC in sepsis is its immunosuppressive function. MDSC reduces innate and acquired immune responses by producing immunosuppressive substances and inhibiting the proliferation and activation of T cells (Mathias et al., 2017). Currently, the biomarkers of various infectious diseases are very limited, but the emerging single cell sequencing (scRNA-seq) technology greatly facilitates the identification of disease-related biomarkers. For example, MX2 of naive B cells and CD163 and IFIT1 of CD14^+^CD16^+^ monocytes in peripheral blood of dengue patients were significantly upregulated before onset of dengue fever, indicating that these genes with significantly altered expression levels have the potential to be biomarkers for predicting dengue disease (Zanini et al., 2018). Cai et al. found that CD3^−^CD7+GZMB + NK cell subpopulation can be used as a new biomarker to identify patients with active tuberculosis and monitor treatment response (Cai et al., 2020). Hence, in current infectious disease research, single-cell transcriptome sequencing may be used to monitor the gene expression patterns associated with specific infections, which can reveal more candidate biomarkers for diagnosis and prognosis of infectious diseases (including sepsis). In this study, using single-cell transcriptome sequencing, we carried out hub genes screening based on PPI network of immune-related genes, and seven hub genes (including CD28, CD3D, CD2, CD4, IL7R, LCK, and CD3E) were screened utilizing three algorithms (Maximal Clique Centrality, Maximum neighborhood component, and Density of Maximum Neighborhood Component). We also found that the expression level of CD3D (Figure 6A), CD3E (Figure 6B), CD4 (Figure 6C), CD28 (Figure 6D), IL7R (Figure 6E), and LCK (Figure 6F) was statistically different between sepsis and normal groups, indicating their potential role in the development of sepsis.

Sepsis has always been an important clinical problem. Early identification and timely treatment are of great significance for sepsis. Currently, there are many sepsis related survival prediction models. For example, an overexpression of PD⁃1 based on a single immune checkpoint in regulatory T cells predicts the prognosis of patients with sepsis, and its ability to validate 28-day mortality in patients with sepsis (AUC = 0.792) (Jiang et al., 2020). However, the pathogenesis of sepsis is complicated, especially the immune mechanism. In our study, we observed the significant difference of B cells naïve, plasma cells, T cells CD8, T cells CD4 naïve, T cells CD4 memory resting, T cells CD4 memory activated, T cells gamma delta, NK cells resting, Monocytes, Macrophages M0, Eosinophils, Neutrophils cells between sepsis samples and control samples (Figure 7B). The lower expression of the hub genes (CD3D, CD3E, CD4, CD28, IL7R and LCK) was observed in sepsis samples, which was consistent in all the six hub genes (Figure 6). This study still has certain limitations. Bioinformatics methods were used to explore the hub gene and immune cell infiltration in sepsis, but external cohort verification was not conducted, so the accuracy of external cohort verification conclusions could be increased in the future. In addition, further experiments are needed to verify the roles and associations of sepsis related genes, signaling pathways and immune cells discussed in this paper.

## Data Availability

The original contributions presented in the study are included in the article/Supplementary Material, further inquiries can be directed to the corresponding authors.
