# Supplementary material for: Single-cell transcriptomics reveals immune infiltrate in sepsis
Source: Front Pharmacol. 2023 Apr 11;14:1133145. doi: 10.3389/fphar.2023.1133145 (PMC10126435; doi:10.3389/fphar.2023.1133145)
Supplement: Supplementary file 1 [file DataSheet1.ZIP › CD28/correlation scatter plot between expression of CD28 and Dendritic cells activated.pdf]

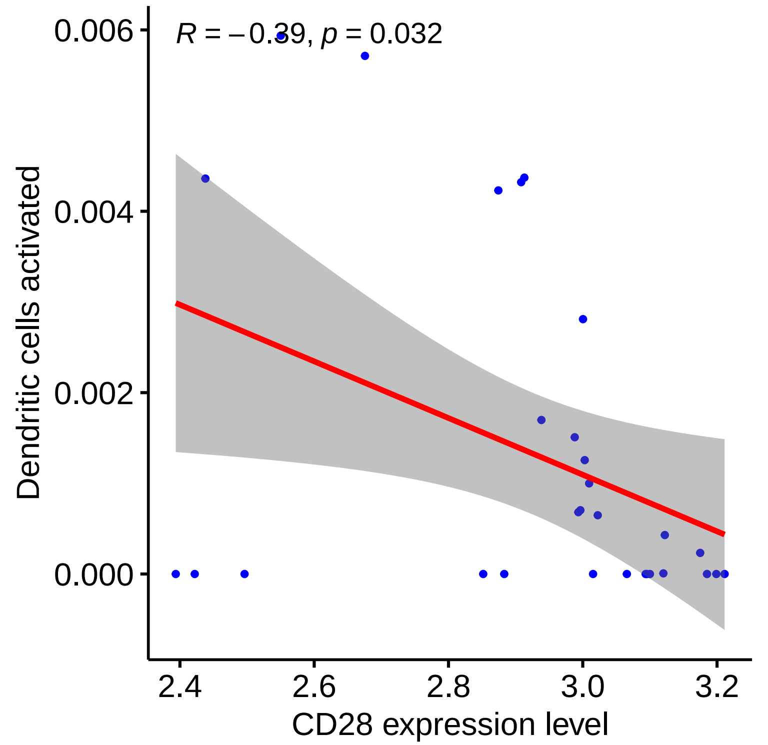
R = −0.39, p = 0.032
Dendritic cells activated
0.006
0.004
0.002
0.000
2.4
2.6
2.8
3.0
3.2
CD28 expression level
