# Supplementary figures and images for: Single-cell transcriptomics reveals immune infiltrate in sepsis
Source: Front Pharmacol. 2023 Apr 11;14:1133145. doi: 10.3389/fphar.2023.1133145 (PMC10126435; doi:10.3389/fphar.2023.1133145)

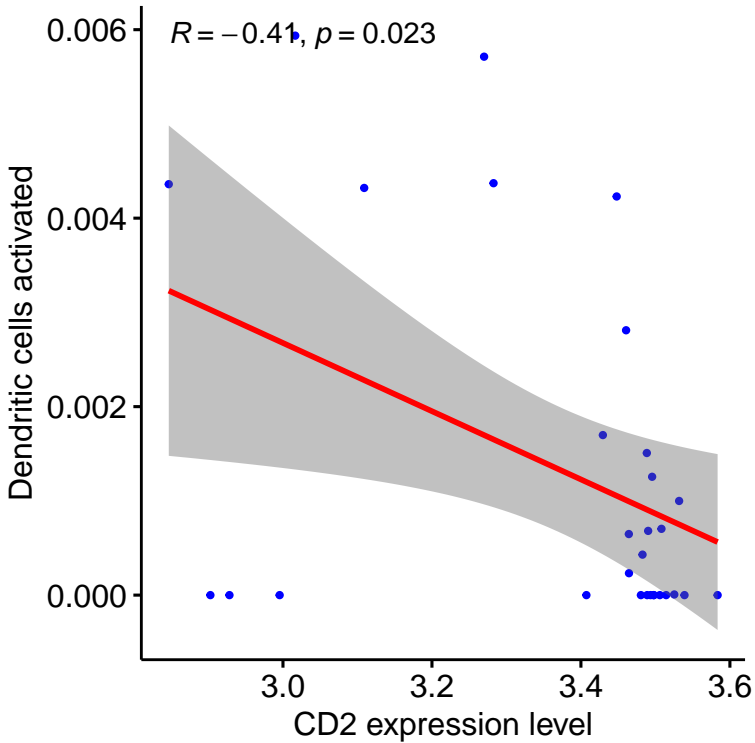

Supplement: Supplementary file 1 [file DataSheet1.ZIP › CD2/correlation scatter plot between expression of CD2 and Dendritic cells activated.pdf]

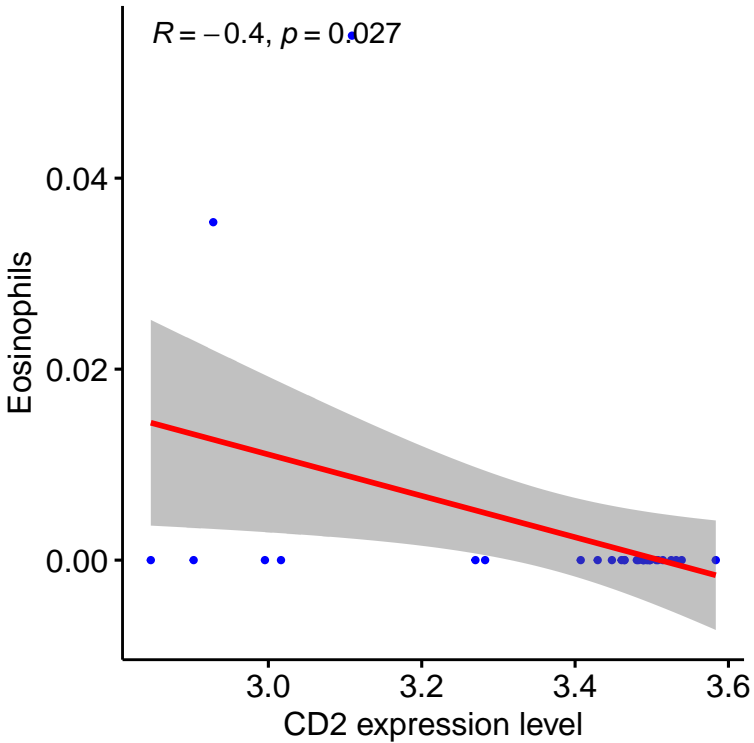

Supplement: Supplementary file 1 [file DataSheet1.ZIP › CD2/correlation scatter plot between expression of CD2 and Eosinophils.pdf]

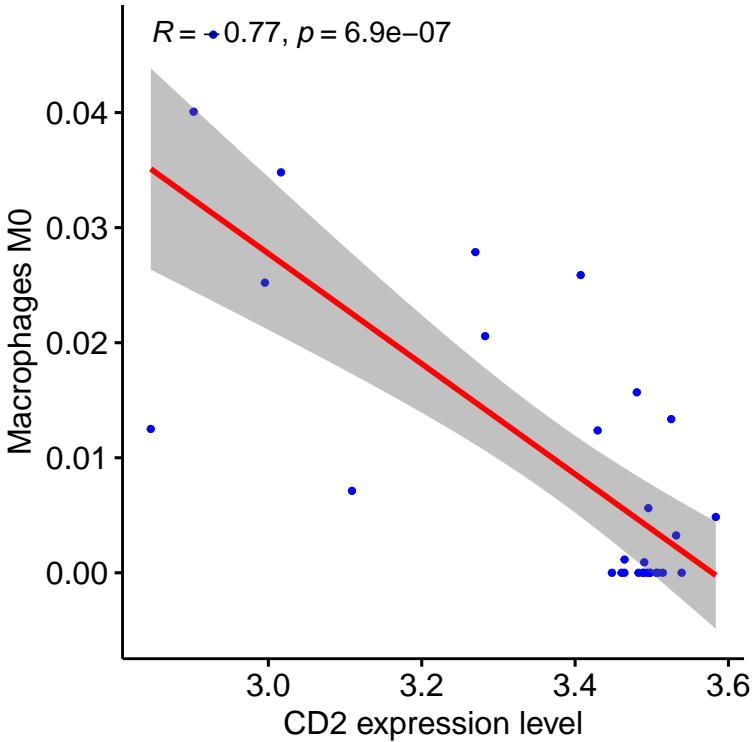

Supplement: Supplementary file 1 [file DataSheet1.ZIP › CD2/correlation scatter plot between expression of CD2 and Macrophages M0.pdf]

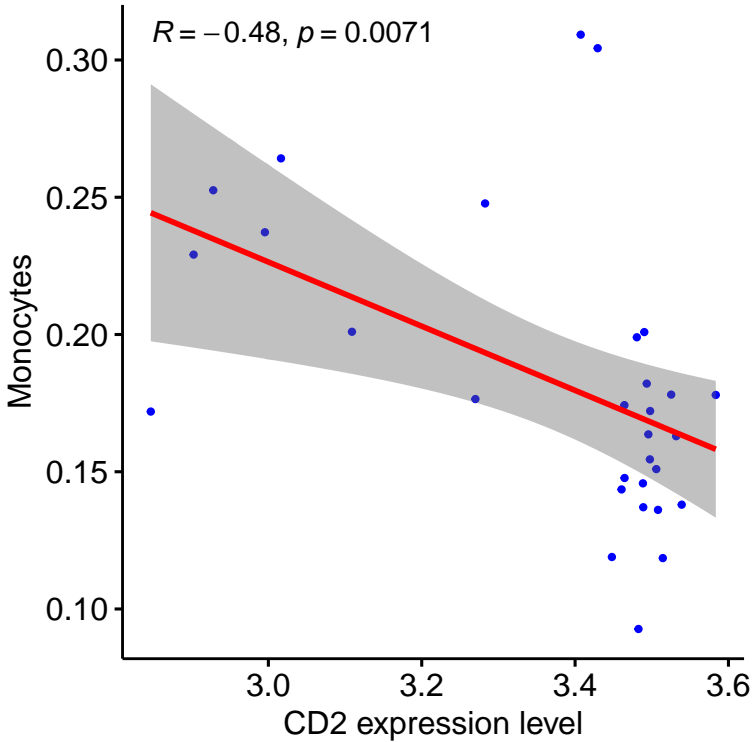

Supplement: Supplementary file 1 [file DataSheet1.ZIP › CD2/correlation scatter plot between expression of CD2 and Monocytes.pdf]

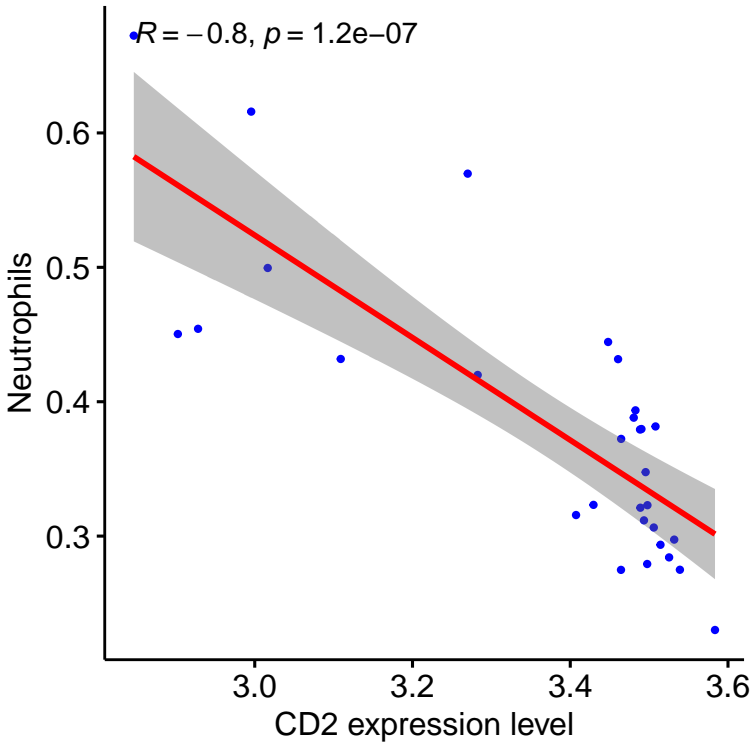

Supplement: Supplementary file 1 [file DataSheet1.ZIP › CD2/correlation scatter plot between expression of CD2 and Neutrophils.pdf]

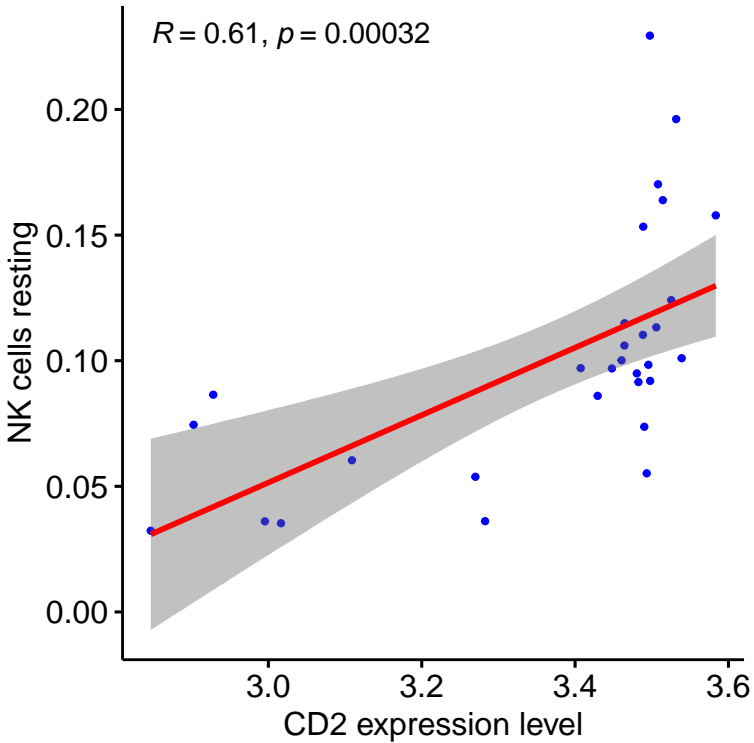

Supplement: Supplementary file 1 [file DataSheet1.ZIP › CD2/correlation scatter plot between expression of CD2 and NK cells resting.pdf]

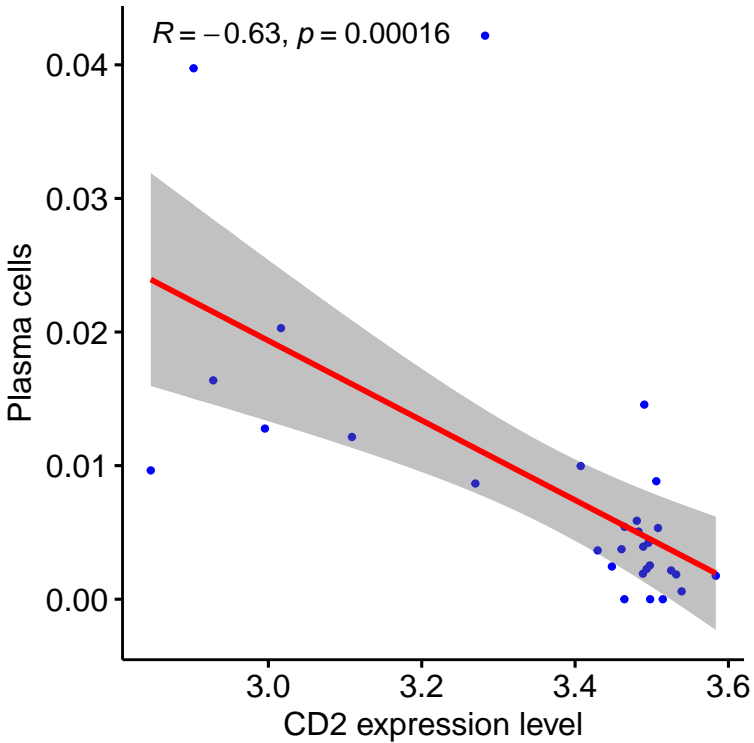

Supplement: Supplementary file 1 [file DataSheet1.ZIP › CD2/correlation scatter plot between expression of CD2 and Plasma cells.pdf]

T cells CD4 memory activated

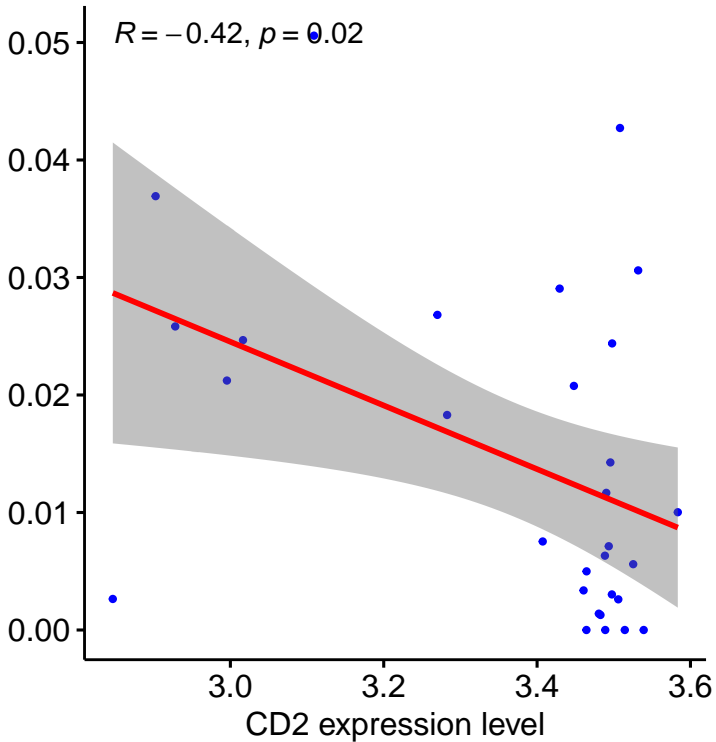

Supplement: Supplementary file 1 [file DataSheet1.ZIP › CD2/correlation scatter plot between expression of CD2 and T cells CD4 memory activated.pdf]

T cells CD4 memory resting

$R = 0.64$ ,  $p = 0.00013$

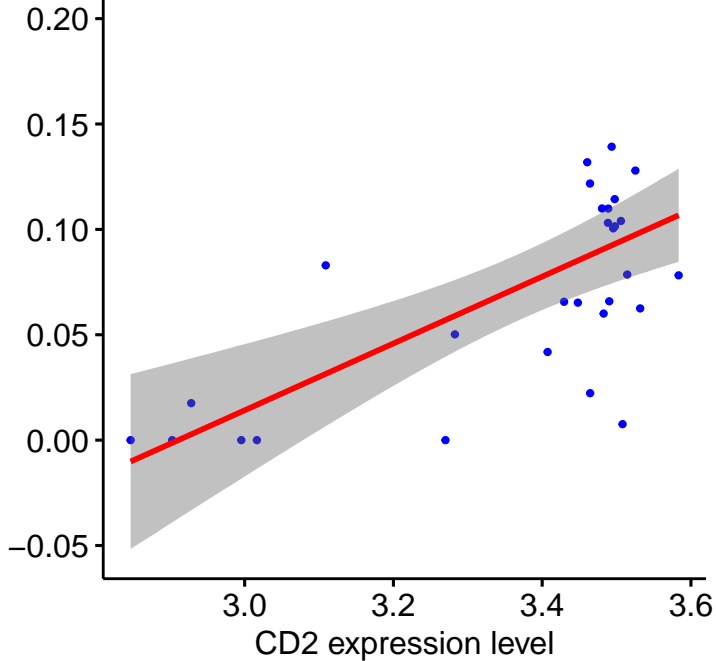

Supplement: Supplementary file 1 [file DataSheet1.ZIP › CD2/correlation scatter plot between expression of CD2 and T cells CD4 memory resting.pdf]

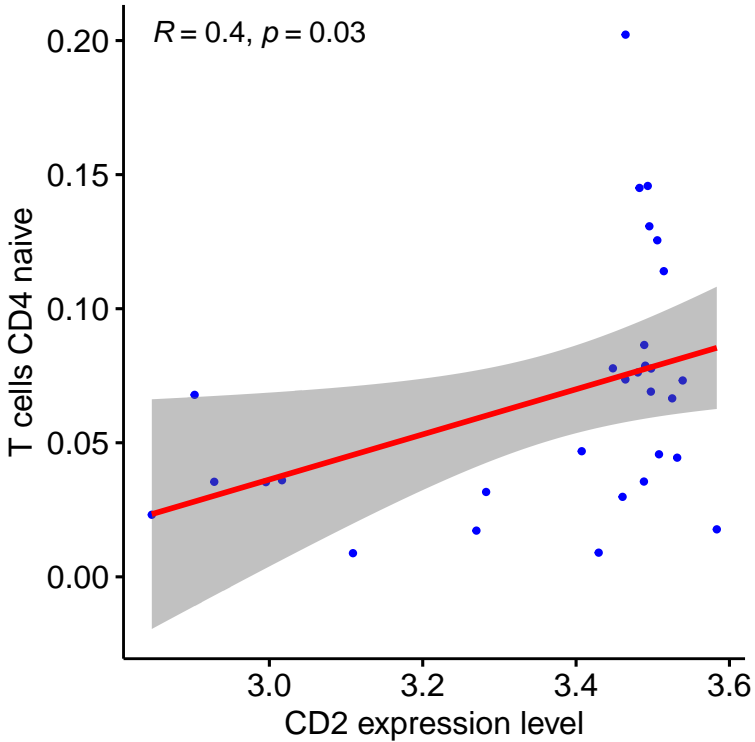

Supplement: Supplementary file 1 [file DataSheet1.ZIP › CD2/correlation scatter plot between expression of CD2 and T cells CD4 naive.pdf]

$R = 0.8, p = 8.2e-08$

T cells CD8

0.2

0.1

0.0

3.0

3.2

3.4

3.6

CD2 expression level

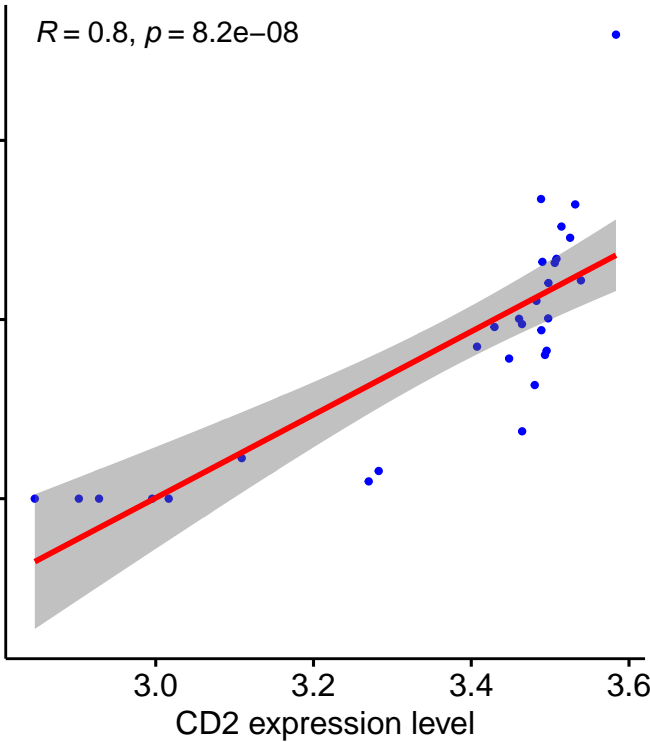

Supplement: Supplementary file 1 [file DataSheet1.ZIP › CD2/correlation scatter plot between expression of CD2 and T cells CD8.pdf]

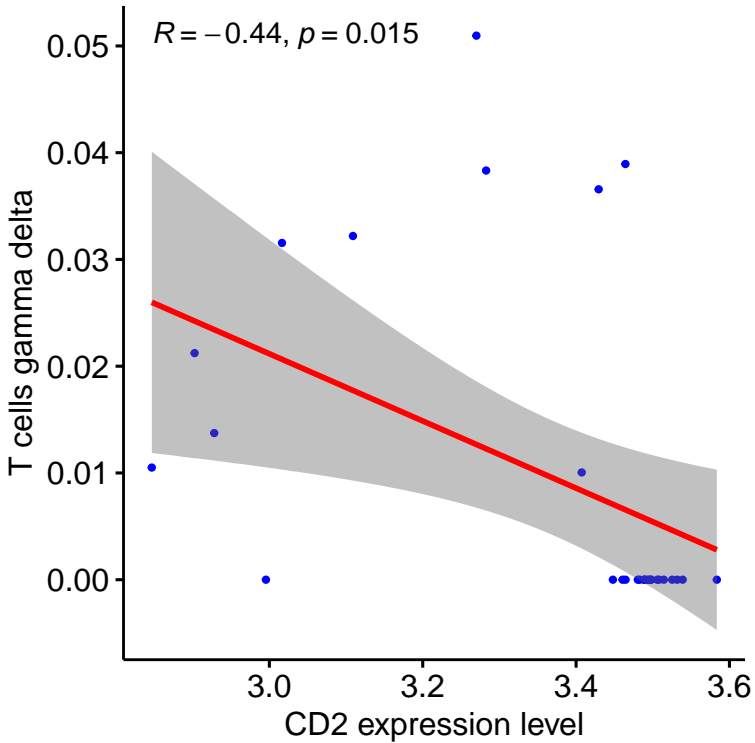

Supplement: Supplementary file 1 [file DataSheet1.ZIP › CD2/correlation scatter plot between expression of CD2 and T cells gamma delta.pdf]

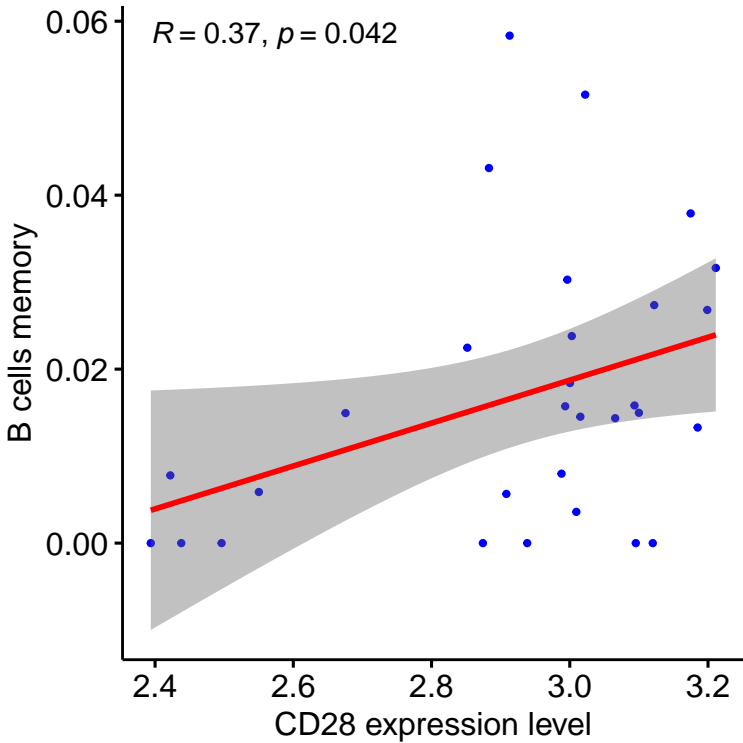

Supplement: Supplementary file 1 [file DataSheet1.ZIP › CD28/correlation scatter plot between expression of CD28 and B cells memory.pdf]

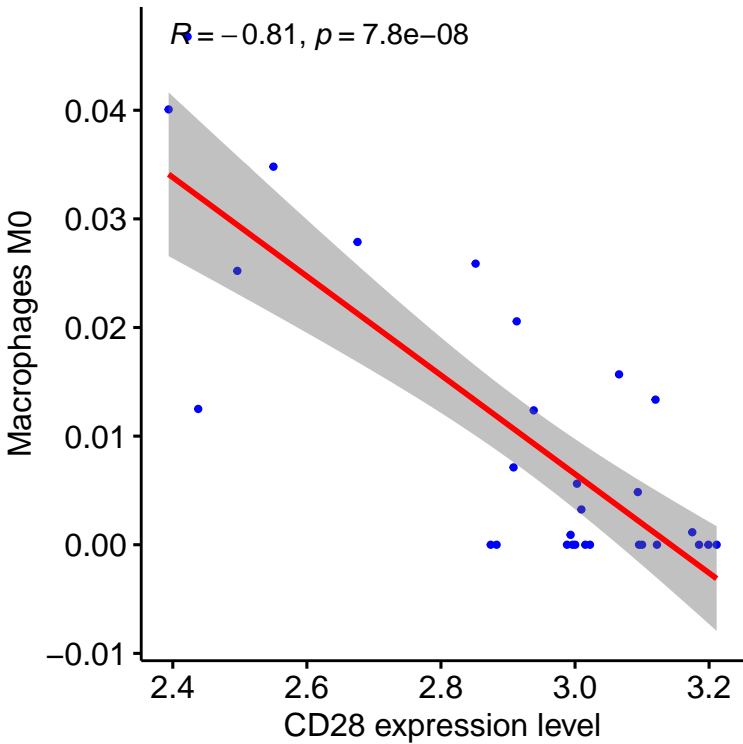

Supplement: Supplementary file 1 [file DataSheet1.ZIP › CD28/correlation scatter plot between expression of CD28 and Macrophages M0.pdf]

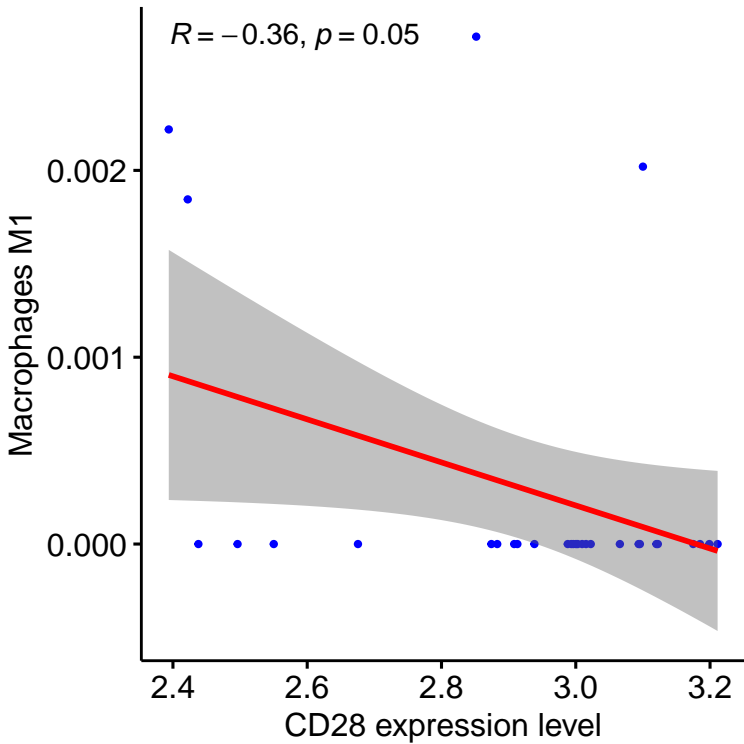

Supplement: Supplementary file 1 [file DataSheet1.ZIP › CD28/correlation scatter plot between expression of CD28 and Macrophages M1.pdf]

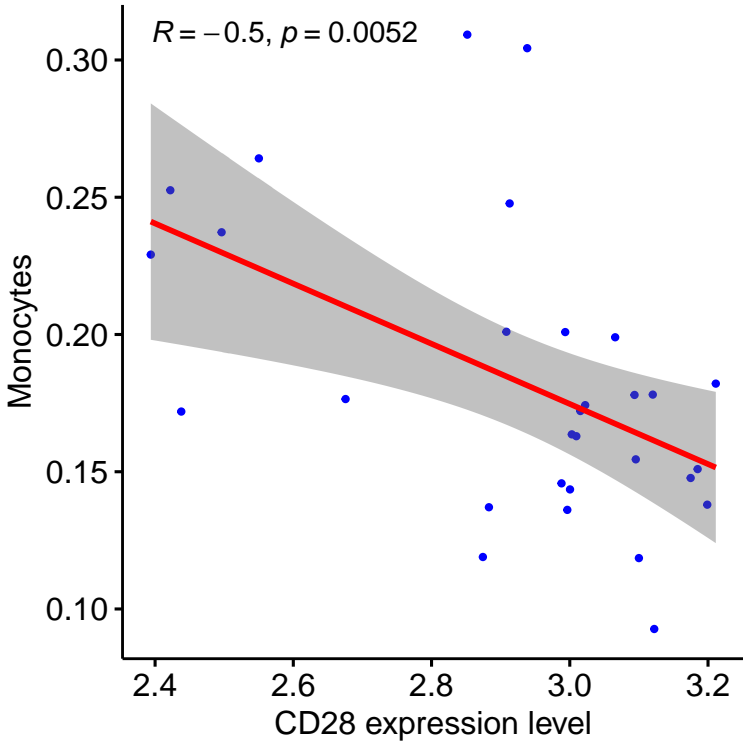

Supplement: Supplementary file 1 [file DataSheet1.ZIP › CD28/correlation scatter plot between expression of CD28 and Monocytes.pdf]

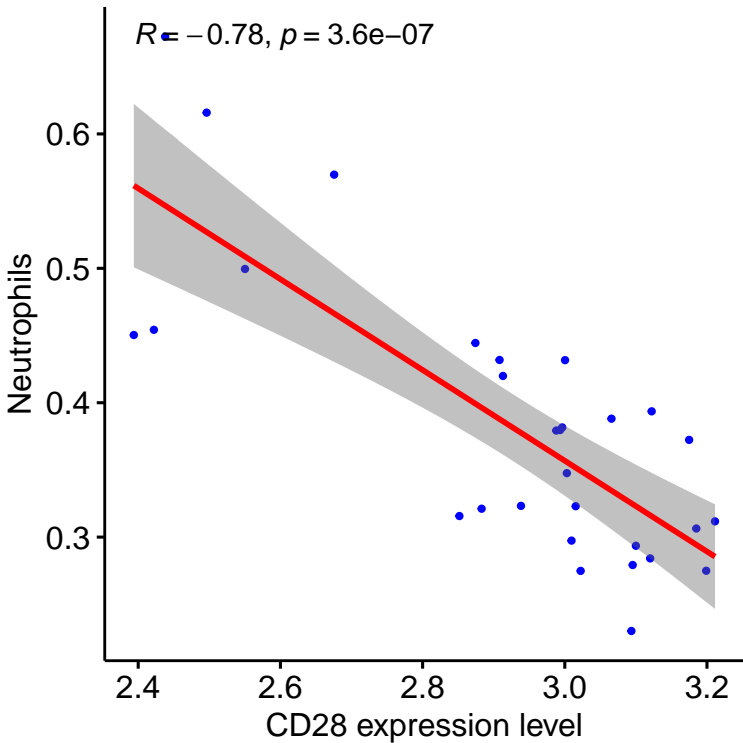

Supplement: Supplementary file 1 [file DataSheet1.ZIP › CD28/correlation scatter plot between expression of CD28 and Neutrophils.pdf]

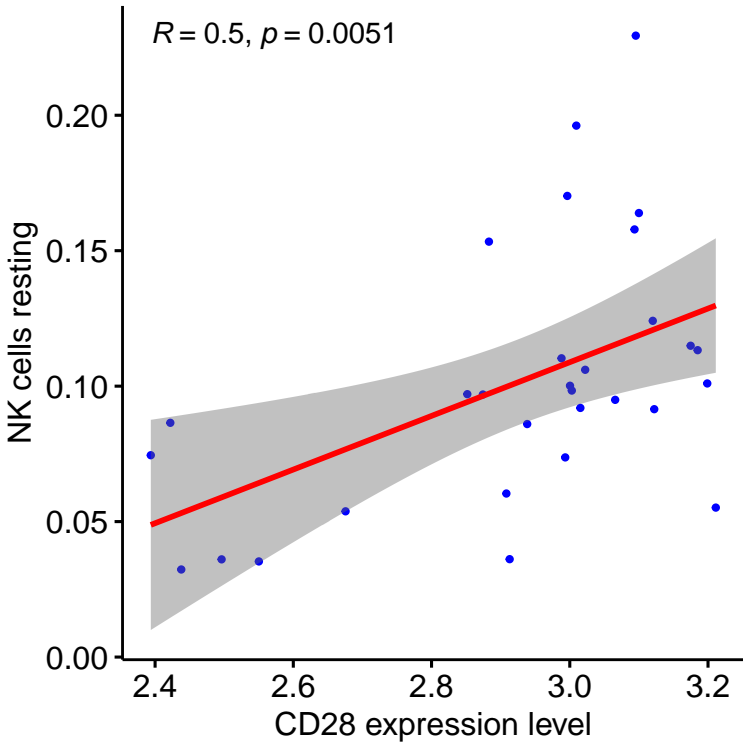

Supplement: Supplementary file 1 [file DataSheet1.ZIP › CD28/correlation scatter plot between expression of CD28 and NK cells resting.pdf]

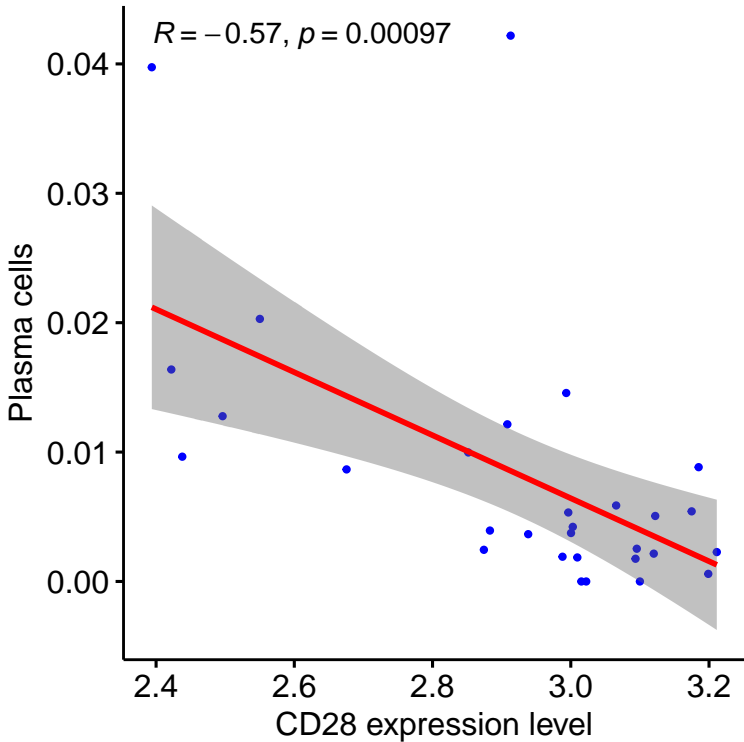

Supplement: Supplementary file 1 [file DataSheet1.ZIP › CD28/correlation scatter plot between expression of CD28 and Plasma cells.pdf]

T cells CD4 memory activated

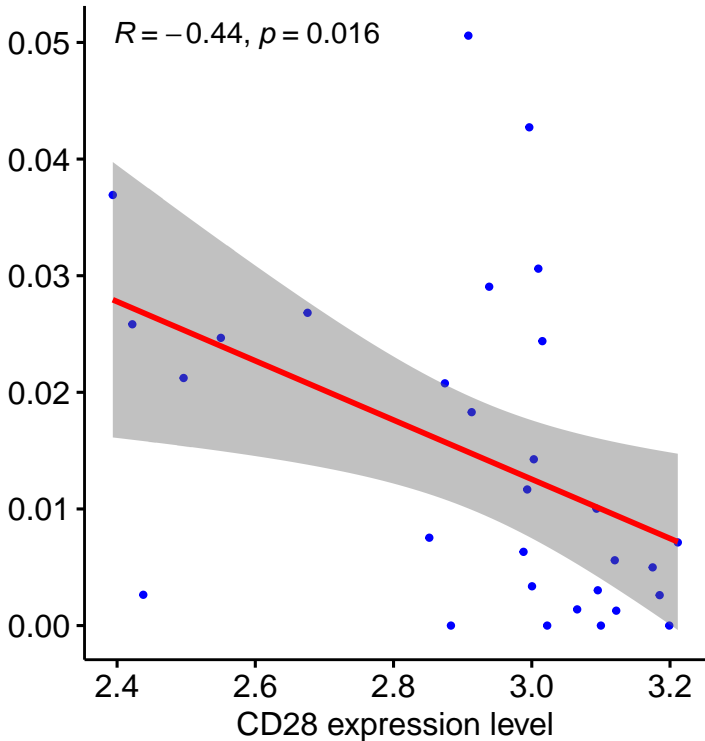

Supplement: Supplementary file 1 [file DataSheet1.ZIP › CD28/correlation scatter plot between expression of CD28 and T cells CD4 memory activated.pdf]

T cells CD4 memory resting

$R = 0.71$ ,  $p = 9.7\text{e-}06$

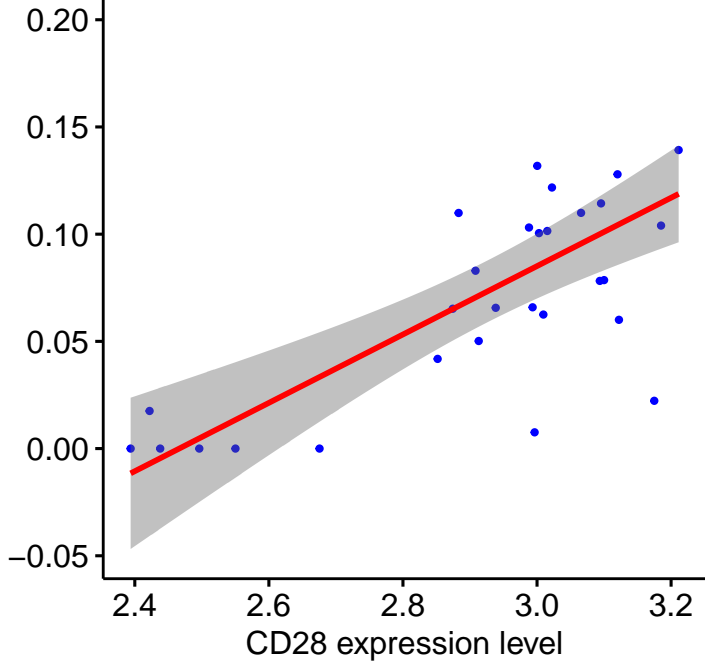

Supplement: Supplementary file 1 [file DataSheet1.ZIP › CD28/correlation scatter plot between expression of CD28 and T cells CD4 memory resting.pdf]

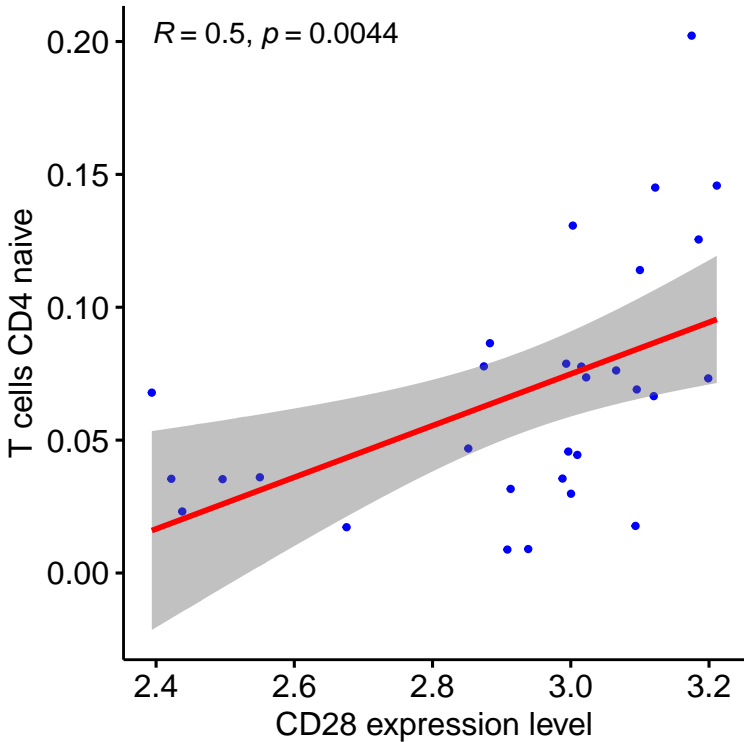

Supplement: Supplementary file 1 [file DataSheet1.ZIP › CD28/correlation scatter plot between expression of CD28 and T cells CD4 naive.pdf]

$R = 0.7, p = 1.8\text{e-}05$

T cells CD8

0.2

0.1

0.0

2.4

2.6

2.8

3.0

3.2

CD28 expression level

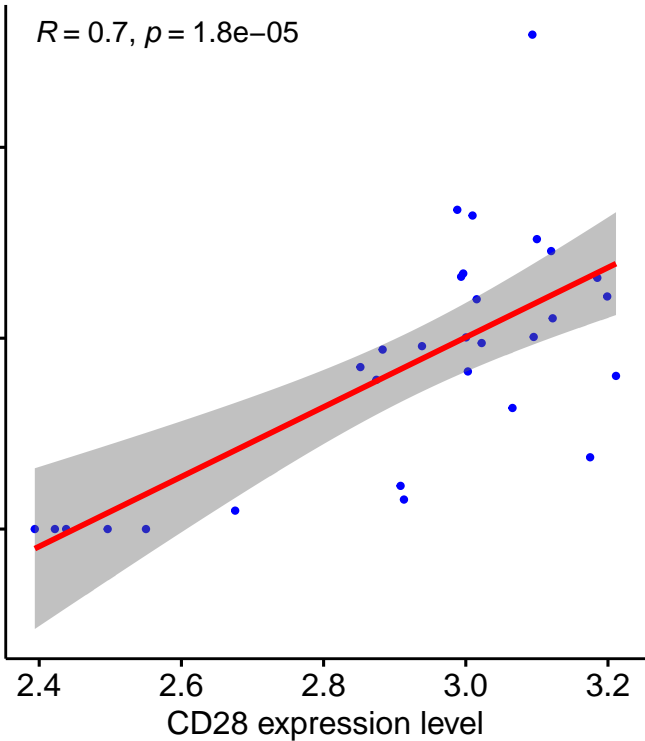

Supplement: Supplementary file 1 [file DataSheet1.ZIP › CD28/correlation scatter plot between expression of CD28 and T cells CD8.pdf]

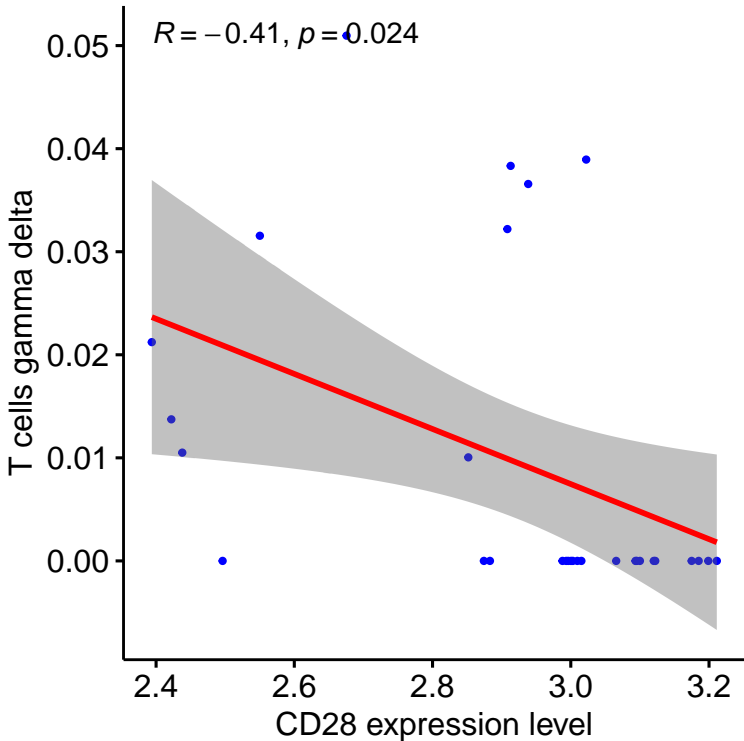

Supplement: Supplementary file 1 [file DataSheet1.ZIP › CD28/correlation scatter plot between expression of CD28 and T cells gamma delta.pdf]

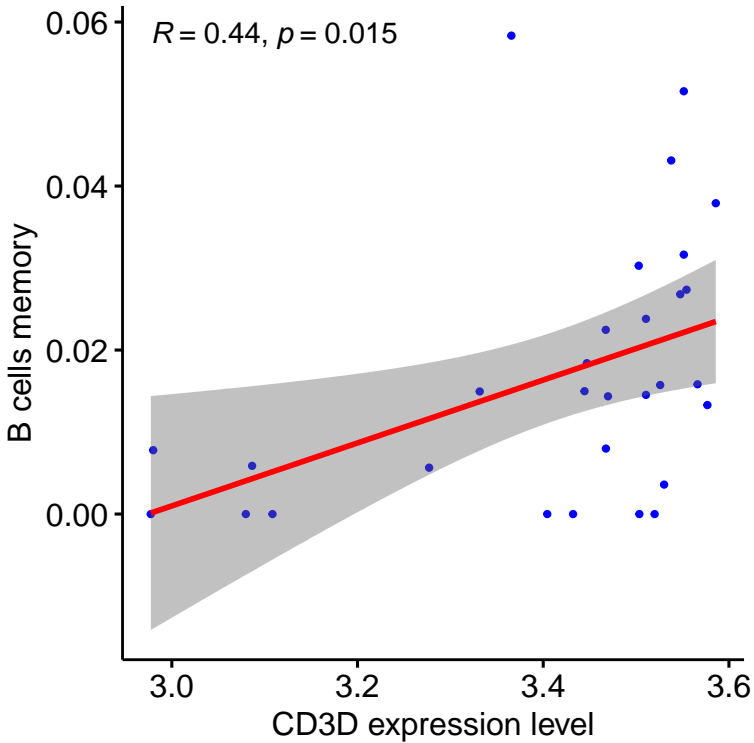

Supplement: Supplementary file 1 [file DataSheet1.ZIP › CD3D/correlation scatter plot between expression of CD3D and B cells memory.pdf]

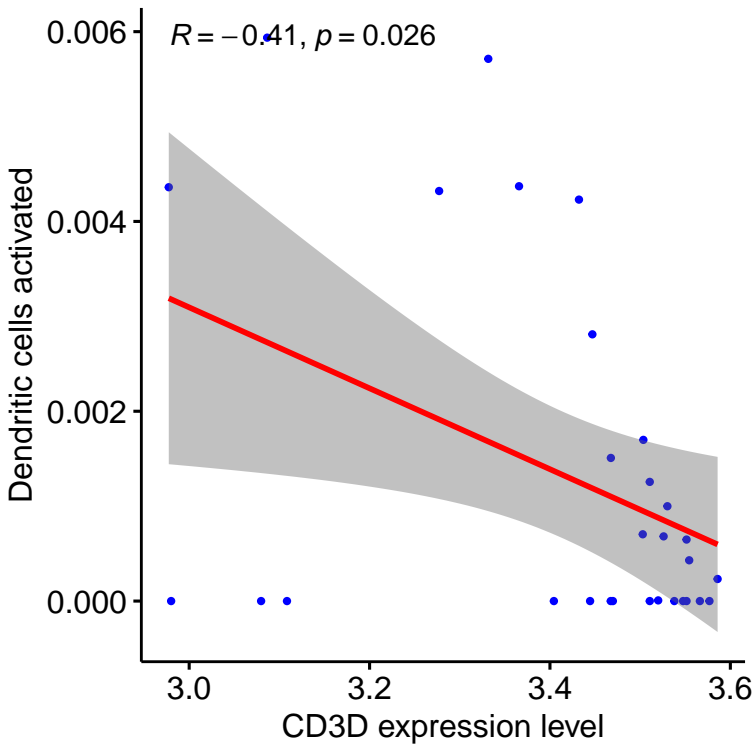

Supplement: Supplementary file 1 [file DataSheet1.ZIP › CD3D/correlation scatter plot between expression of CD3D and Dendritic cells activated.pdf]

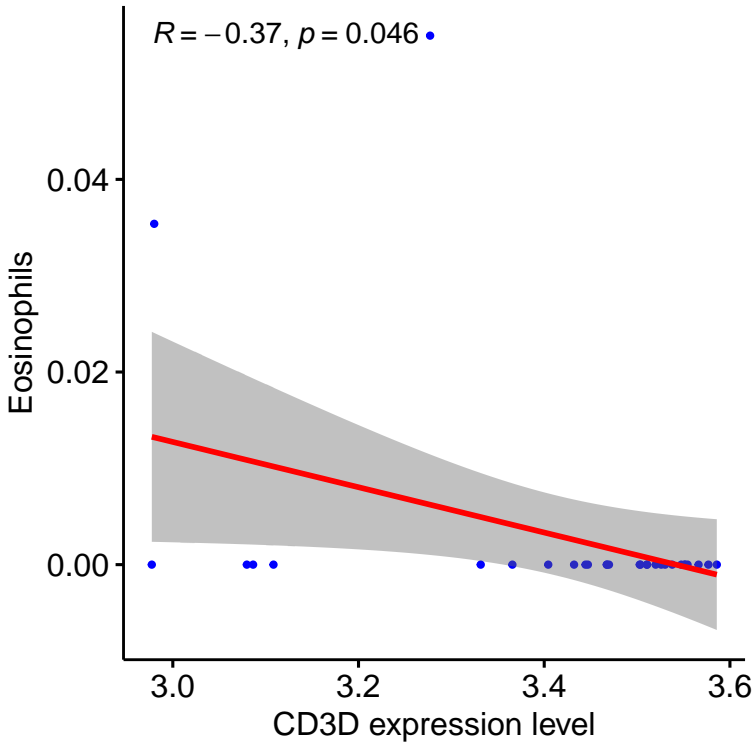

Supplement: Supplementary file 1 [file DataSheet1.ZIP › CD3D/correlation scatter plot between expression of CD3D and Eosinophils.pdf]

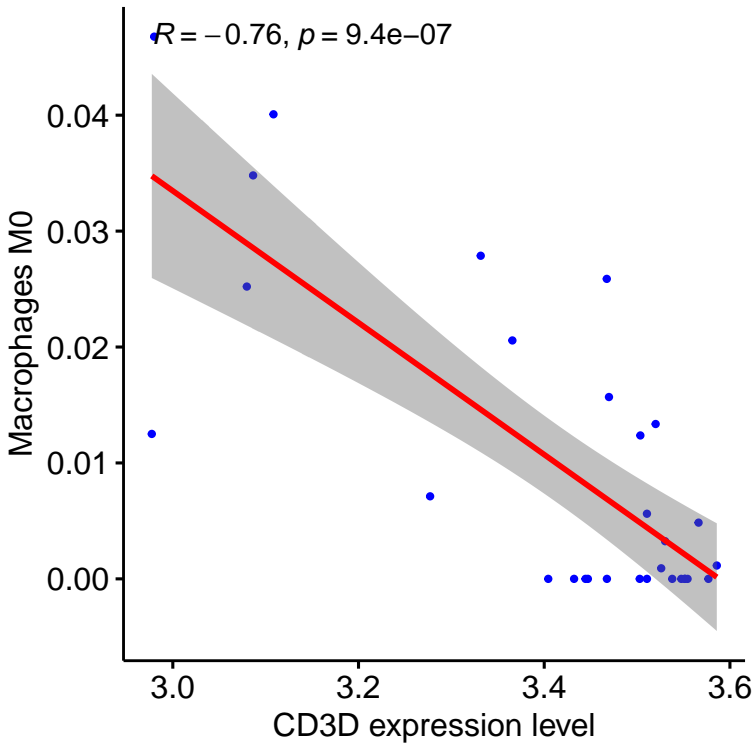

Supplement: Supplementary file 1 [file DataSheet1.ZIP › CD3D/correlation scatter plot between expression of CD3D and Macrophages M0.pdf]

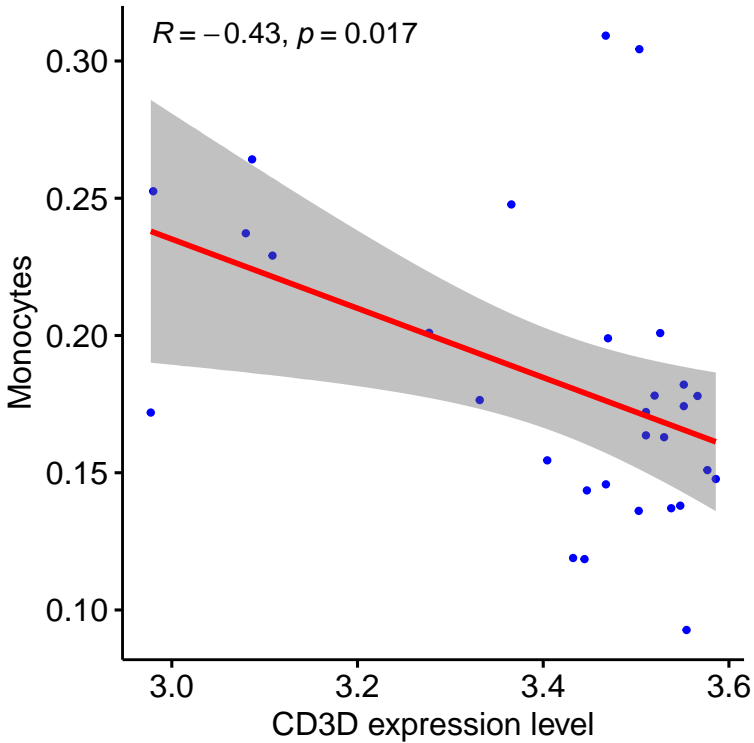

Supplement: Supplementary file 1 [file DataSheet1.ZIP › CD3D/correlation scatter plot between expression of CD3D and Monocytes.pdf]

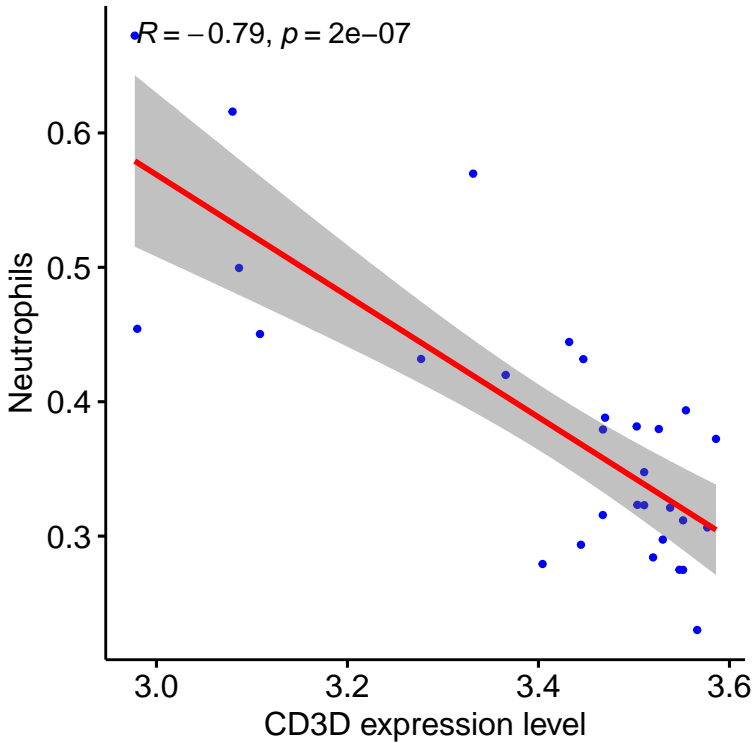

Supplement: Supplementary file 1 [file DataSheet1.ZIP › CD3D/correlation scatter plot between expression of CD3D and Neutrophils.pdf]

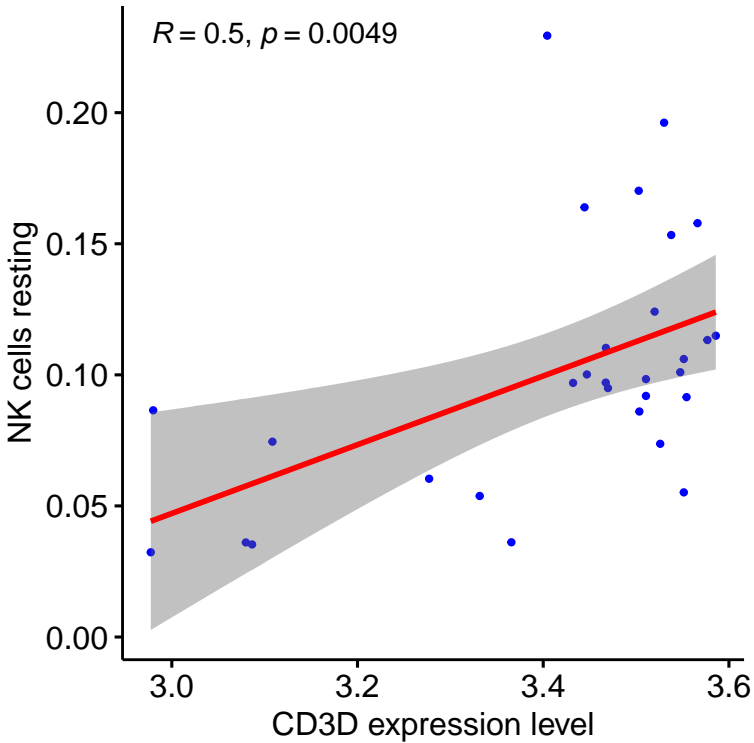

Supplement: Supplementary file 1 [file DataSheet1.ZIP › CD3D/correlation scatter plot between expression of CD3D and NK cells resting.pdf]

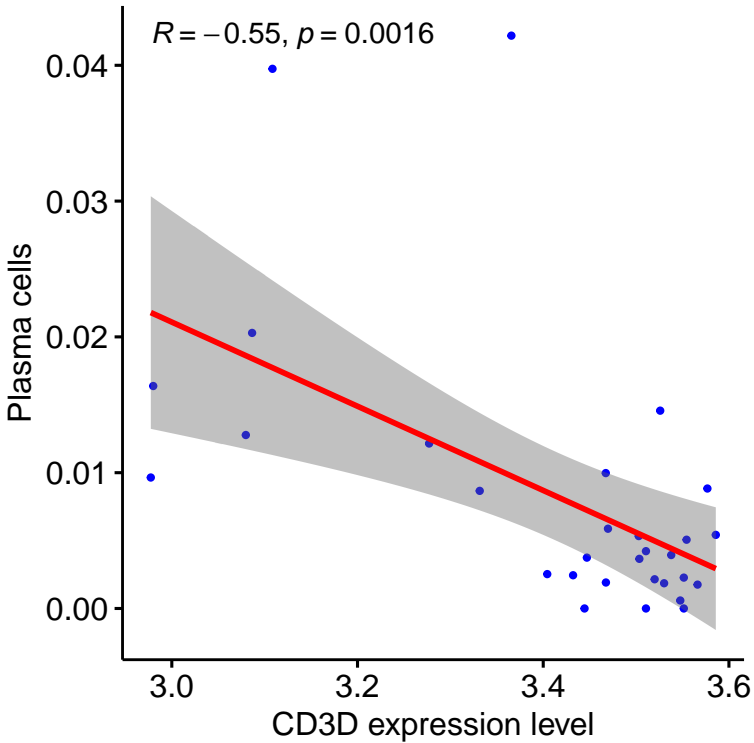

Supplement: Supplementary file 1 [file DataSheet1.ZIP › CD3D/correlation scatter plot between expression of CD3D and Plasma cells.pdf]

T cells CD4 memory activated

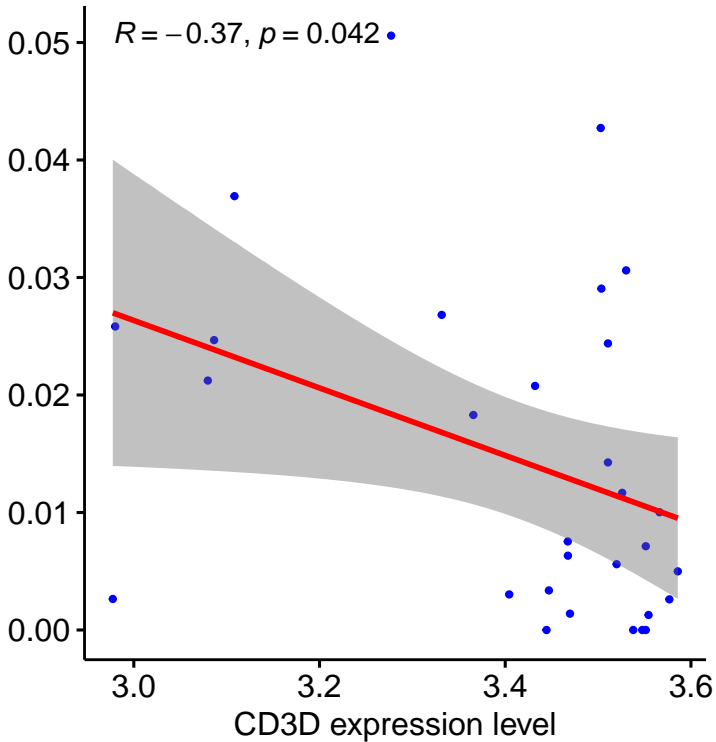

Supplement: Supplementary file 1 [file DataSheet1.ZIP › CD3D/correlation scatter plot between expression of CD3D and T cells CD4 memory activated.pdf]

T cells CD4 memory resting

$R = 0.61$ ,  $p = 0.00033$

0.20  
0.15  
0.10  
0.05  
0.00  
-0.05

3.0

3.2

3.4

3.6

CD3D expression level

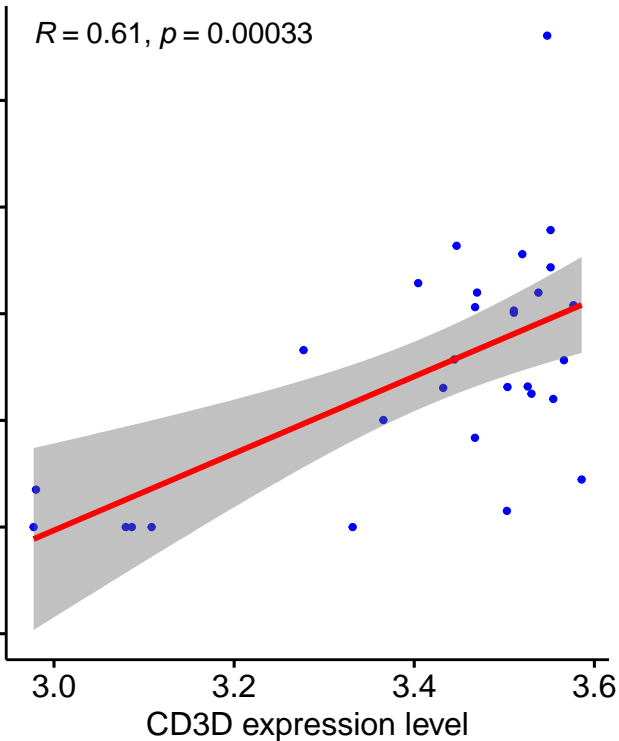

Supplement: Supplementary file 1 [file DataSheet1.ZIP › CD3D/correlation scatter plot between expression of CD3D and T cells CD4 memory resting.pdf]

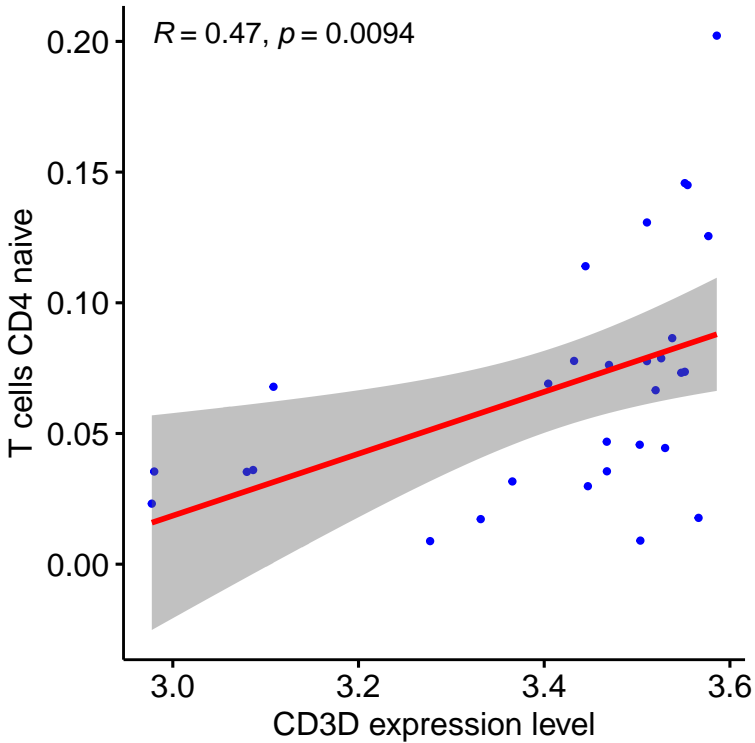

Supplement: Supplementary file 1 [file DataSheet1.ZIP › CD3D/correlation scatter plot between expression of CD3D and T cells CD4 naive.pdf]

$R = 0.73$ ,  $p = 4e-06$

T cells CD8

0.2

0.1

0.0

3.0

3.2

3.4

3.6

CD3D expression level

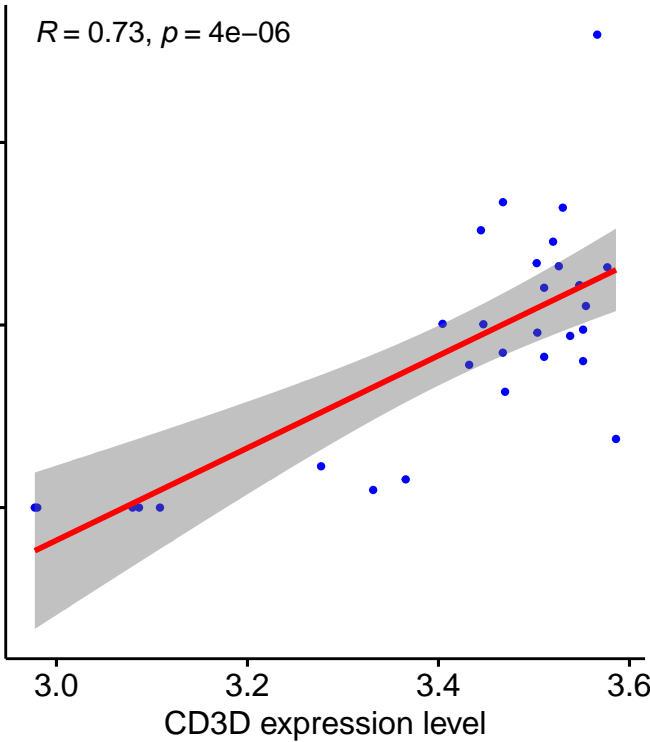

Supplement: Supplementary file 1 [file DataSheet1.ZIP › CD3D/correlation scatter plot between expression of CD3D and T cells CD8.pdf]

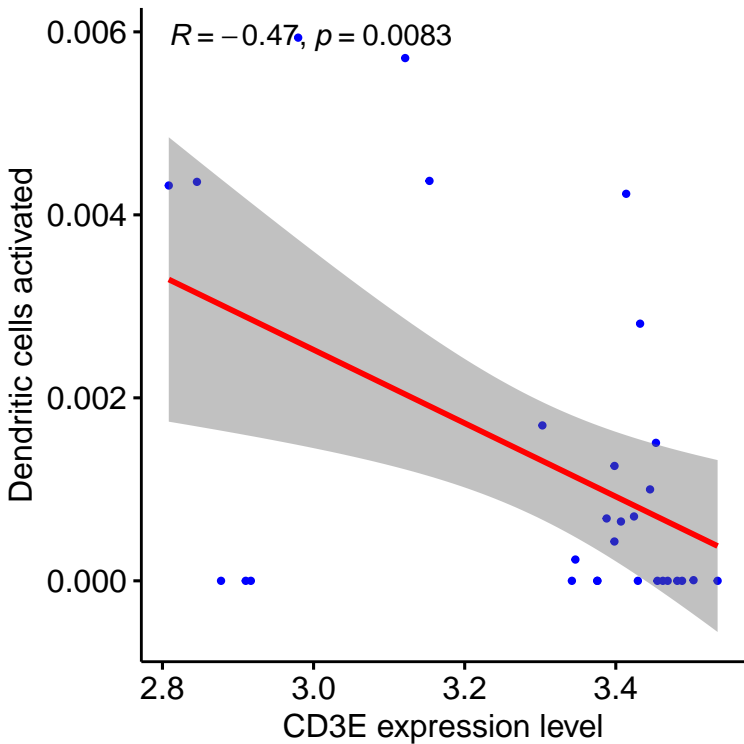

Supplement: Supplementary file 1 [file DataSheet1.ZIP › CD3E/correlation scatter plot between expression of CD3E and Dendritic cells activated.pdf]

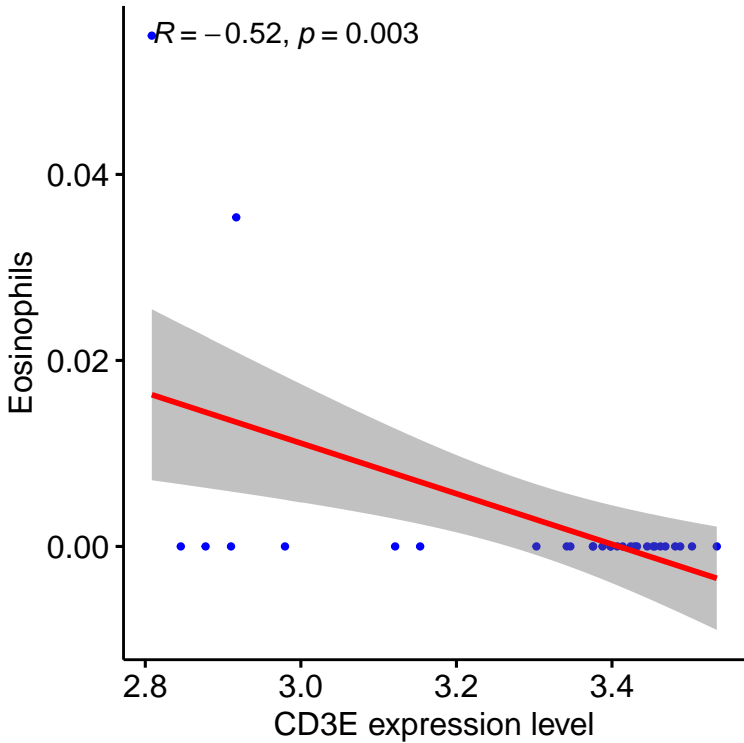

Supplement: Supplementary file 1 [file DataSheet1.ZIP › CD3E/correlation scatter plot between expression of CD3E and Eosinophils.pdf]

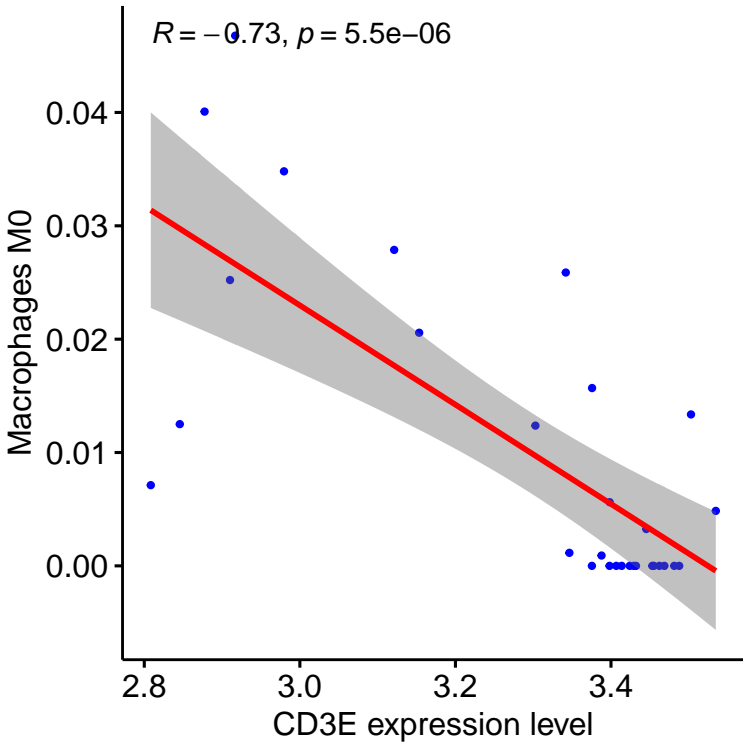

Supplement: Supplementary file 1 [file DataSheet1.ZIP › CD3E/correlation scatter plot between expression of CD3E and Macrophages M0.pdf]

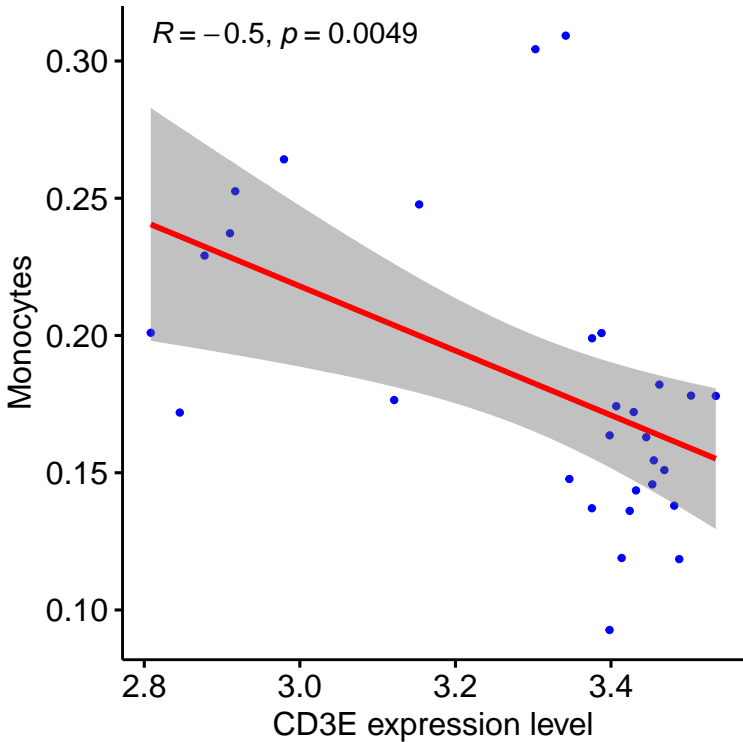

Supplement: Supplementary file 1 [file DataSheet1.ZIP › CD3E/correlation scatter plot between expression of CD3E and Monocytes.pdf]

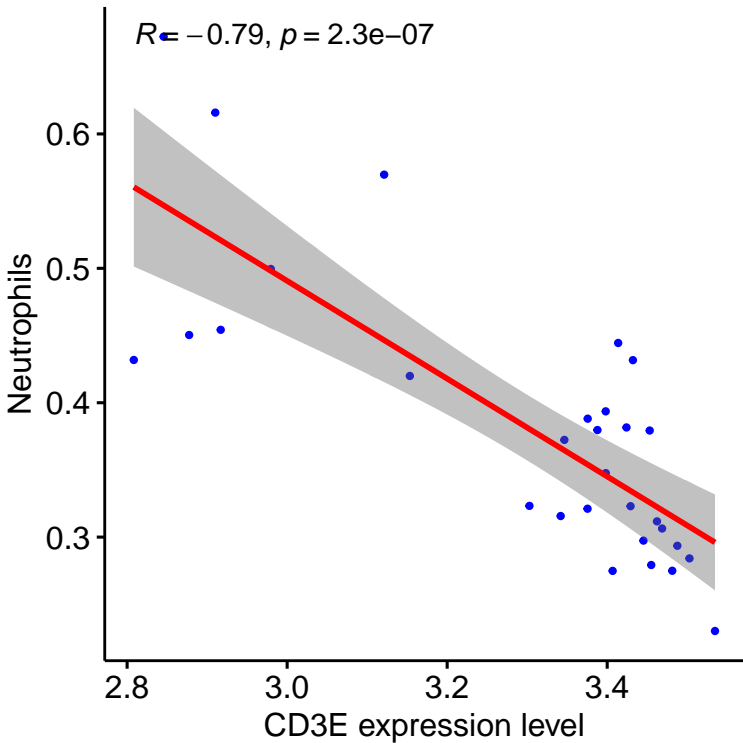

Supplement: Supplementary file 1 [file DataSheet1.ZIP › CD3E/correlation scatter plot between expression of CD3E and Neutrophils.pdf]

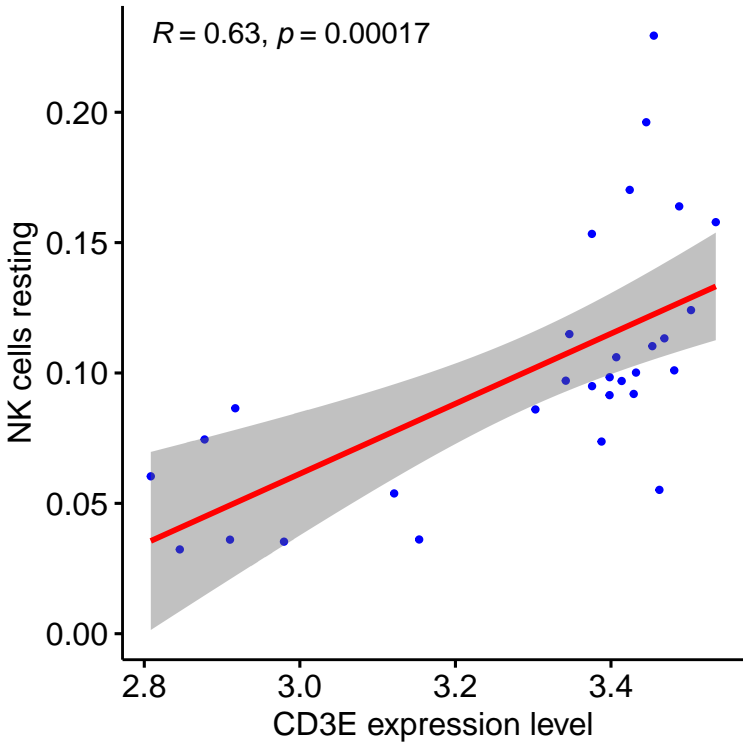

Supplement: Supplementary file 1 [file DataSheet1.ZIP › CD3E/correlation scatter plot between expression of CD3E and NK cells resting.pdf]

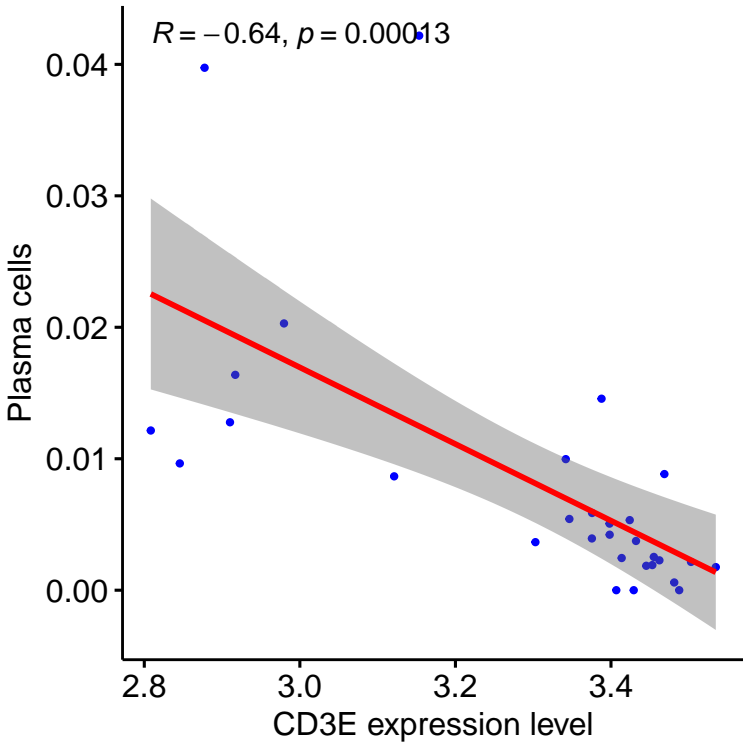

Supplement: Supplementary file 1 [file DataSheet1.ZIP › CD3E/correlation scatter plot between expression of CD3E and Plasma cells.pdf]

T cells CD4 memory activated

$R = -0.53, p = 0.0027$

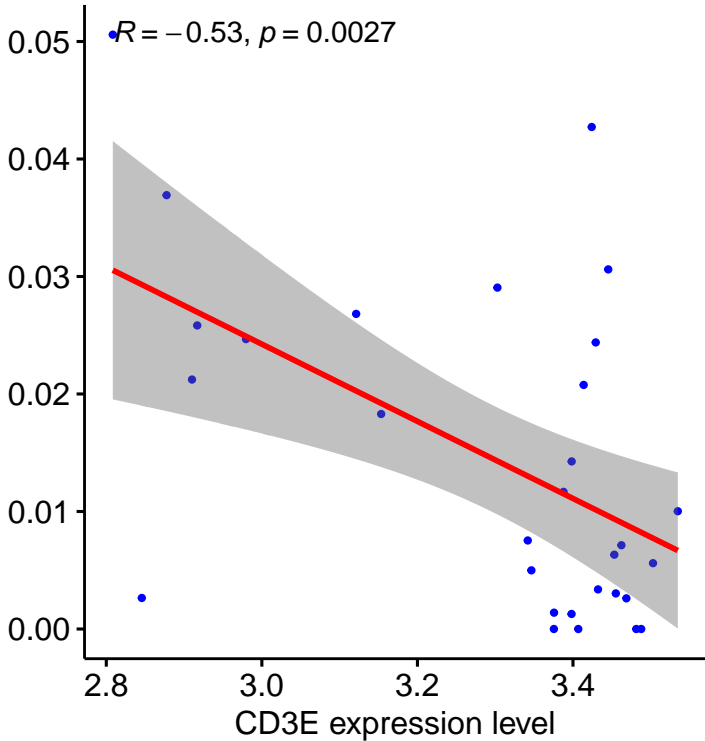

Supplement: Supplementary file 1 [file DataSheet1.ZIP › CD3E/correlation scatter plot between expression of CD3E and T cells CD4 memory activated.pdf]

T cells CD4 memory resting

$R = 0.64$ ,  $p = 0.00014$

0.20  
0.15  
0.10  
0.05  
0.00  
-0.05

2.8

3.0

3.2

3.4

CD3E expression level

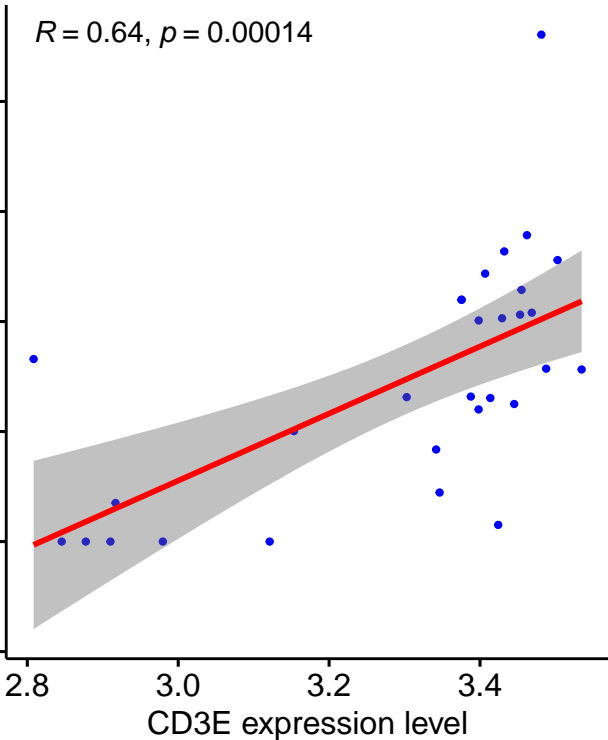

Supplement: Supplementary file 1 [file DataSheet1.ZIP › CD3E/correlation scatter plot between expression of CD3E and T cells CD4 memory resting.pdf]

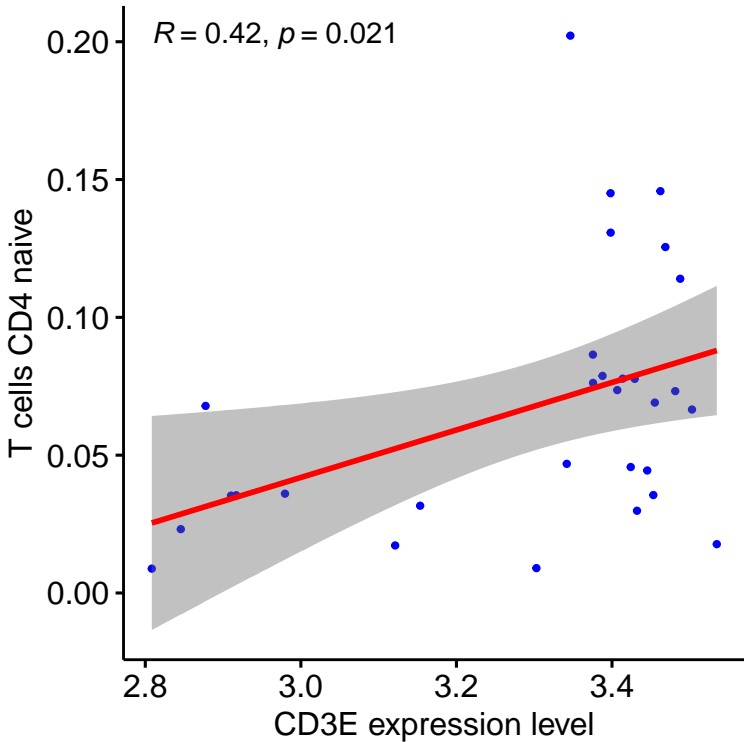

Supplement: Supplementary file 1 [file DataSheet1.ZIP › CD3E/correlation scatter plot between expression of CD3E and T cells CD4 naive.pdf]

$R = 0.83$ ,  $p = 1.3\text{e-}08$

T cells CD8

0.2

0.1

0.0

2.8

3.0

3.2

3.4

CD3E expression level

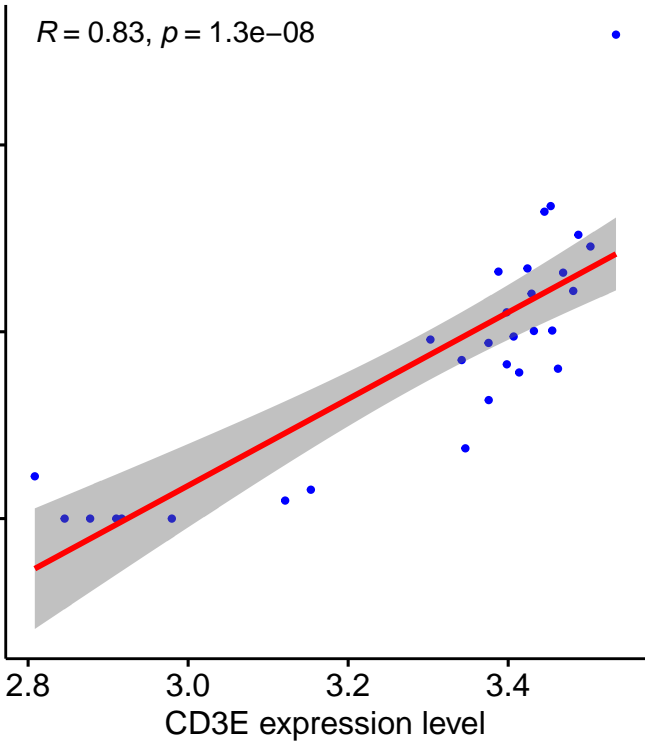

Supplement: Supplementary file 1 [file DataSheet1.ZIP › CD3E/correlation scatter plot between expression of CD3E and T cells CD8.pdf]

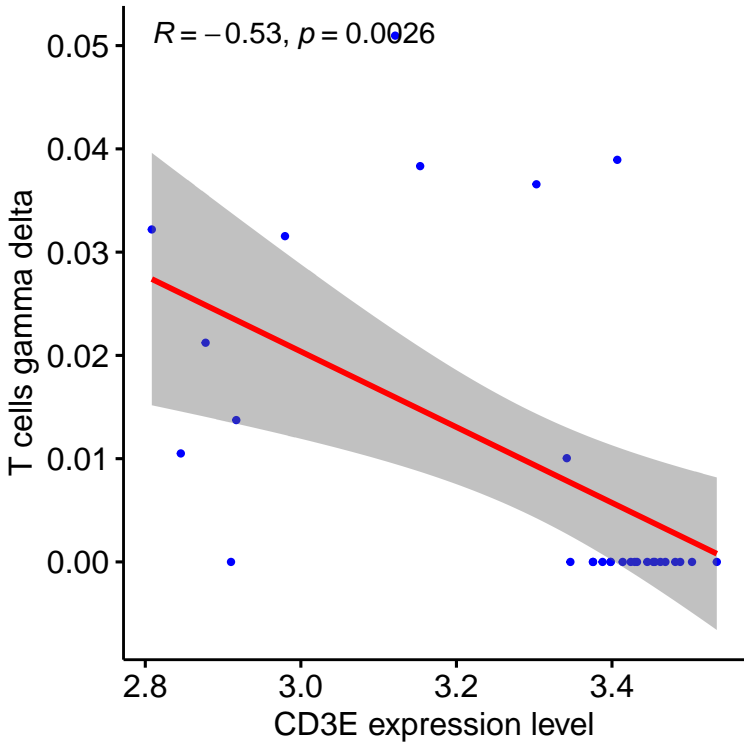

Supplement: Supplementary file 1 [file DataSheet1.ZIP › CD3E/correlation scatter plot between expression of CD3E and T cells gamma delta.pdf]

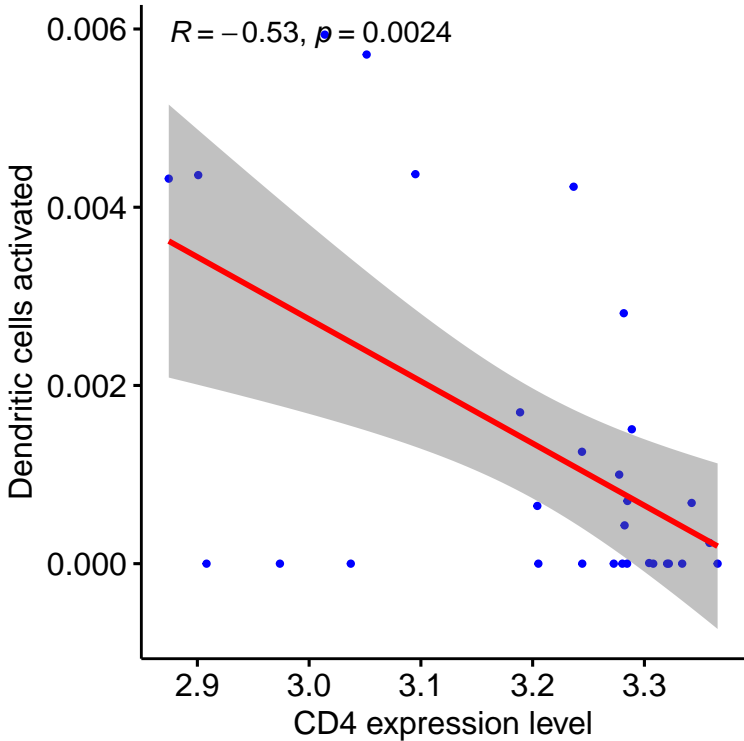

Supplement: Supplementary file 1 [file DataSheet1.ZIP › CD4/correlation scatter plot between expression of CD4 and Dendritic cells activated.pdf]

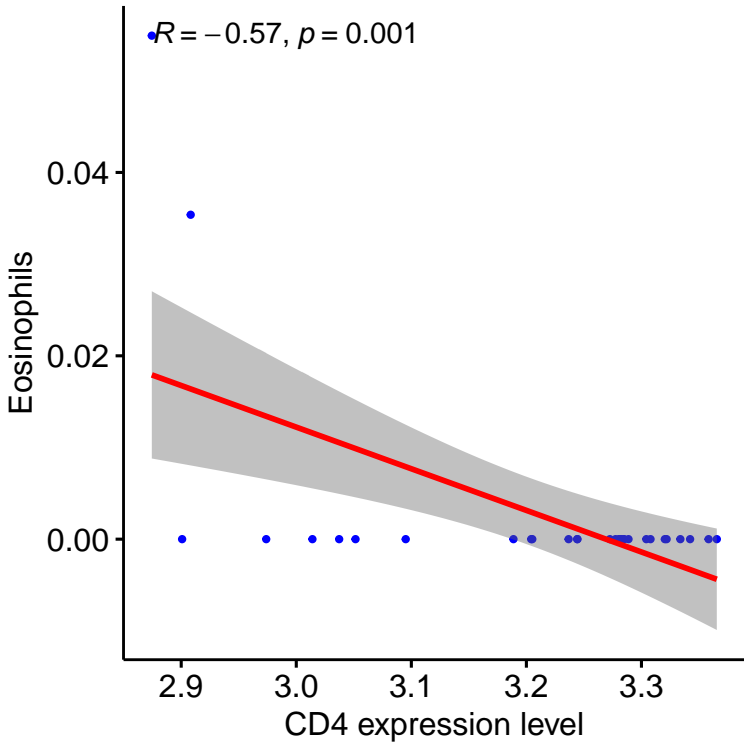

Supplement: Supplementary file 1 [file DataSheet1.ZIP › CD4/correlation scatter plot between expression of CD4 and Eosinophils.pdf]

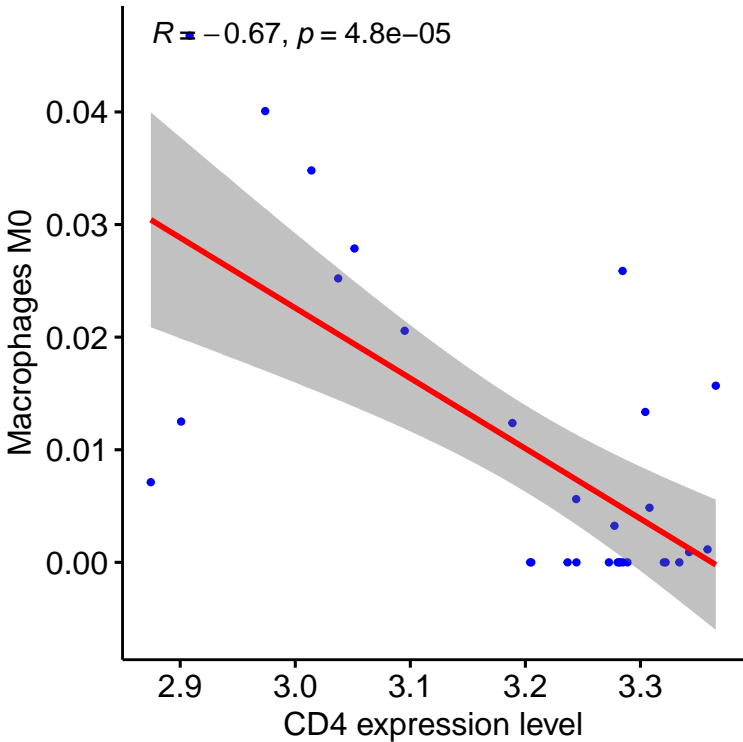

Supplement: Supplementary file 1 [file DataSheet1.ZIP › CD4/correlation scatter plot between expression of CD4 and Macrophages M0.pdf]

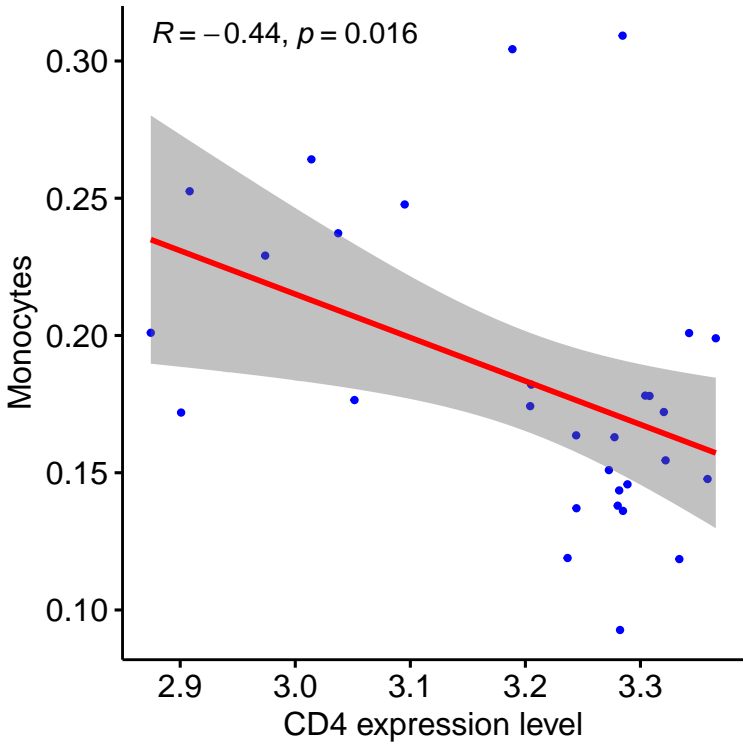

Supplement: Supplementary file 1 [file DataSheet1.ZIP › CD4/correlation scatter plot between expression of CD4 and Monocytes.pdf]

$R = -0.7$ ,  $p = 1.4e-05$

Neutrophils

0.6

0.5

0.3

2.9

3.0

3.1

3.2

3.3

CD4 expression level

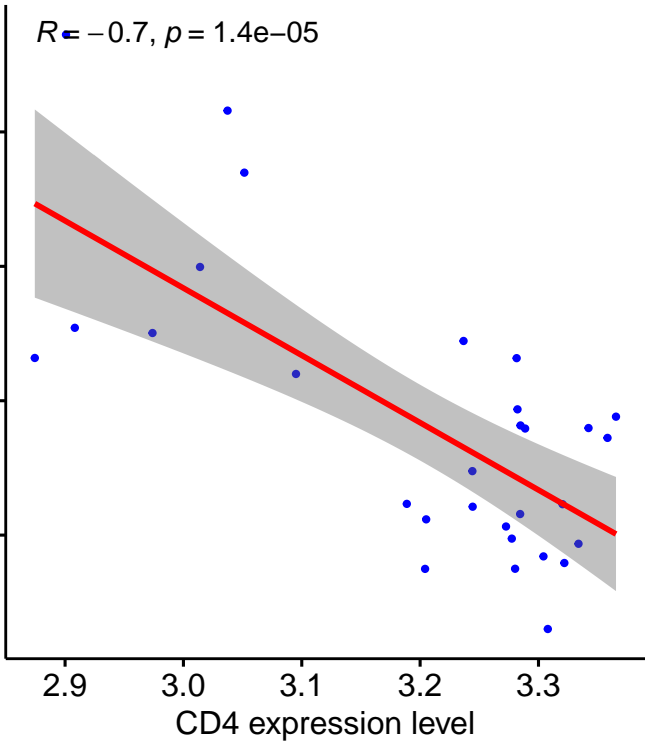

Supplement: Supplementary file 1 [file DataSheet1.ZIP › CD4/correlation scatter plot between expression of CD4 and Neutrophils.pdf]

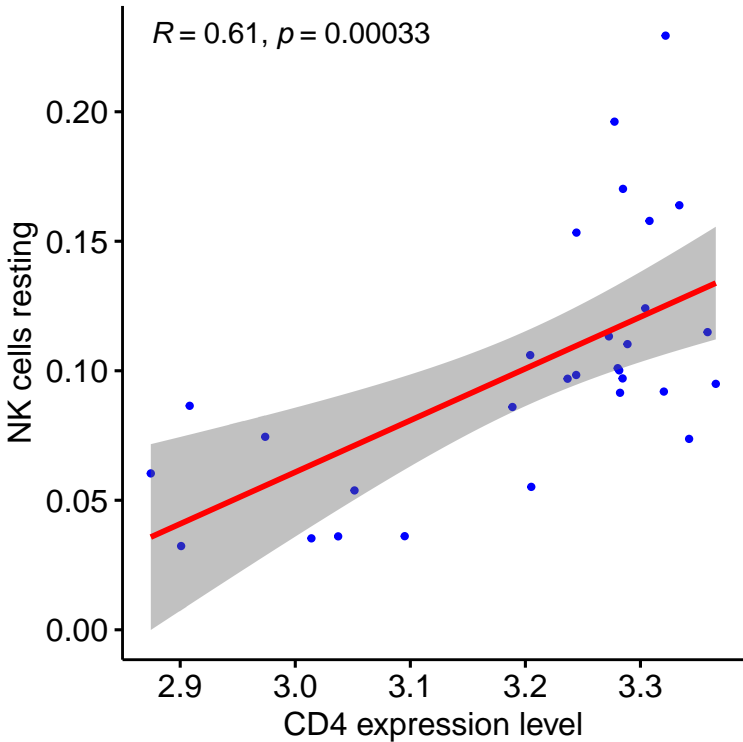

Supplement: Supplementary file 1 [file DataSheet1.ZIP › CD4/correlation scatter plot between expression of CD4 and NK cells resting.pdf]

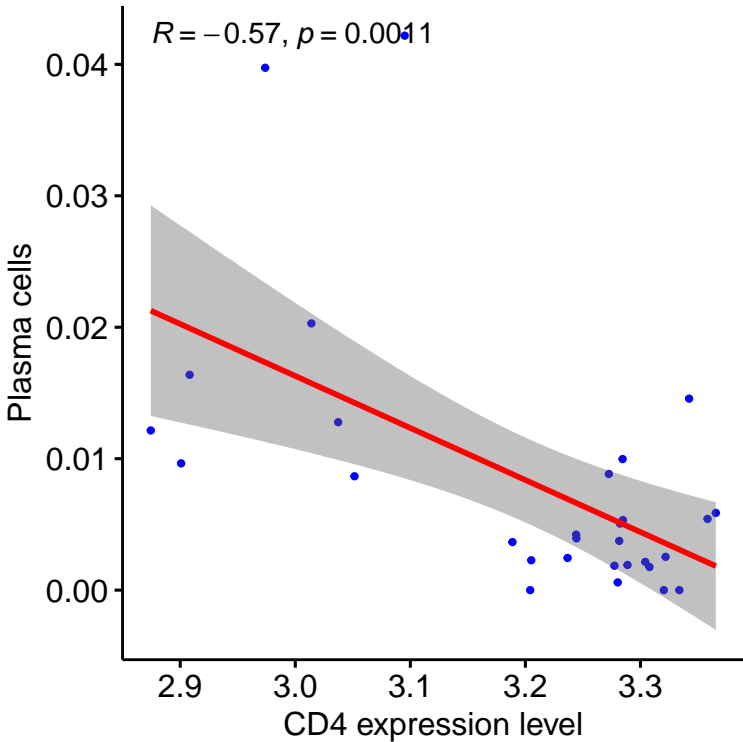

Supplement: Supplementary file 1 [file DataSheet1.ZIP › CD4/correlation scatter plot between expression of CD4 and Plasma cells.pdf]

T cells CD4 memory activated

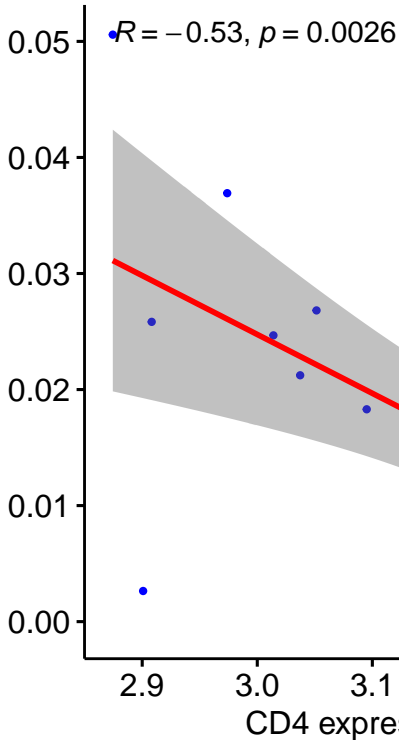

Supplement: Supplementary file 1 [file DataSheet1.ZIP › CD4/correlation scatter plot between expression of CD4 and T cells CD4 memory activated.pdf]

T cells CD4 memory resting

$R = 0.51, p = 0.0038$

0.20  
0.15  
0.10  
0.05  
0.00

2.9

3.0

3.1

3.2

3.3

CD4 expression level

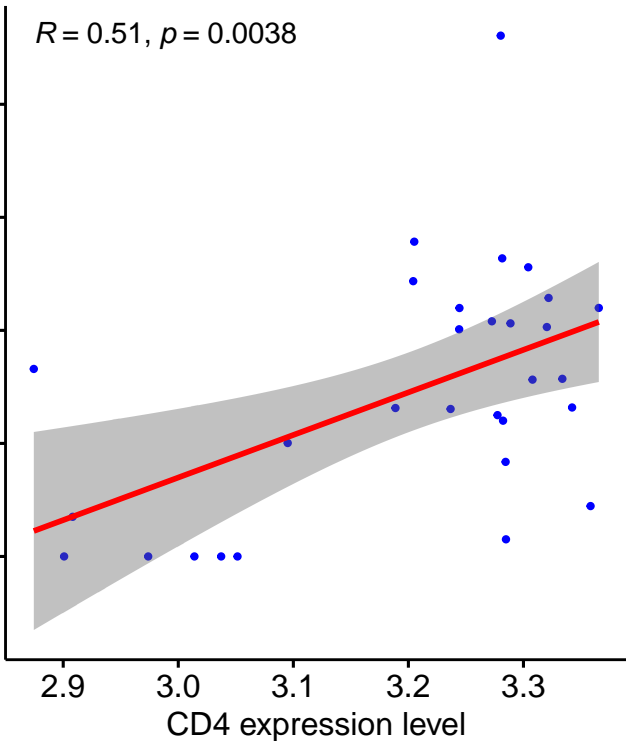

Supplement: Supplementary file 1 [file DataSheet1.ZIP › CD4/correlation scatter plot between expression of CD4 and T cells CD4 memory resting.pdf]

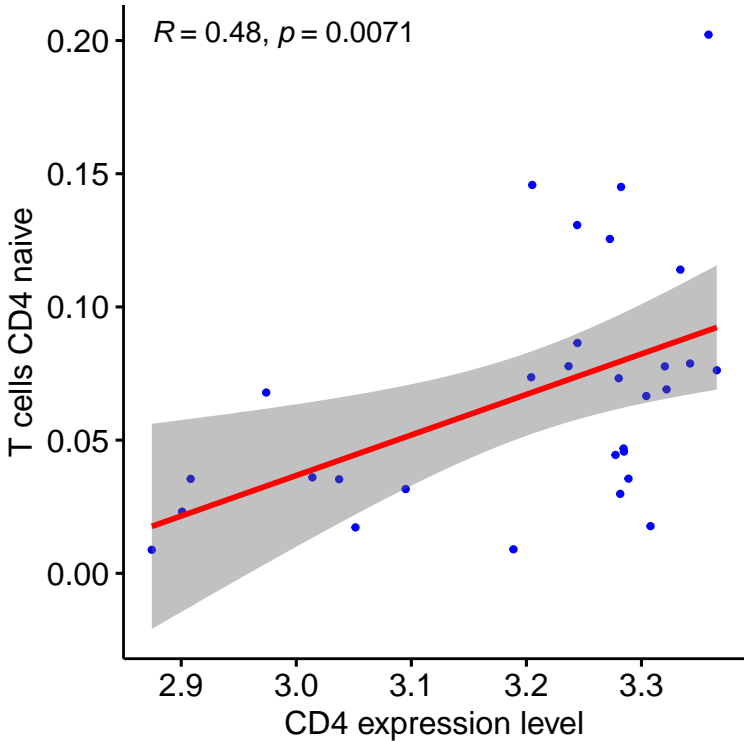

Supplement: Supplementary file 1 [file DataSheet1.ZIP › CD4/correlation scatter plot between expression of CD4 and T cells CD4 naive.pdf]

$R = 0.75$ ,  $p = 2.2\text{e-}06$

T cells CD8

0.2

0.1

0.0

2.9

3.0

3.1

3.2

3.3

CD4 expression level

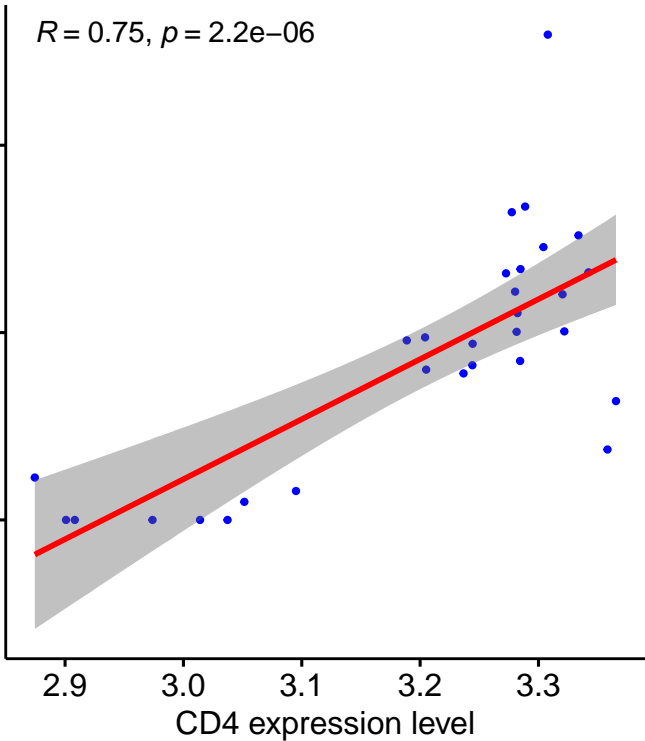

Supplement: Supplementary file 1 [file DataSheet1.ZIP › CD4/correlation scatter plot between expression of CD4 and T cells CD8.pdf]

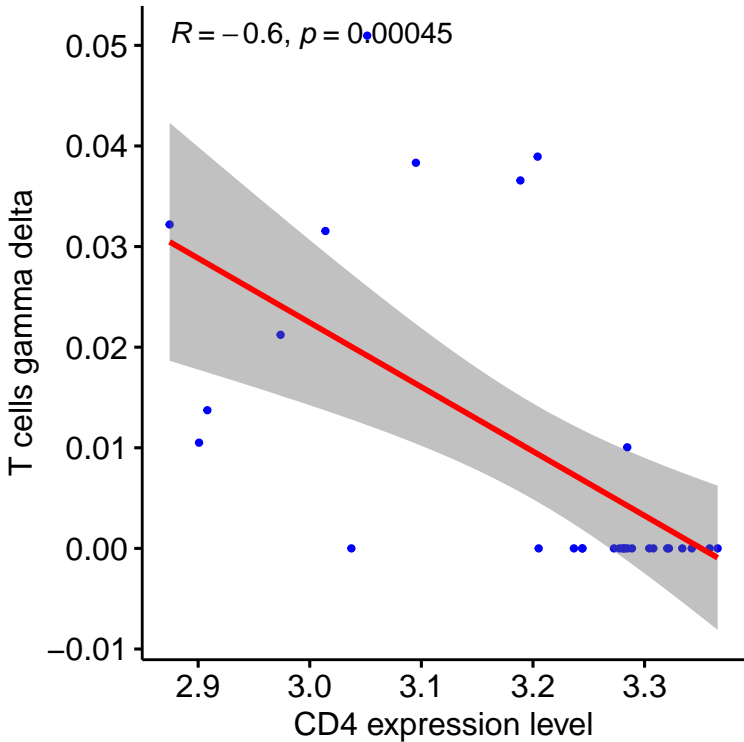

Supplement: Supplementary file 1 [file DataSheet1.ZIP › CD4/correlation scatter plot between expression of CD4 and T cells gamma delta.pdf]

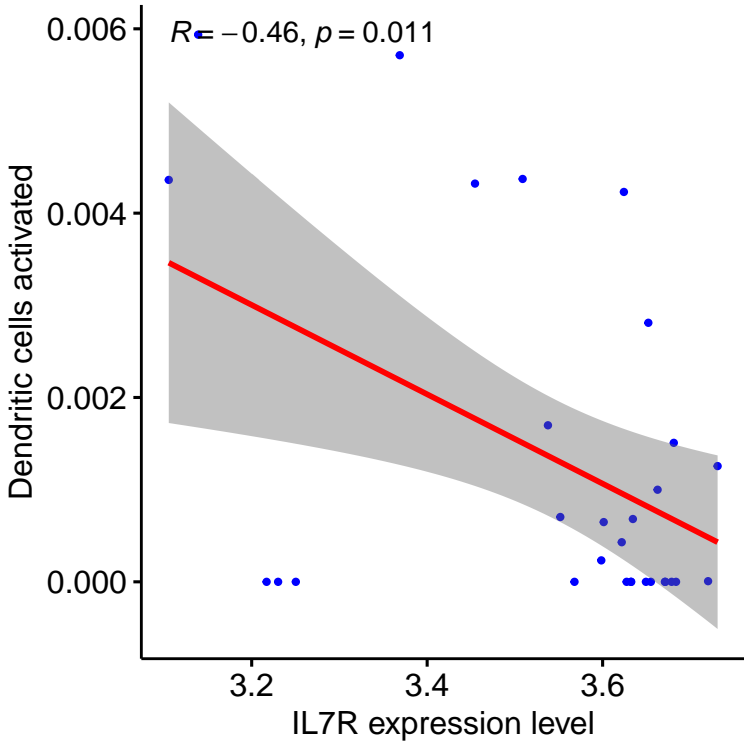

Supplement: Supplementary file 1 [file DataSheet1.ZIP › IL7R/correlation scatter plot between expression of IL7R and Dendritic cells activated.pdf]

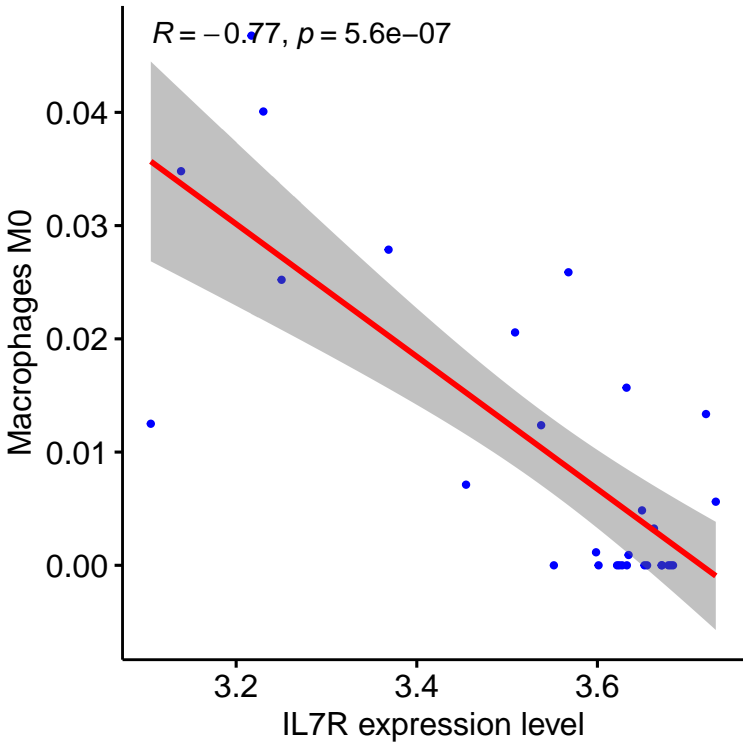

Supplement: Supplementary file 1 [file DataSheet1.ZIP › IL7R/correlation scatter plot between expression of IL7R and Macrophages M0.pdf]

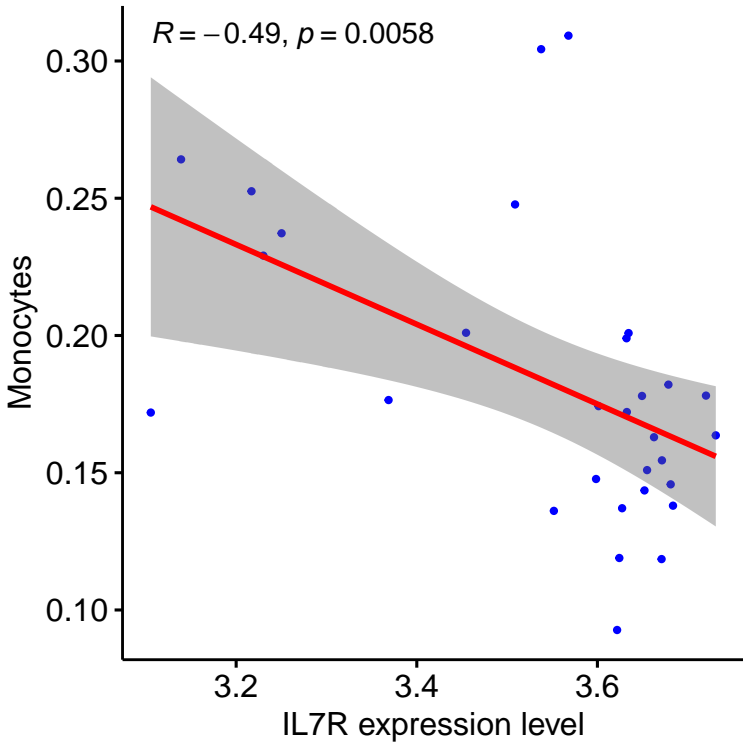

Supplement: Supplementary file 1 [file DataSheet1.ZIP › IL7R/correlation scatter plot between expression of IL7R and Monocytes.pdf]

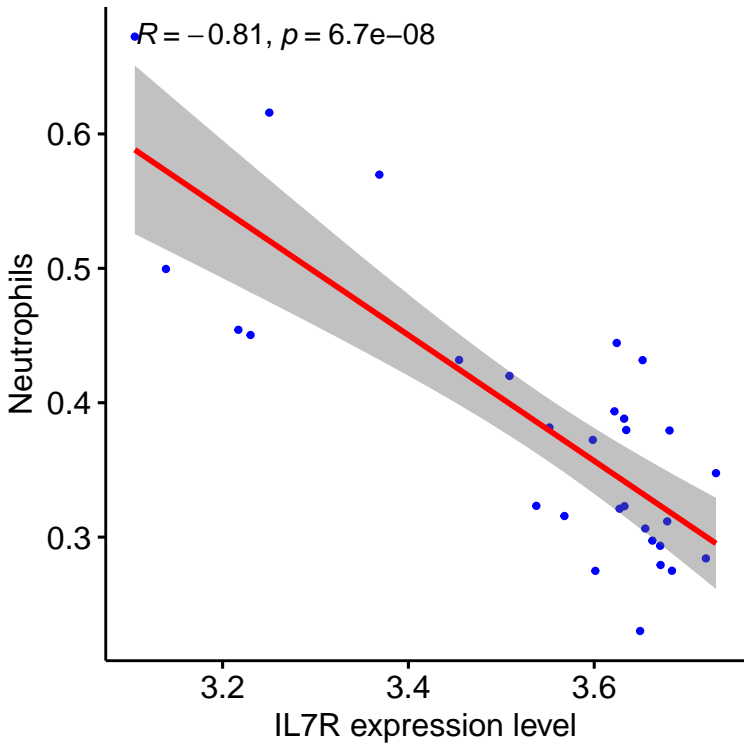

Supplement: Supplementary file 1 [file DataSheet1.ZIP › IL7R/correlation scatter plot between expression of IL7R and Neutrophils.pdf]

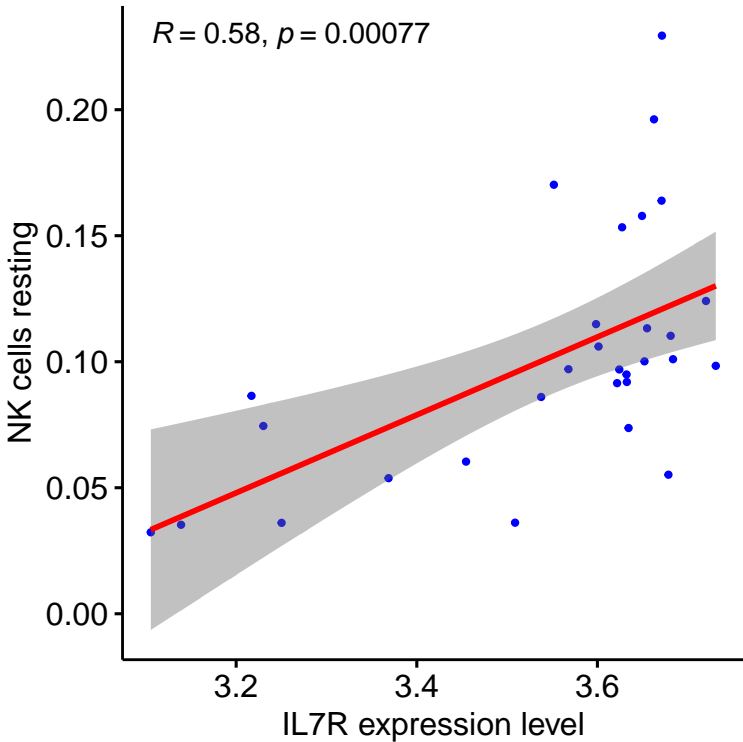

Supplement: Supplementary file 1 [file DataSheet1.ZIP › IL7R/correlation scatter plot between expression of IL7R and NK cells resting.pdf]

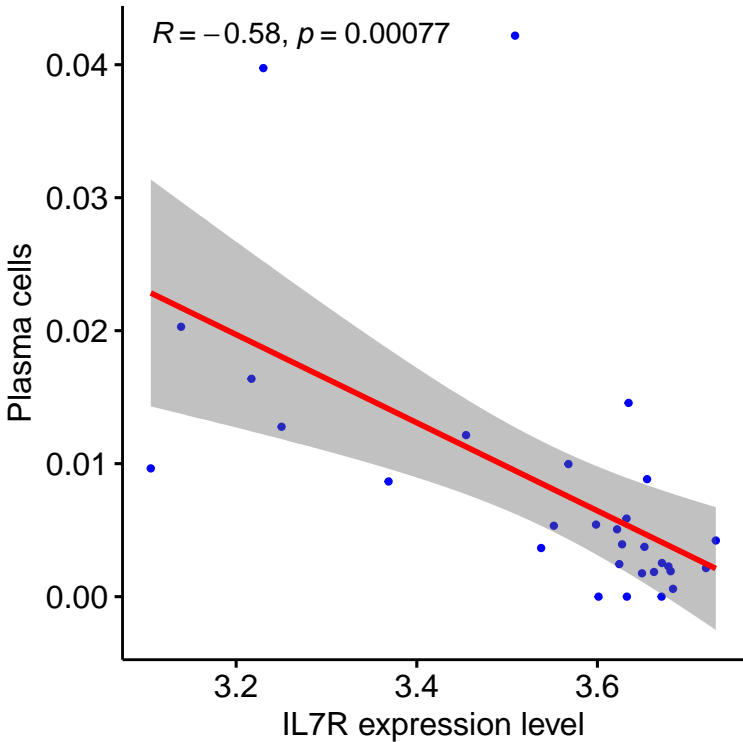

Supplement: Supplementary file 1 [file DataSheet1.ZIP › IL7R/correlation scatter plot between expression of IL7R and Plasma cells.pdf]

T cells CD4 memory activated

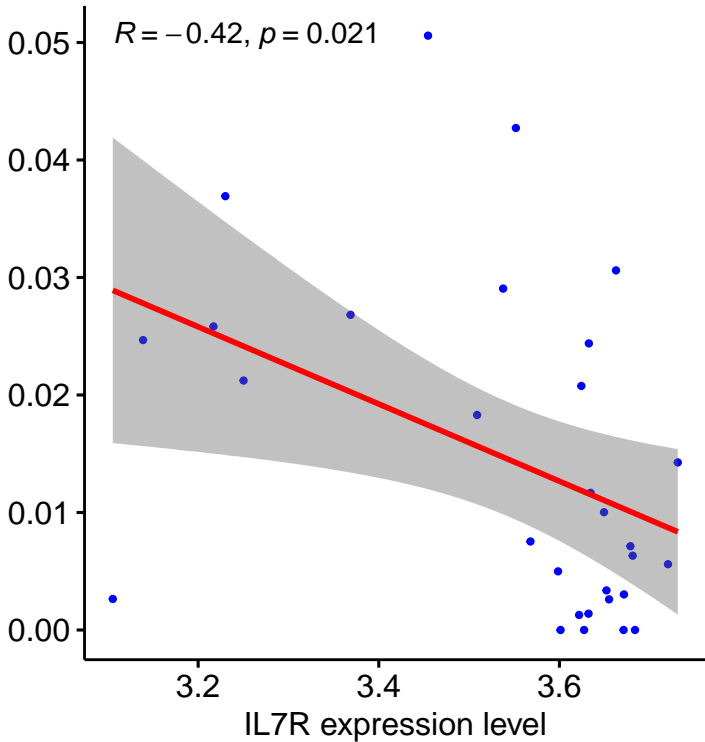

Supplement: Supplementary file 1 [file DataSheet1.ZIP › IL7R/correlation scatter plot between expression of IL7R and T cells CD4 memory activated.pdf]

$R = 0.73$ ,  $p = 3.9\text{e-}06$

T cells CD4 memory resting

0.2

0.1

0.0

3.2

3.4

3.6

IL7R expression level

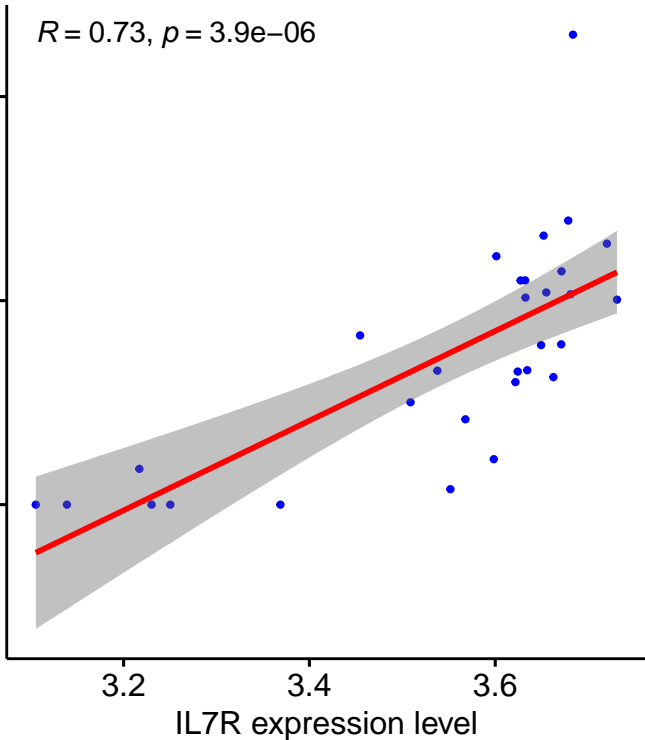

Supplement: Supplementary file 1 [file DataSheet1.ZIP › IL7R/correlation scatter plot between expression of IL7R and T cells CD4 memory resting.pdf]

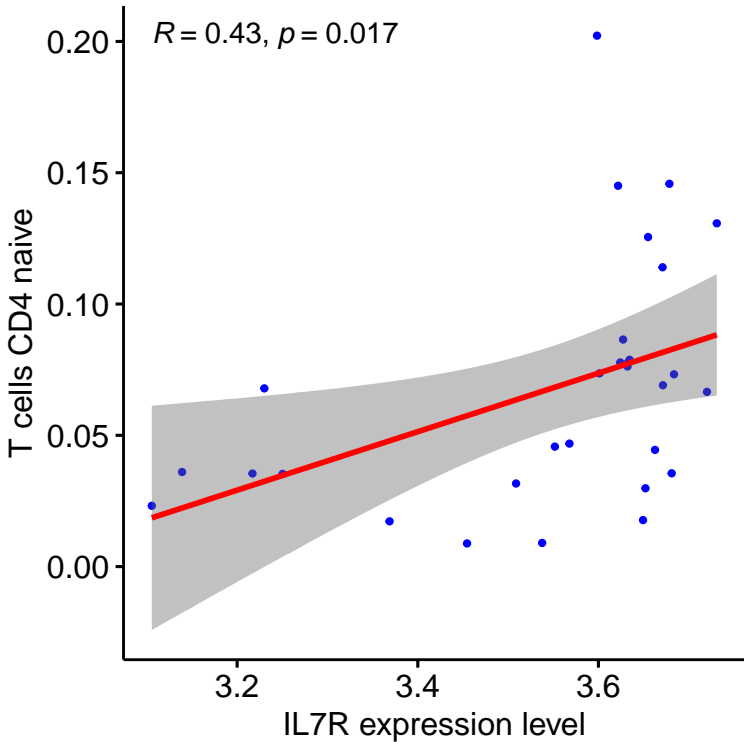

Supplement: Supplementary file 1 [file DataSheet1.ZIP › IL7R/correlation scatter plot between expression of IL7R and T cells CD4 naive.pdf]

$R = 0.76$ ,  $p = 1.4\text{e-}06$

T cells CD8

0.2

0.1

0.0

3.2

3.4

3.6

IL7R expression level

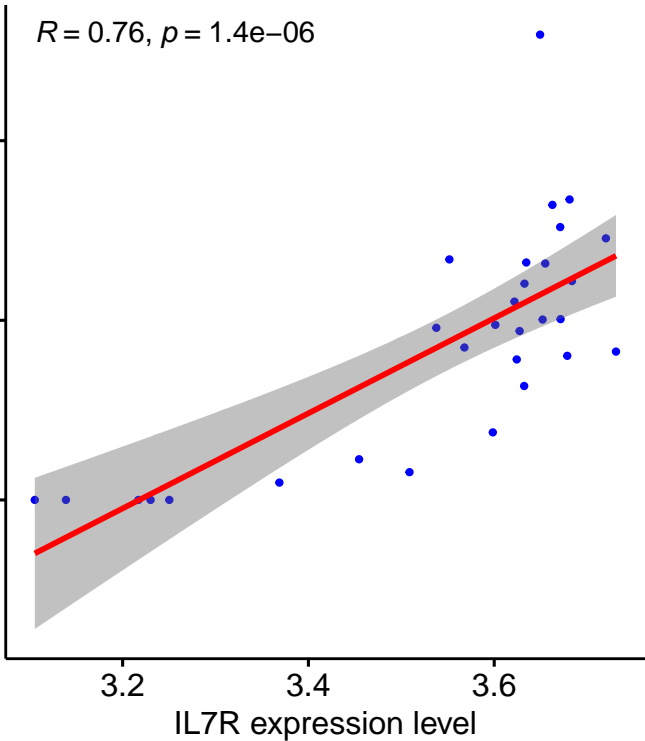

Supplement: Supplementary file 1 [file DataSheet1.ZIP › IL7R/correlation scatter plot between expression of IL7R and T cells CD8.pdf]

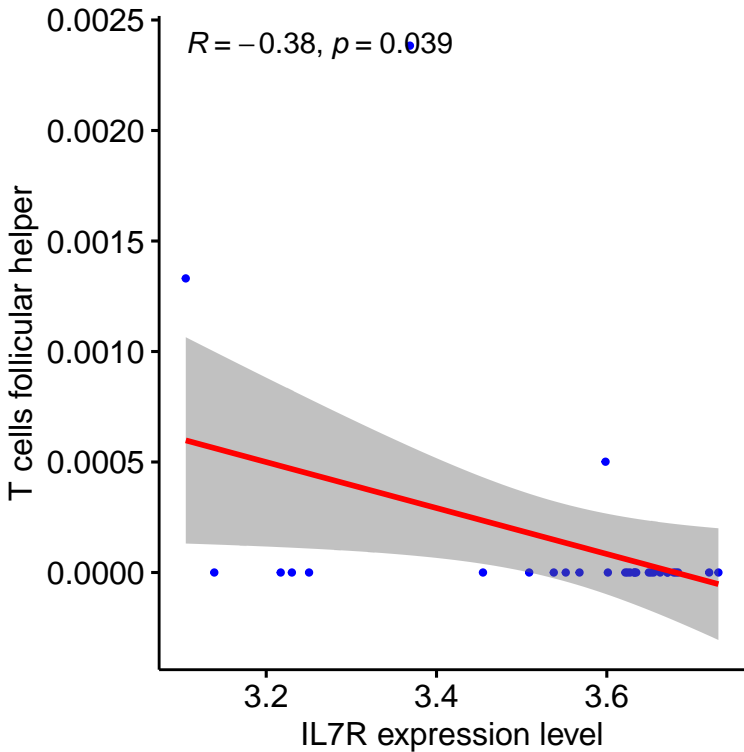

Supplement: Supplementary file 1 [file DataSheet1.ZIP › IL7R/correlation scatter plot between expression of IL7R and T cells follicular helper.pdf]

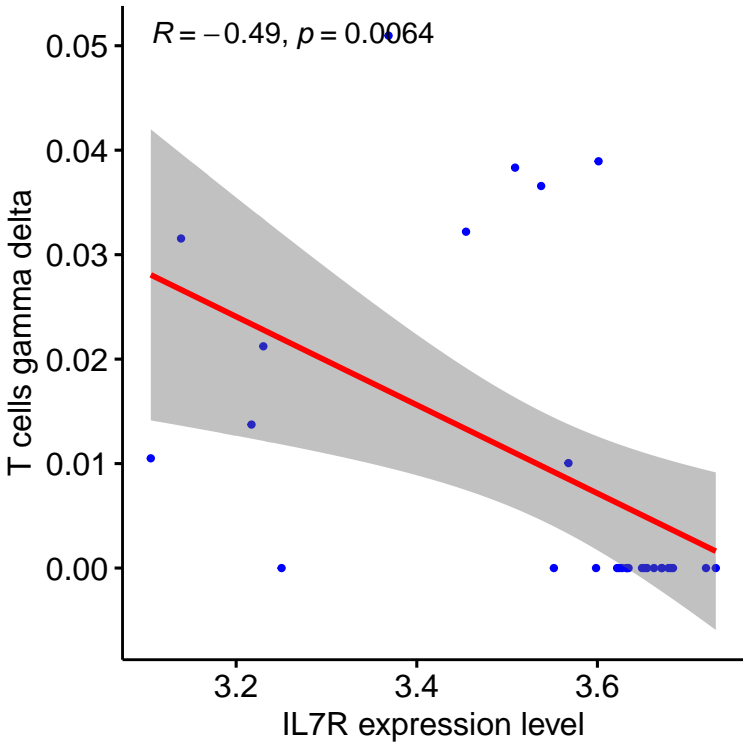

Supplement: Supplementary file 1 [file DataSheet1.ZIP › IL7R/correlation scatter plot between expression of IL7R and T cells gamma delta.pdf]

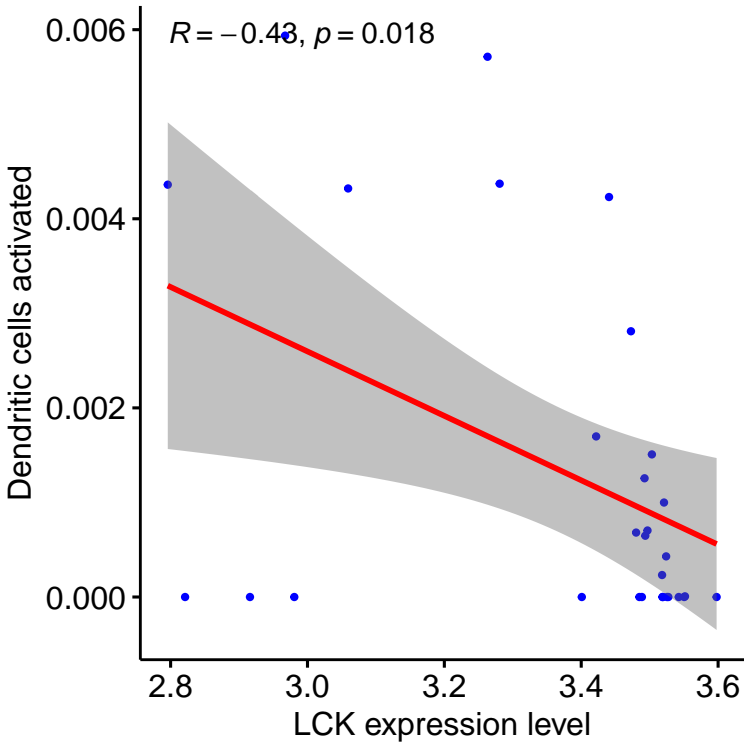

Supplement: Supplementary file 1 [file DataSheet1.ZIP › LCK/correlation scatter plot between expression of LCK and Dendritic cells activated.pdf]

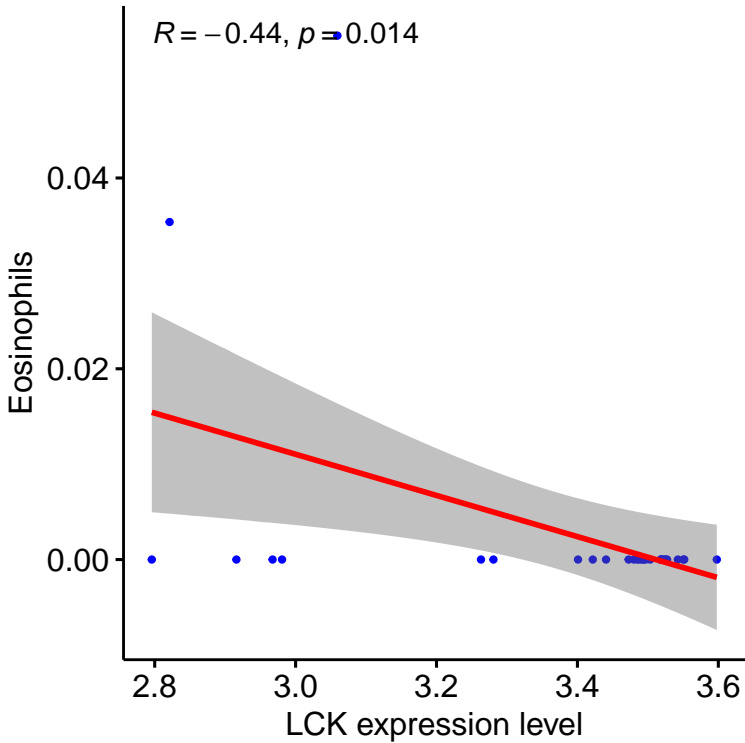

Supplement: Supplementary file 1 [file DataSheet1.ZIP › LCK/correlation scatter plot between expression of LCK and Eosinophils.pdf]

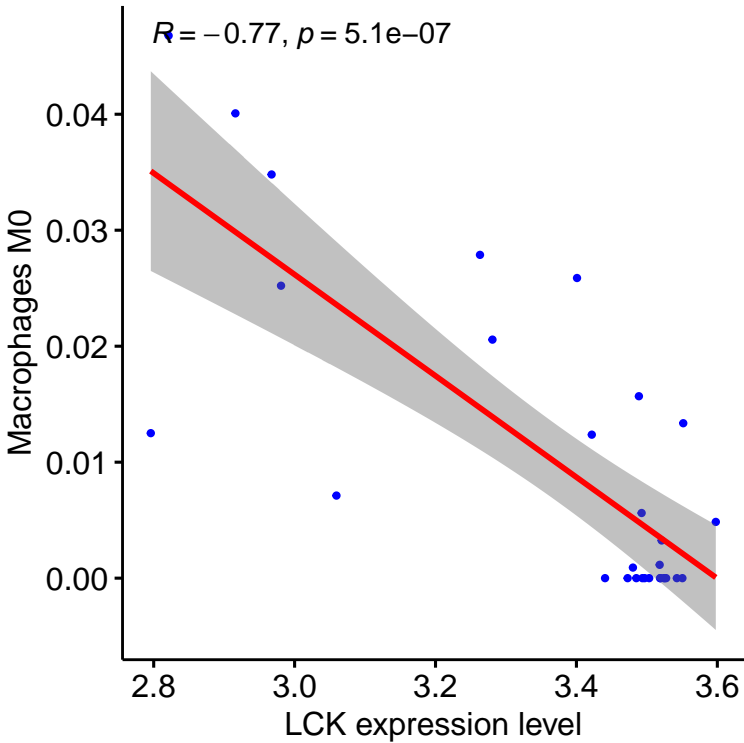

Supplement: Supplementary file 1 [file DataSheet1.ZIP › LCK/correlation scatter plot between expression of LCK and Macrophages M0.pdf]

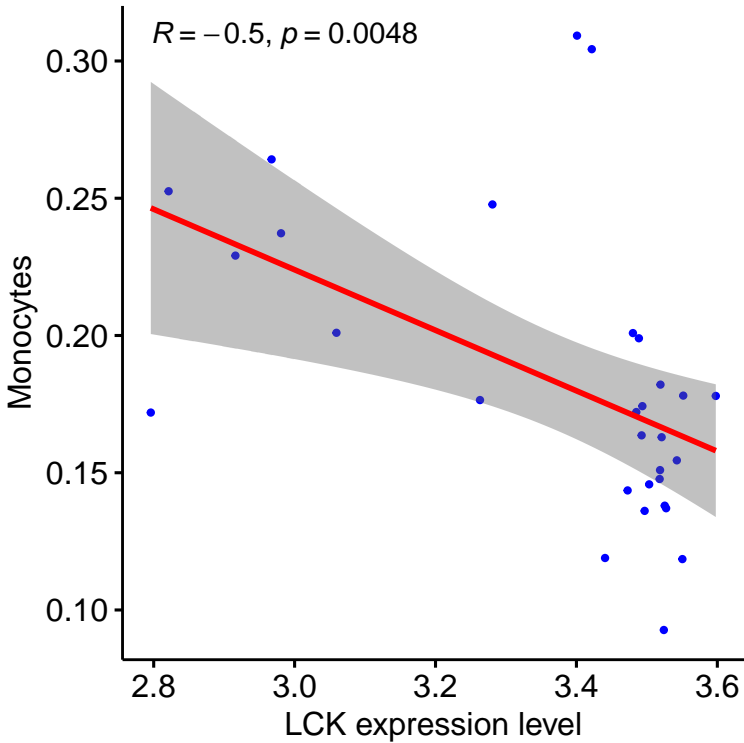

Supplement: Supplementary file 1 [file DataSheet1.ZIP › LCK/correlation scatter plot between expression of LCK and Monocytes.pdf]

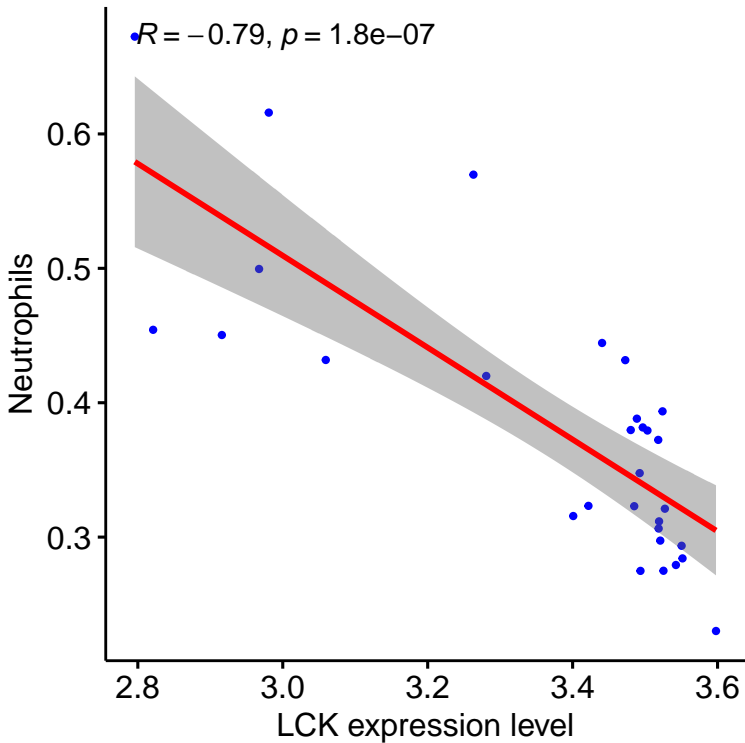

Supplement: Supplementary file 1 [file DataSheet1.ZIP › LCK/correlation scatter plot between expression of LCK and Neutrophils.pdf]

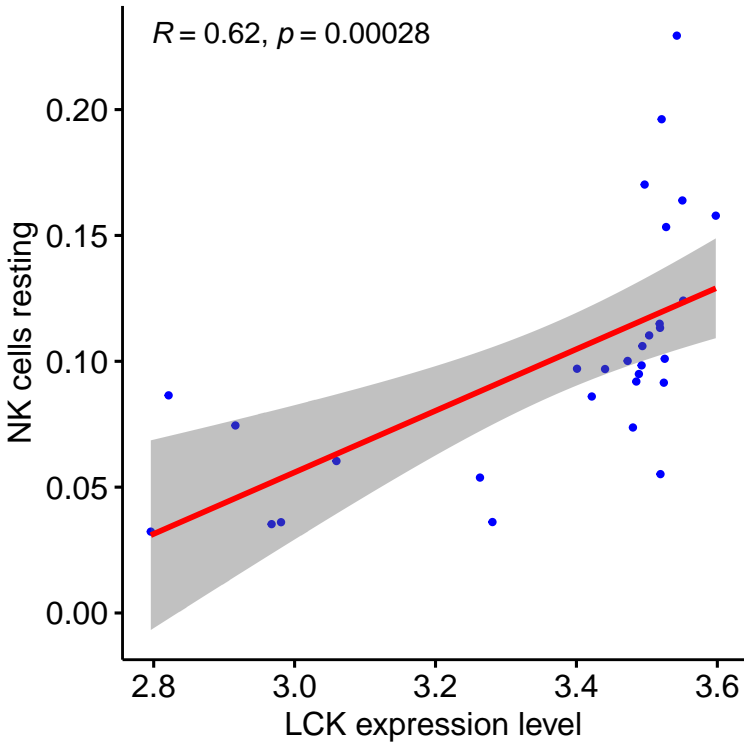

Supplement: Supplementary file 1 [file DataSheet1.ZIP › LCK/correlation scatter plot between expression of LCK and NK cells resting.pdf]

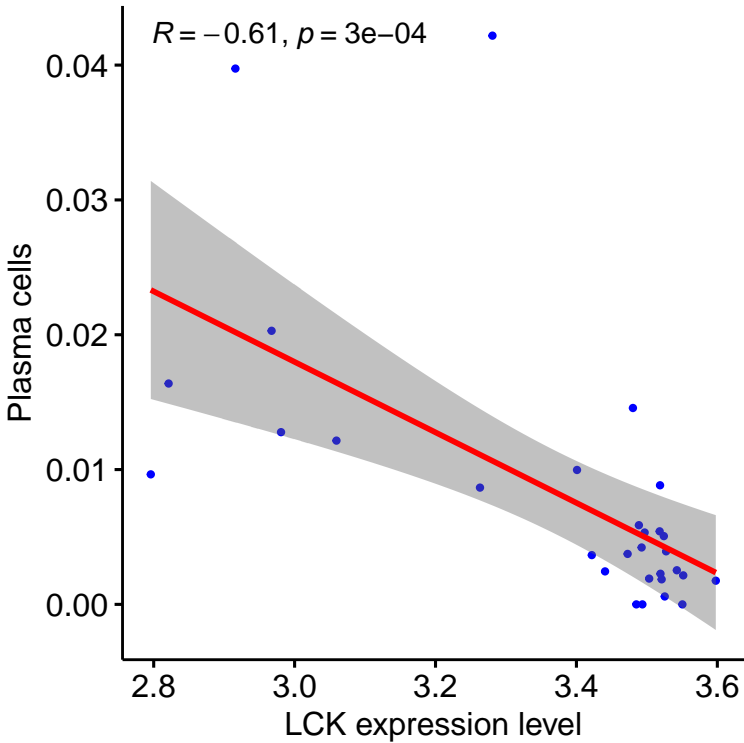

Supplement: Supplementary file 1 [file DataSheet1.ZIP › LCK/correlation scatter plot between expression of LCK and Plasma cells.pdf]

T cells CD4 memory activated

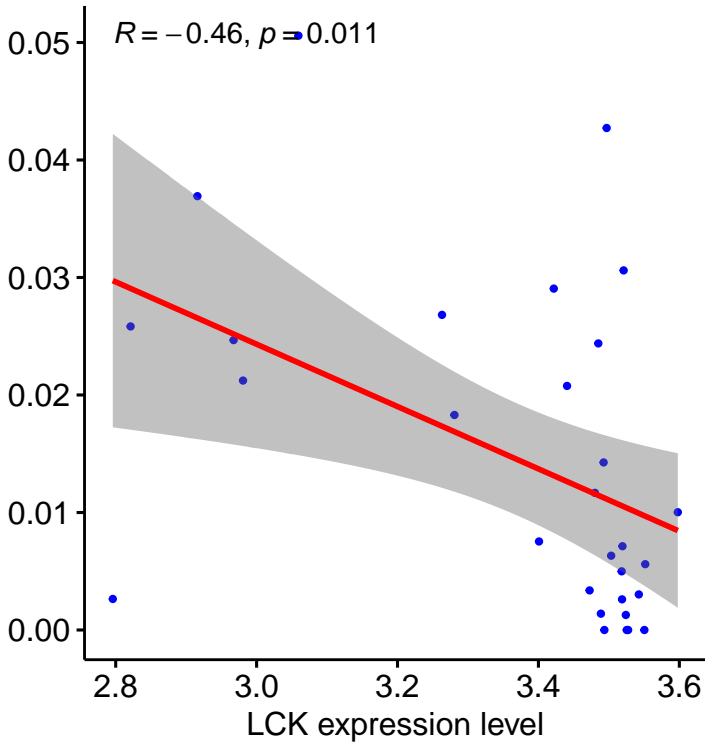

Supplement: Supplementary file 1 [file DataSheet1.ZIP › LCK/correlation scatter plot between expression of LCK and T cells CD4 memory activated.pdf]

T cells CD4 memory resting

$R = 0.63$ ,  $p = 0.00018$

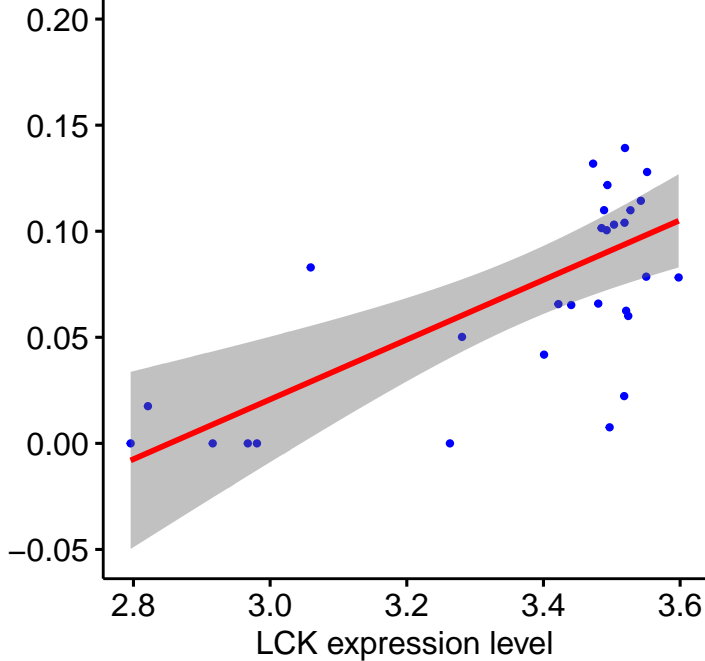

Supplement: Supplementary file 1 [file DataSheet1.ZIP › LCK/correlation scatter plot between expression of LCK and T cells CD4 memory resting.pdf]

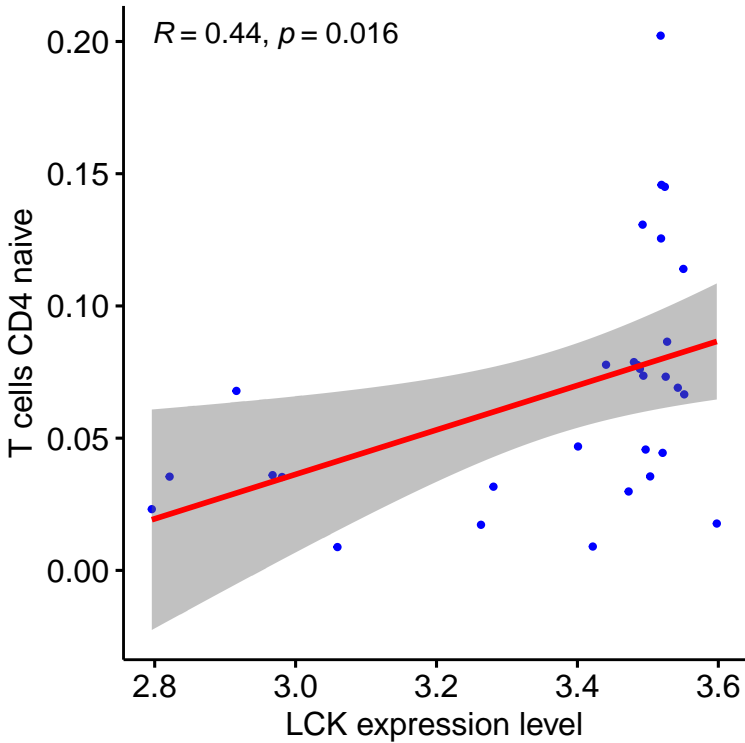

Supplement: Supplementary file 1 [file DataSheet1.ZIP › LCK/correlation scatter plot between expression of LCK and T cells CD4 naive.pdf]

$R = 0.79$ ,  $p = 2.3\text{e-}07$

T cells CD8

0.2

0.1

0.0

2.8

3.0

3.2

3.4

3.6

LCK expression level

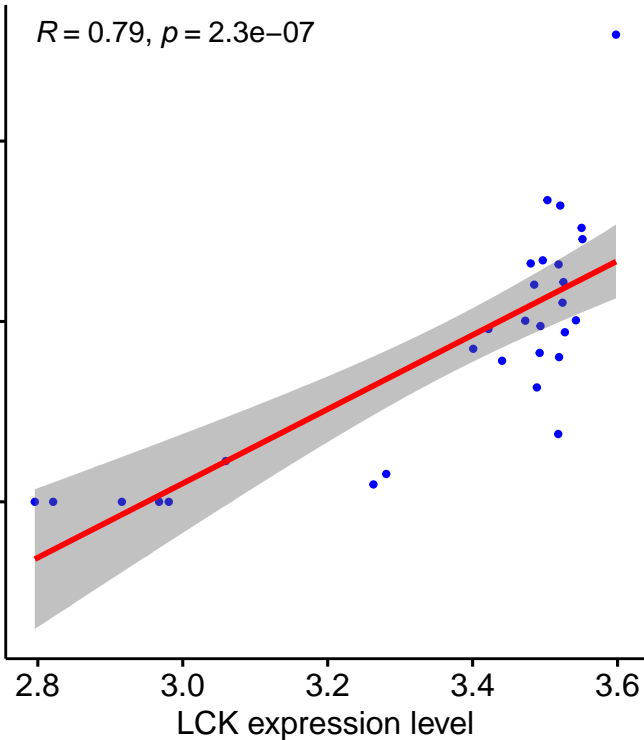

Supplement: Supplementary file 1 [file DataSheet1.ZIP › LCK/correlation scatter plot between expression of LCK and T cells CD8.pdf]

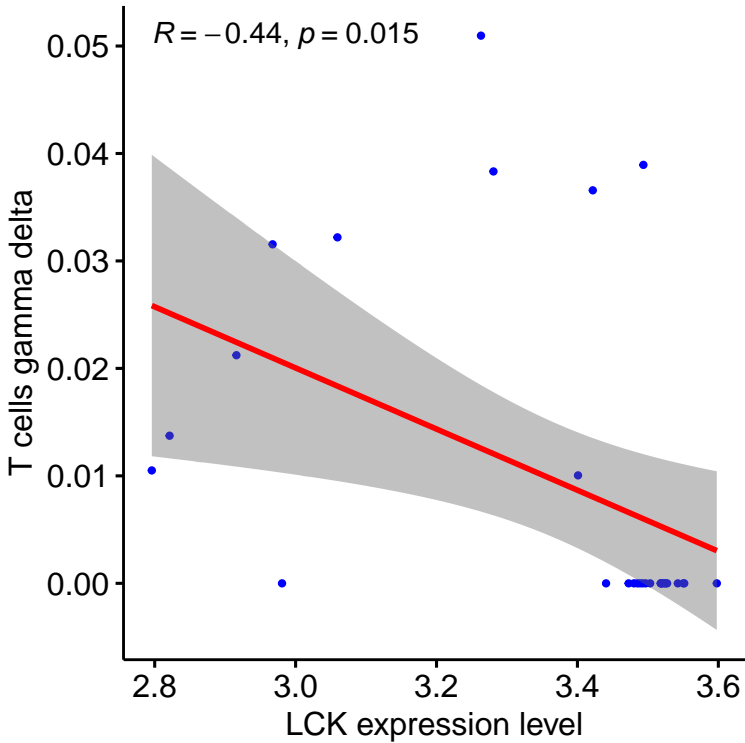

Supplement: Supplementary file 1 [file DataSheet1.ZIP › LCK/correlation scatter plot between expression of LCK and T cells gamma delta.pdf]

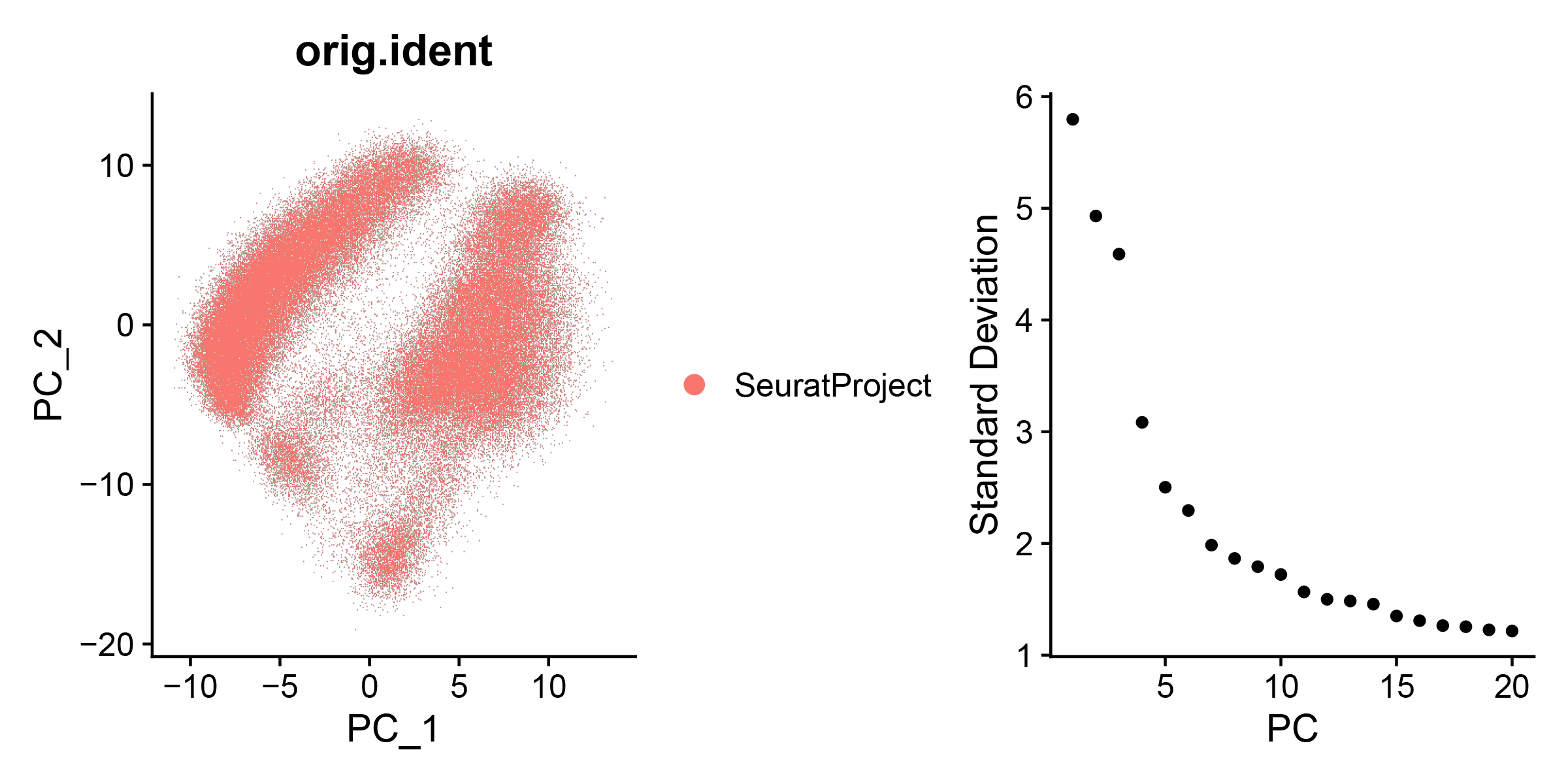

Supplement: Supplementary file 2 [file Image1.TIF]
